# Design, Synthesis, and Evaluation of New Polyhydroxylated *B* *is*‐Chalcones as Potential COX‐2 Selective Inhibitors

**DOI:** 10.1002/cmdc.202500784

**Published:** 2026-04-25

**Authors:** Rui Pereira, Alberto N. Araújo, Daniela Ribeiro, Ismael Rufino, Nuno Martinho, Rita C. Guedes, Vera L. M. Silva, Eduarda Fernandes

**Affiliations:** ^1^ LAQV‐REQUIMTE Laboratory of Applied Chemistry Department of Chemical Sciences Faculty of Pharmacy University of Porto Porto Portugal; ^2^ Institute of Agricultural and Environmental Research and Technology Faculty of Agrarian Sciences and Environment University of the Azores Angra do Heroísmo Portugal; ^3^ Research Institute for Medicines (iMed.ULisboa) Faculty of Pharmacy University of Lisboa Lisboa Portugal; ^4^ LAQV‐REQUIMTE Department of Chemistry University of Aveiro Aveiro Portugal

**Keywords:** *bis*‐chalcones, human cyclooxygenase‐2 inhibitors, inhibition type, molecular docking, synthesis

## Abstract

Selective inhibition of COX‐2 is considered one of the best strategies for treating chronic inflammatory diseases. However, the currently available options still have significant side effects due to exacerbated selectivity. *Bis*‐chalcone derivatives have shown promising anti‐inflammatory properties with reduced side effects. In this study, a family of polyhydroxylated *bis*‐chalcones was synthesized and tested in vitro for their ability to inhibit human COX‐2 and COX‐1 and to assess selectivity. To further understand their mechanism of action, inhibitory kinetic analysis and in silico molecular docking calculations were performed. The results showed that *bis*‐chalcone **31**, with hydroxy groups at positions 3′ and 4′ of the B rings and three hydroxy groups at the center, was the most active. It was recognized as a mixed‐type inhibitor with balanced selectivity. With molecular docking, it was observed that this substitution pattern provided *bis*‐chalcone **31** with additional bulk that hindered its access to the active pocket of COX‐1 over COX‐2. Also, compound **31** establishes additional hydrogen bonds within the COX‐2 pocket that *bis*‐chalcone **30** did not, therefore explaining the selectivity and superior potency of *bis*‐chalcone **31**. In conclusion, *bis*‐chalcone **31** with multiple hydroxy groups in its structure shows promising properties for the design of new COX‐2 selective inhibitors.

## Graphical abstract

Novel polyhydroxylated *bis*‐chalcones were successfully synthesized and demonstrated COX‐2 selective inhibition. The most potent and selective inhibitor exhibited a mixed‐type inhibition mechanism with COX‐2. Its additional bulk preferentially blocked the access to the active pocket of COX‐1 rather than COX‐2.

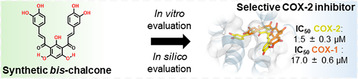

## 1 Introduction

Many health issues in developed nations, such as arthritis or cardiovascular disease, and leading causes of morbidity and mortality like diabetes or cancer, stem from persistent inflammation [1]. This condition translates to a physiological imbalance due to an overproduction of pro‐inflammatory mediators such as cytokines and prostaglandins, and inadequate anti‐inflammatory mediator levels. An effective therapeutic approach is, in turn, challenging due to the multiple dysregulated pro‐inflammatory mechanisms causing chronic inflammation [1].

A well‐studied pro‐inflammatory mechanism arises from high cyclooxygenase activity, which results in the overproduction of prostaglandins (PGs) from arachidonic acid [2, 3]. Of the various cyclooxygenase (COX) enzymes, the COX‐2 isoform, primarily located in the endoplasmic reticulum, is substantially expressed during inflammation. It produces eicosanoids, which account for the prolongation of the pro‐inflammatory response as well as the cardinal signs of inflammation. Therefore, inhibiting only this isoform seems preferable as it prevents secondary effects caused by the inhibition of other COX enzymes. Specifically, COX‐1 is responsible for the biosynthesis of PGs PGE_2_ and PGI_2_ in the gastrointestinal tissues, and both exert multiple cytoprotective effects on gastric function, including bicarbonate and mucus secretion, decrease of gastric acid and pepsin production, and maintenance of optimal blood flow to the mucosa [4]. Avoiding inhibition of this isoform while decreasing the COX‐2 activity minimizes gastrointestinal complications.

Despite their selectivity, COX‐2 inhibitors currently available still carry severe side effects, notably cardiovascular diseases and renal ischemia [5]. Researchers attribute these side effects to an imbalance between different eicosanoids. Findings reveal that COX‐2 is a constitutive enzyme in brain and kidney tissue, causing complications when its activity is blocked [5, 6]. A key problem with selective COX‐2 inhibitors is their ability to halt production of the essential vasodilator prostacyclin (PGI_2_), in contrast to thromboxane A2 (TXA_2_), a pro‐aggregatory and vasoconstrictive mediator whose production is regulated by COX‐1 and remains unaffected [6]. This imbalance between TXA_2_ and PGI_2_ can ultimately lead to heart attacks and strokes. Therefore, alternatives are needed.

Plants, a key part of nature, offer a vast array of compounds useful for medicine and are a source of inspiration for the development of novel therapeutic drugs. Widely found in plants, chalcones have been known and studied extensively for over a century. Originally mentioned by Stanisław Kostanecki and Josef Tambor in 1899 [4, 7], these natural compounds of bronze and yellow color are associated with versatile and potent bioactivities, such as anti‐inflammatory [3], antioxidant [8], and anticancer [9], with the most active compounds featuring hydroxy substituent groups as the main feature. Some chalcones, such as choleretic metochalcone and sofalcone, were meanwhile approved for clinical applications as gastroprotective drugs. For anti‐inflammatory activity, many chalcone derivatives described in the literature have shown exceptional capabilities acting through different pro‐inflammatory mechanisms to prevent chronic inflammation [3]. One of those mechanisms was indeed the inhibition of PGs production, by either targeting the COX‐2 enzyme or its expression [3], accompanied by the fact that chalcones show reduced cytotoxicity and side effects [10, 11, 12, 13].

Recently, new compounds derived from chalcones have attracted interest, such as dihydrochalcones [14] and hybrid chalcones, due to newly found biopotential [15, 16, 17]. *Bis*‐chalcones are another class of chalcone derivatives that have garnered considerable attention in the last two decades and are composed of two chalcone moieties in the same structure [18]. In fact, some studies suggest that these derivatives outperform regular chalcones in certain biological activities and selectivity, particularly as anti‐inflammatory agents, although few *bis*‐chalcone structures have been investigated for this purpose [18]. For selective COX‐2 inhibition, only one example is available in the literature, from the El‐Sabbagh et al. group [19]. Despite the potential therapeutic application, there is a clear lack of information on the range of active structures.

Taking this into account, we aimed to produce new polyhydroxylated *bis*‐chalcone derivatives, evaluate their anti‐inflammatory properties as selective COX‐2 inhibitors, and establish structure–activity relationships. In vitro assays were performed to assess this capability as well as the COX‐2/COX‐1 selectivity, complemented by calculations of inhibition type and docking studies. Ultimately, our goal is to gather enough information to identify the most active *bis*‐chalcone structure and determine its preferred mode of action.

## 2 Results and Discussion

### 2.1 Chemistry

**SCHEME 1:**
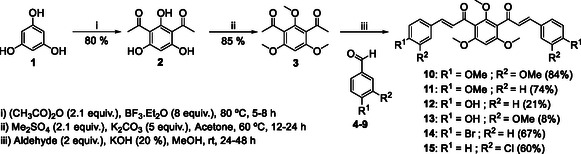
Synthesis of *bis*‐chalcones **10‐15**.

**SCHEME 2:**
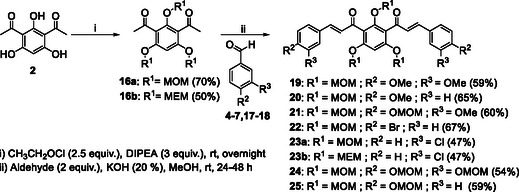
Synthesis of *bis*‐chalcones **19‐25**.

**SCHEME 3:**
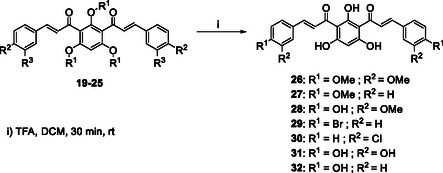
Synthesis of deprotected *bis*‐chalcones **26‐32**.

The synthesis of *bis*‐chalcones was organized into different groups according to the molecule's central moiety (**2**, Scheme 1). The three central hydroxy groups were protected either with methyl groups (**10‐15**, Scheme 1), methoxymethyl and 2‐methoxyethoxymethyl groups (**19‐25**, Scheme 2), unprotected (**26‐32**, Scheme 2) or with combinations of the above (**40‐44**, Scheme 3). The starting material **2** was obtained in good yield (80%) by Friedel‐Crafts acylation of phloroglucinol **1** with acetic anhydride, using BF_3_.Et_2_O as acid catalyst and solvent (Scheme 1). Then, the general approach for the synthesis of the different groups of compounds consisted of three key steps. The first one was the protection of the hydroxy groups of the starting material. This protection step was adopted as an alternative to a direct approach, which could be challenging. This step was followed by the Claisen–Schmidt condensation of the appropriate diketone **3** or **16 a, b** with different benzaldehydes. The deprotection of the hydroxy groups was the last step.

*Bis*‐chalcones **10‐15** were synthesized via Claisen‐Schmidt condensation of product **3** with benzaldehydes **4‐9**. Reactions were performed in methanol at room temperature using potassium hydroxide as a catalyst. Yields ranged from 60% to 84%, except when the benzaldehyde contained free hydroxy groups, in which case yields ranged from 8% to 21% [20]. This discrepancy is related to the additional hydroxy groups (enolates in solution) being potential nucleophiles that compete with the enolate for the condensation reaction, lowering the overall yield significantly.

This issue was later addressed through the protection of the hydroxy groups of the benzaldehyde as well. Compounds **11**, **14,** and **15** were synthesized previously by S. Burmaoglu et al. [21] using the same synthetic protocol and achieving similar yields.

**FIGURE 1:**
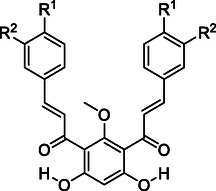
Spatial arrangement of monoprotected *bis*‐chalcones.

Deprotection of all hydroxy groups of the central moiety with BBr_3_ was attempted, but it was unsuccessful despite the prolonged reaction times tested (up to a week). The resulting product always retained one methoxy group (Figure 1). This reactivity could be explained by the structure of *bis*‐chalcones **10‐15** and their spatial arrangement in solution, as evidenced by NMR spectroscopic analysis.

The ^1^H NMR spectra of *bis*‐chalcones **10–15** shared common signals, specifically two signals corresponding to the resonances of the symmetric H‐α and H‐β protons of their two unsaturated carbonyl systems, and the signal due to the resonance of H‐5 from the central A ring. All six compounds showed doublet signals for the H‐α at δ_H_ = 7.35–7.44 ppm and H‐β protons at δ_H_ = 6.87–7.09 ppm, both with *J*_Hα, Hβ_ = 16.1–16.2 Hz, the characteristic coupling constant for double bonds with trans/*E* configuration. The stereochemistry of the *bis*‐chalcones was confirmed by NOESY analysis. The NOESY spectrum of **11**, the *bis*‐chalcone derivative with a methoxy group at 4‐position of the B ring, was obtained, and it revealed the absence of any interactions between the protons of the methoxy groups at positions 4 and 6 of the central moiety and the α/β protons. This result suggests that the molecule does not adopt the linear conformation presented in Scheme 1.

The spatial arrangement of the *bis*‐chalcones suggested in Figure 1 can explain why partial deprotection of the methoxy groups yielded a highly stabilized compound, with two intramolecular hydrogen bonds between the carbonyl groups and the hydroxy groups. The last methoxy deprotection was unfavored since the resulting product would not be more stable, and the conditions were not strong enough. To optimize space and organization, *bis*‐chalcones in the following schemes will be shown linearly, despite a “V” shape being a more appropriate representation.

Because complete deprotection of the methoxy groups was unsuccessful, alternative protecting groups with analogous properties, namely methoxymethyl (MOM) or methoxyethoxymethyl (MEM), were tested. MOM or MEM groups, being slightly bigger, led to greater steric hindrance, but compared to methoxy groups, they are easier to introduce and remove under mild conditions. The groups were introduced in good yields using chloromethyl methyl ether or 2‐methoxyethoxymethyl chloride in basic medium using *N*,*N*‐diisopropylethylamine (DIPEA) at room temperature (Scheme 2). Analysis of the NMR spectra confirmed the presence of the three MOM/MEM groups. The MOM groups were composed of two symmetric CH_2_ groups whose protons’ resonance corresponds to a singlet at δ_H_ = 5.26 ppm (four protons) and another singlet at δ_H_ = 4.88 ppm (two protons) corresponding to the remaining methylene group. The terminal methyl groups showed a similar pattern to the above, corresponding to two singlets at δ_H_ = 3.40 ppm (6H) and δ_H_ = 3.24 ppm (3H). The MEM groups were identified by similar singlets at δ_H_ = 5.27 ppm corresponding to the resonance of the two symmetric CH_2_ groups and δ_H_ = 5.01 ppm from the CH_2_ of the remaining group at the 2‐position. The terminal methyl groups showed similar behavior to the MOM ones. However, additional multiplet signals were detected at δ_H_ = 3.79 ppm (four protons from two symmetric CH_2_), δ_H_ = 3.73 ppm (two protons from the CH_2_ of the MEM group on position 2) and δ_H_ = 3.55 ppm (the remaining six protons). The protected *bis*‐acetophenone **16** underwent Claisen–Schmidt condensation with benzaldehydes **4–7**, **17–18** giving compounds **19–25** (47%–77% yield, Scheme 2).

Deprotection at room temperature with trifluoroacetic acid (TFA) in dichloromethane successfully removed all MOM/MEM groups in quantitative yield without degradation or isomerization, leading to the polyhydroxylated *bis*‐chalcones **26‐32** (Scheme 3). Once the TFA was removed from the mixture by evaporation, no additional purification was needed. ^1^H NMR spectra confirmed the complete deprotection because the signals for the MOM/MEM groups disappeared, and two highly unshielded signals appeared at δ_H_ = 13.42 ppm and δ_H_ = 17.05 ppm, the first singlet corresponding to the two symmetric hydroxy groups and the second corresponding to the remaining OH. The high chemical shifts for these protons can be explained by the intramolecular hydrogen bonds established between the carbonyl groups and the protons of the hydroxy groups.

### 2.2 COX‐1/2 Inhibitory Activity

The above‐mentioned *bis*‐chalcones were tested for their capacity to inhibit PGs production through the inhibition of human COX‐2 and COX‐1 catalysis (isolated forms). Both enzymes share approximately 60% of the same amino acids, of which 84% have similar properties, but have different distributions and functions in the human body, as explained previously [4]. However, they both have similarities in the upper active site (90% homology), which makes it difficult to achieve effective selectivity [4]. Among several amino acid differences, one of particular interest is the shift from an isoleucine at position 523 in COX‐1 to a valine in COX‐2. Compared to isoleucine, valine lacks a methyl group, which provides additional space for the COX‐2 pocket, 17% bigger [5]. Some of the remaining key differences include the replacement of l‐phenylalanine 503, isoleucine 434 and histidine 513 in COX‐1 with leucine 503, valine 434 and arginine 513 in COX‐2 [5]. This substitution increases the flexibility of the pocket walls, making it easier to accommodate larger molecules. Our recently obtained *bis*‐chalcones were tested based on this premise. While regular chalcones are usually potent, they are not usually specific or selective, so we aimed to achieve higher selectivity with these new, larger compounds. As explained in the previous section, these molecules can also adopt a V‐shape, like one of the positive controls used in the following assay, Celecoxib. This resemblance further suggests their potential for good activity.

The COX‐1/2 inhibitory potential of *bis*‐chalcones was assessed with an in vitro fluorometric assay, based on the protocol outlined by Sigma‐Aldrich in their COX‐2 Inhibitor Screening Kit. Table 1 shows the results obtained for the tested *bis*‐chalcones categorized into four different groups, according to the display of the hydroxy groups of the central A‐ring: the methyl protected (**10‐15**, Scheme 1), methoxymethyl and methoxyethoxymethyl protected (**19‐25**, Scheme 2), or unprotected (**26‐32**, Scheme 3).

**TABLE 1:** COX inhibition results and selectivity index for all the tested *bis*‐chalcones; the most active ones are highlighted in bold.

**IC**_**50**_ **,** **µM or Inhibition, %, mean ± SEM**						
***Bis*** **‐chalcones**		R^1^	R^2^	COX‐1	COX‐2	Selectivity index (SI)
**10**	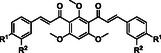	OMe	OMe	<30% ^100 μM^	<30% ^100 μM^	—
**11**		OMe	H	<30% ^100 μM^	<30% ^100 μM^	—
**12**		OH	H	50% ± 9% ^100 μM^ *	47 ± 7% ^100 μM^ *	0.9
**13**		OH	OMe	24 ± 3 µM ^c^	5.5 ± 0.7 µM ^d^	4.4
**14**		Br	H	<30% ^100 μM^	<30% ^100 μM^	—
**15**		H	Cl	<30% ^100 μM^	<30% ^100 μM^	—
**19**	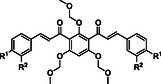	OMe	OMe	<30% ^100 μM^	<30% ^100 μM^	—
**20**		OMe	H	<30% ^100 μM^	<30% ^100 μM^	—
**21**		OMOM	OMe	<30% ^100 μM^	<30% ^100 μM^	—
**22**		Br	H	<30% ^100 μM^	<30% ^100 μM^	—
**24**		OMOM	OMOM	<30% ^100 μM^	<30% ^100 μM^	—
**25**		OMOM	H	<30% ^100 μM^	<30% ^100 μM^	—
**26**	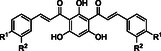	OMe	OMe	51% ± 6% ^100 μM^	40 ± 5% ^100 μM^	0.8
**27**		OMe	H	48 ± 2 µM ^a^	44 ± 3 µM ^a^	1.2
**28**		OH	OMe	37 ± 4 µM ^b^	14.6 ± 0.5 µM ^b^	2.6
**29**		Br	H	51% ± 3% ^100 μM^	58 ± 3 µM ^100 μM^	1.1
**30**		**H**	**Cl**	**10.6 ± 0.6 µM** ^d^	8.6 ± 0.7 µM ^c^	1.2
**31**		**OH**	**OH**	17.0 ± 0.6 µM^c,d^	**1.5 ± 0.3 µM** ^e^	**11.3**
Positive control **Celecoxib**	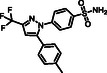			50 ± 1^a^	0.20 ± 0.04 ^f^	251.2
Positive Control **SC‐560**	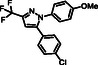			0.0114 ± 0.0007 ^e^	4.0 ± 0.2 ^d^	0.003

*Inhibition (mean, % ± SEM) at the highest tested concentration (in superscript) at the assay conditions. The IC_50_ with different lowercase superscript letters are significantly different from each other (*p* < 0.05). Statistical analysis was only applied within the same enzyme.

**FIGURE 2:**
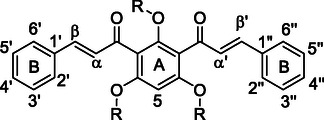
Numeration of the *bis*‐chalcone scaffold.

All four groups of compounds show similar patterns of substitutions on the symmetric B‐rings (Figure 2), including methoxy groups at positions 3′, 3″ and 4′, 4″, a bromine substituent at positions 4′ and 4″, a chlorine at positions 3′ and 3″, and hydroxy groups at positions 3′, 3″ and/or 4′, 4″. In one case, a methoxymethyl group was present at positions 3′, 3″ and 4′, 4″. These varied substitution patterns helped elucidate the structure‐activity relationship.

In total, 18 novel *bis*‐chalcones were tested for the first time as inhibitors of two different enzyme isoforms, COX‐1 and COX‐2, and their activities were compared through a selectivity index (SI) to assess whether there was selectivity towards COX‐2, the intended target. Celecoxib was the positive control used as a selective inhibitor of COX‐2, and SC‐560 was the positive control used as a selective inhibitor of COX‐1.

Out of compounds **10–15** in the initial group, two were found to be active: *bis*‐chalcone **12** containing a *para*‐hydroxy group and *bis*‐chalcone **13** with a *para*‐hydroxy group and a *meta*‐methoxychalcone. *Bis*‐chalcone **12** showed a nonselective inhibitory effect, with a maximum inhibition of approximately 50% at 100 μM, for both enzymes. *Bis‐*chalcone **13** showed a potent and selective inhibition of COX‐2, with an IC_50_ value of 5.5 ± 0.7 μM for this enzyme and an IC_50_ value of 24 ± 3 μM for COX‐1, corresponding to an SI of 4.4. These results suggest that the presence of the hydroxy group at positions 4′ and 4″, present in both *bis*‐chalcones **12** and **13**, is critical for the activity. All other compounds without hydroxy groups, with either methoxy groups or halogens, were inactive. This behavior is consistent with chalcone derivatives reported in the literature [22, 23].

Nonetheless, in the case of *bis*‐chalcone **13,** the introduction of the methoxy group at positions 3′ and 3″ significantly enhanced its activity and selectivity compared to *bis*‐chalcone **12**. This result demonstrates that interactions at this position are critical for a high activity profile. It is important to note that this methoxy group at positions 3′ and 3″ was also present in *bis*‐chalcone **10**, but by itself, it did not lead to any relevant effect, requiring the synergistic effect of the hydroxy group at the *para‐*position.

For the following group **19–25**, with methoxymethyl groups on the central A‐ring, no relevant activity was detected for any of the derivatives at the highest concentration tested. The lack of free hydroxy groups on the B‐rings in all compounds supports the previous finding that these groups are critical for activity.

In the third group of compounds, **26–31**, all have three free hydroxy groups in addition to the previously used substitutions on B‐rings (*bis*‐chalcones **10–15**). With just this modification alone, all *bis*‐chalcones became active, showing that there is a clear interaction between these substituents and the enzyme. Activity was now observed across substitution patterns that previously were inactive, including *meta‐OMe*, *para*‐OMe, *para‐*OH, *para‐*Br, and *meta‐*Cl. However, there were noticeable differences between them. Derivatives **26,** containing two methoxy groups on the B‐rings, and the brominated *bis*‐chalcone **29** showed weak activity, achieving 40%–50% inhibition at 100 μM, and no selectivity toward any of the enzymes. Derivative **27**, which contains only one methoxy group at the *para‐*position, is slightly more active (IC_50_ value of 44 ± 3 μM for COX‐2), indicating that two methoxy groups are detrimental for the interactions established. Therefore, these activities seem to result from a nonspecific interaction of these hydroxy groups with both COX isoforms. These findings also corroborate earlier reports showing that chalcones with methoxy and bromine groups fail to inhibit either COX‐2 or COX‐1 [24].

*Bis*‐chalcone **30**, with a chlorine substituent at *meta‐* position was the most active compound for COX‐1, with an IC_50_ of 10.6 ± 0.6 µM, and an IC_50_ of 8.6 ± 0.7 µM for COX‐2. These results yielded an SI of 1.2. Among the most active compounds tested, *bis*‐chalcone **30** was the least selective. The importance of the *meta* position is shown again, and it also suggests that this compound interacts in a similar way with both enzymes. This role of the chlorine substituent in the *meta‐*position of chalcone‐based inhibitors, leading to higher COX‐2 inhibitory activity, has already been demonstrated before in the literature [5, 23]. Also, in the few examples of active *bis*‐chalcone analogs as COX‐2 inhibitors known in the literature, the chlorine substituent was present in the structure of the most active compound, albeit in the *para‐*position of the side rings [18, 19].

The two other most active *bis*‐chalcones of this group were **28**, with a hydroxy group at positions 4′ and 4″ and a methoxy group at positions 3′ and 3″, and **31**, with two hydroxy groups at positions 3′, 4′ and 3″, 4″. Compound **28** (IC_50_ of 14.6 ± 0.5 µM for COX‐2) can be directly compared to compound **13**, as it shares the same substitution pattern on B‐rings. The importance of the substitution at the *meta*‐position is again shown. It can only be assumed that compounds **13** and **28** did not bind to the same site on the enzymes.

The most potent *bis*‐chalcone tested was **31** (IC_50_ of 1.5 ± 0.3 µM for COX‐2), with hydroxy groups at positions 3′, 4′ and 3″, 4″. It was the compound whose activity most closely approached that of the positive control celecoxib (IC_50_ of 0.2 ± 0.04 µM for COX‐2). COX‐2 selectivity was highest for this compound (SI = 11.3), suggesting that this moiety likely interacts with specific sites on COX‐2 that are not present in COX‐1, unlike *bis*‐chalcone **30**, which interacted similarly with both enzymes. It may not be as selective as celecoxib (SI = 251), but this could be considered a positive feature, acting as a balanced selective inhibitor of COX‐2. Disproportionate selective inhibition of COX‐2 is a cause of one of the most common adverse side effects of celecoxib, cardiotoxicity [6, 25, 26]. A lower selectivity index may help reduce this side effect [27].

A comparison of compound **31** with **28** also illustrates an improvement in activity by substituting the methoxy group at the *meta‐*position with a hydroxy group, which allows for further hydrogen bonding interactions. This catechol moiety has been recognized in the literature to play a role in the COX‐2 inhibition activity of other derivatives [22, 25, 26, 27, 28]. Most authors highlight the importance of this moiety in the interaction of the inhibitor with the hydrophilic pocket of the enzyme. However, to the best of our knowledge, this is the first time *bis*‐chalcone derivatives containing this catechol moiety were tested as inhibitors of the human COX‐2 enzyme.

**FIGURE 3:**
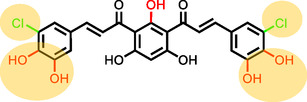
Potential substitution pattern associated with the highest COX‐2 inhibitory activity.

Analysis of all tested *bis‐*chalcones allowed us to conclude which substitution patterns best inhibit COX‐2 (Figure 3). Within the derivatives with free hydroxy groups at the central ring, the two best substitutions for the B rings were a catechol moiety and a chlorine at the *meta‐*position of the side rings. Methoxy groups and bromine groups were ineffective unless combined with already present hydroxy groups.

### 2.3 Type of Enzyme Inhibition

Following the results obtained, two compounds, **30** and **31**, were selected as the most potent *bis*‐chalcones for COX‐1 and COX‐2, respectively. Their inhibition types were calculated through visual evaluation of Lineweaver–Burk plots and statistical analysis of experimental data with nonlinear regression. The Solver supplement of Microsoft Office Excel was employed for this purpose [29].

**FIGURE 4:**
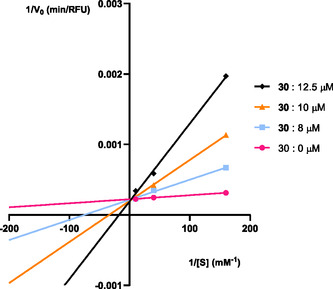
Lineweaver–Burk plot of COX‐1 inhibition by compound **30**. The mean values are represented by the different symbols. Each line was obtained with data from at least three independent experiments. The lines were obtained according to the best fitted model of competitive inhibition. The parameters obtained for this model were V_max_ = 4582 ± 52 μmol/min, K_m_ = 2.7 ± 0.2 μM, K_ic_ = 1.24 ± 0.07 μM,.

**FIGURE 5:**
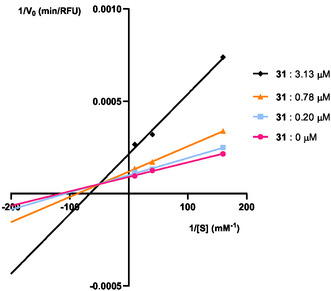
Lineweaver–Burk plot of COX‐2 inhibition by compound **31**. The mean values are represented by the different symbols. Each line was obtained with data from at least three independent experiments. The lines were obtained according to the best fitted model of mixed inhibition. The parameters obtained for this model were V_max_ = 11 109 ± 109 μmol/min, K_m_ = 8.9 ± 0.2 μM, K_ic_ = 1.34 ± 0.08 μM, K_iu_ = 2.02± 0.07 μM.

At least three experiments were conducted for each compound, considering three inhibitor concentrations in the range of 8–15 µM for compound **30**, from 0.2 to 3.2 µM for compound **31**, and three substrate concentrations in the range of 6.25–100 µM. The experimental data were obtained from the slope of the kinetic reaction during the first 2 minutes after substrate addition, corresponding to the initial velocity of the linear steady state phase. This data was plotted in Lineweaver–Burk plots (Figures 4 and 5), using the linearized form of the Michaelis–Menten equation described in the experimental section. Visual inspection of the intersection points among the lines corresponding to each inhibitor's concentration provides information about the type of inhibition kinetics associated with that particular inhibitor. Intersection at the *y*‐axis is indicative of a competitive inhibition profile, intersection at the *x*‐axis corresponds to non‐competitive inhibition, and intersection within the first quadrant reflects a mixed‐type inhibition pattern. This data was also sequentially fitted to various hyperbolic equation models accounting for the different inhibition models (without inhibition, competitive, noncompetitive, uncompetitive, or mixed inhibition), by nonlinear least squares regression. From this fitting, the corresponding kinetic parameters and the sum of the square errors’ values for each model were obtained and compared to determine the best model. The extra sum‐of‐squares F test and the Akaike information criterion (AIC) test were further considered for model validation.

With the experimental data obtained, *bis*‐chalcone **30** exhibited a competitive inhibition mechanism of COX‐1, visible by the Lineweaver‐Burk plot in Figure 4, with the related kinetic constant values (V_max_, K_m_, K_ic_ and K_iu_) presented in Table 2. *Bis*‐chalcone **31** was recognized as a mixed‐type inhibitor of COX‐2 (Figure 5), with the corresponding kinetic parameters also presented in Table 2.

**TABLE 2:** Type of inhibition (using Solver supplement) of the tested compounds **30** and **31** against COX‐1 and COX‐2 activities and respective kinetic parameters values: V_max_, K_m_, K_ic_ and K_iu_ (mean ± SEM).

Compound	Type of inhibition	Vmax, μmol/min	**K**_**m**_ **,** **μM**	**K**_**ic**_ **,** **μM** ^**−1**^	**K**_**iu**_ **,** **μM** ^**−1**^
**COX‐1**					
**30**	Competitive	4582 ± 52	2.7 ± 0.2	1.24 ± 0.07	—
**COX‐2**					
**31**	Mixed	11 109 ± 109	8.9 ± 0.2	1.34 ± 0.08	2.02 ± 0.07

Compound **30** exhibited a competitive inhibition mechanism, and this mechanism was deemed the most appropriate model since it yielded the smallest sum of squared residuals after performing iterative nonlinear regression using the Solver supplement.

The corresponding Lineweaver–Burk plot (Figure 4), generated from the same experimental data, confirmed the expected behavior of a competitive inhibitor. As illustrated in Figure 4, when the concentration of **30** increased, the V_max_ remained constant (all concentrations share the same value when 1/[S] = 0, the definition of V_max_), but the K_m_ (when 1/[V_0_] = 0) value increased, indicating a lower affinity of *bis*‐chalcone **30** for the enzyme. Since compound **30** is a competitive inhibitor, it competes directly with arachidonic acid for the same active site on COX‐1. Given that *bis*‐chalcone **30** is not a specific inhibitor, this *bis*‐chalcone interacts with the common active site cavity of both COX‐1 and COX‐2 isoforms. This result is corroborated by examples in the literature, where the chlorinated inhibitors also bind to the active site of COX‐1 [23, 30].

Compound **31**, which was confirmed as the most potent compound for COX‐2, has been characterized as a mixed inhibitor. As illustrated in Figure 5, with the increase in concentration, both V_max_ and K_m_ increase, according to the expected behavior of a mixed inhibitor. A distinctive feature of this mechanism is the binding to the free enzyme or the enzyme‐substrate complex. This behavior can be attributed to the presence of the catechol moiety, which has the potential to bind to multiple spots on the enzyme due to its high hydrogen bonding capacity. Consequently, the interaction of this compound is not limited to the active site.

### 2.4 Molecular Docking Studies

Compounds **30** and **31** were identified as the most potent COX‐1 and COX‐2 inhibitors in this study. Therefore, molecular docking calculations were performed to rationalize the molecular mechanisms underlying their inhibitory activity. The objective was to propose a structural rationale hypothesis to explain the potency of these *bis*‐chalcones, in particular, for the markedly higher selectivity of compound **31** for COX‐2 over COX‐1.

The docking calculations protocol was optimized for docking compounds **30** and **31**. This essential preparatory stage involved extensive self‐ and cross‐docking calculations using 20 COX‐1 and 6 COX‐2 crystal structures obtained from the Protein Data Bank (PDB). Structures were selected based on two key criteria: the presence of a co‐crystallized ligand and an X‐ray resolution of 3 Å or better. Interestingly, although six X‐ray structures are available for human COX‐2, the PDB contains only one human COX‐1 structure, an apo form lacking any bound ligand (PDB ID: 6Y3C). Consequently, 19 holo structures from sheep COX‐1 that met the selection criteria were incorporated into the optimization of the docking protocol.

Upon completion of the self‐ and cross‐docking calculations, we analyzed the results and excluded all structures where the majority of cross‐docking calculations were unsuccessful in the software packages tested. This analysis led to the selection of 14 COX‐1 structures for final consideration and validation of the docking protocol (Supplementary Figure S77). All six COX‐2 structures were deemed suitable for validating the docking protocol (Supplementary Figure S78).

Upon evaluating the performance of various docking software through self‐ and cross‐docking calculations that included all selected proteins and their respective ligands, for COX‐2, the structure 5IKT proved most effective in accurately reproducing not only its own crystallographic pose but also the poses of other X‐ray ligands found in the PDB. For COX‐1, although PDB ID 6Y3C did not show the best cross‐docking performance, as illustrated in Supplementary Figure S1 (showing that fewer structures were reproduced with an RMSD < 2 Å), it still enabled a strong overall performance, with 9 out of 10 ligands displaying RMSD values below 2 Å. This, and the fact that it is the only human structure, prompted its selection for our docking calculations (for further details, please refer to the Methods section). The performance of the human structure demonstrated its capability to accurately position the X‐ray ligands from the sheep structures. Since all assays in this study were conducted using human COX‐1, this structure was selected for the docking calculations. Glide was identified as the most effective software in both scenarios, demonstrating superior accuracy and reliability.

**FIGURE 6:**
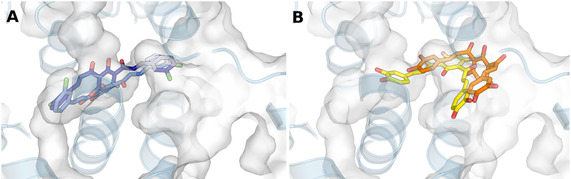
Predicted binding poses for compounds **30** and **31** within the binding sites of COX‐1 (PDB ID: 6Y3C) and COX‐2 (PDB ID: 5IKT). Both proteins are overlaid for comparison, but the surface shown corresponds to COX‐2. (A) Compound **30** top pose within the COX‐1 (dark blue) and within the COX‐2 (light blue) binding sites. (B) Compound **31** top pose within the COX‐1 (orange) and COX‐2 (yellow) binding sites.

After establishing optimal conditions, docking calculations were performed for compounds **30** and **31** against COX‐1 (PDB ID: 6Y3C) and COX‐2 (PDB ID: 5IKT) to elucidate their binding modes and the molecular interactions underlying the selectivity of compound **31** for COX‐2 (SI = 11.3). The binding cavities for COX‐1 and COX‐2 were chosen based on the region known to interact with non‐steroidal anti‐inflammatory drugs (NSAIDs), guided by structural data demonstrating the molecular configurations typically involved in NSAIDs’ interactions with these enzymes (Figure 6). The identified ‘common’ pocket consists of a hydrophobic channel that extends from the membrane‐binding domain (the entrance) to the core of the catalytic domain, adopting an L‐shaped configuration. The substrate binding site, also known as the arachidonate‐binding site, was located in the first half of the channel, spanning from Arg120 to near Tyr385 [6].

Initially, we inspected the binding poses of compounds **30** and **31** within the COX‐1 and COX‐2 binding sites, comparing the regions occupied by these compounds with the X‐ray crystallographic poses of known inhibitors where such data were available (e.g., celecoxib and tolfenamic acid, Figure S79). The docking results indicated that compound **30** occupies the same region of the binding pocket in COX‐1 and COX‐2, with the molecules nearly superimposed (Figure 6A). This could explain the similar inhibitory activity observed, with IC_50_ values of 10.6 and 8.6 µM, respectively.

Conversely, compound **31** adopts a distinct conformation within the COX‐1 and COX‐2 binding sites (Figure 6B). The unique positioning of compound **31**, which is not observed in any of the COX‐2 inhibitors, aligns with the fact that this compound has been determined to be a mixed inhibitor. The predicted pose for this compound does not preclude substrate binding, suggesting that it might act noncompetitively. Therefore, we hypothesize that the distinct binding locations of both compounds in COX‐1 and COX‐2 contribute to their observed selectivity, as evidenced by SI values of 1.2 and 11.3 for compounds **30** and **31**, respectively.

**FIGURE 7:**
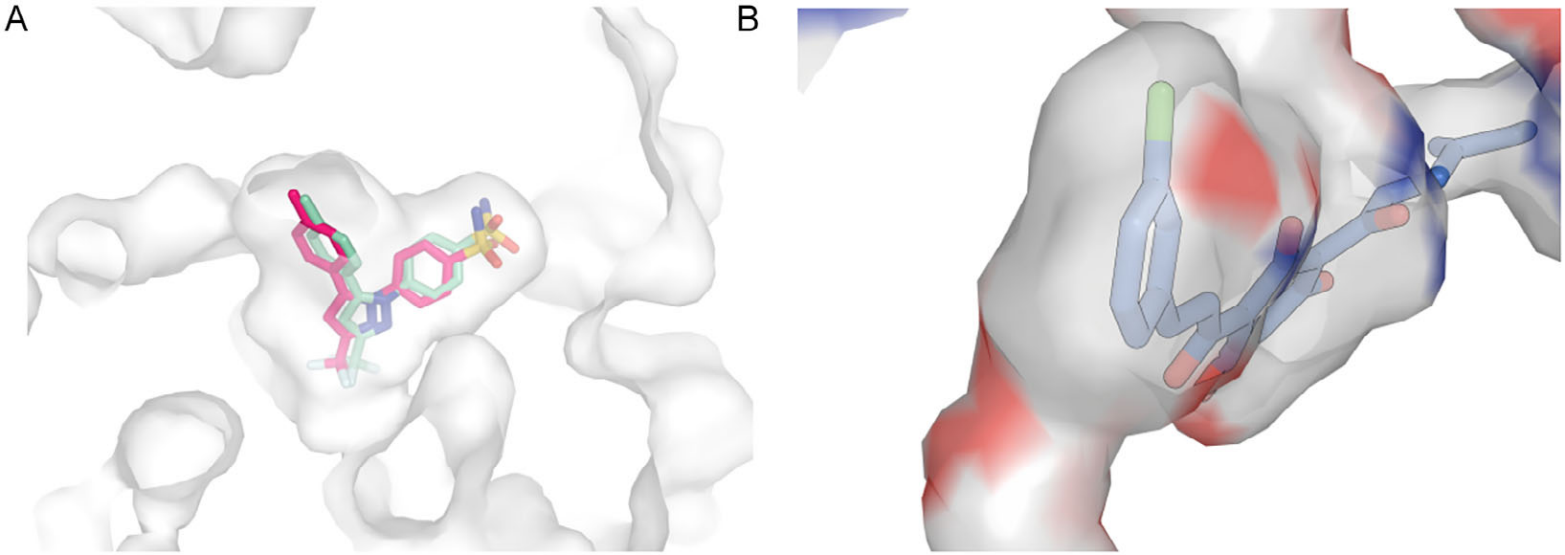
(A) Celecoxib co‐crystallized with COX‐1 (shown in pink) and COX‐2 (shown in sage), highlighting structural similarities**.** (B) The back of the initial segment of the COX‐1/COX‐2 binding sites is mostly hydrophobic, with gray regions indicating the presence of carbons and red regions indicating oxygen atoms.

**FIGURE 8:**
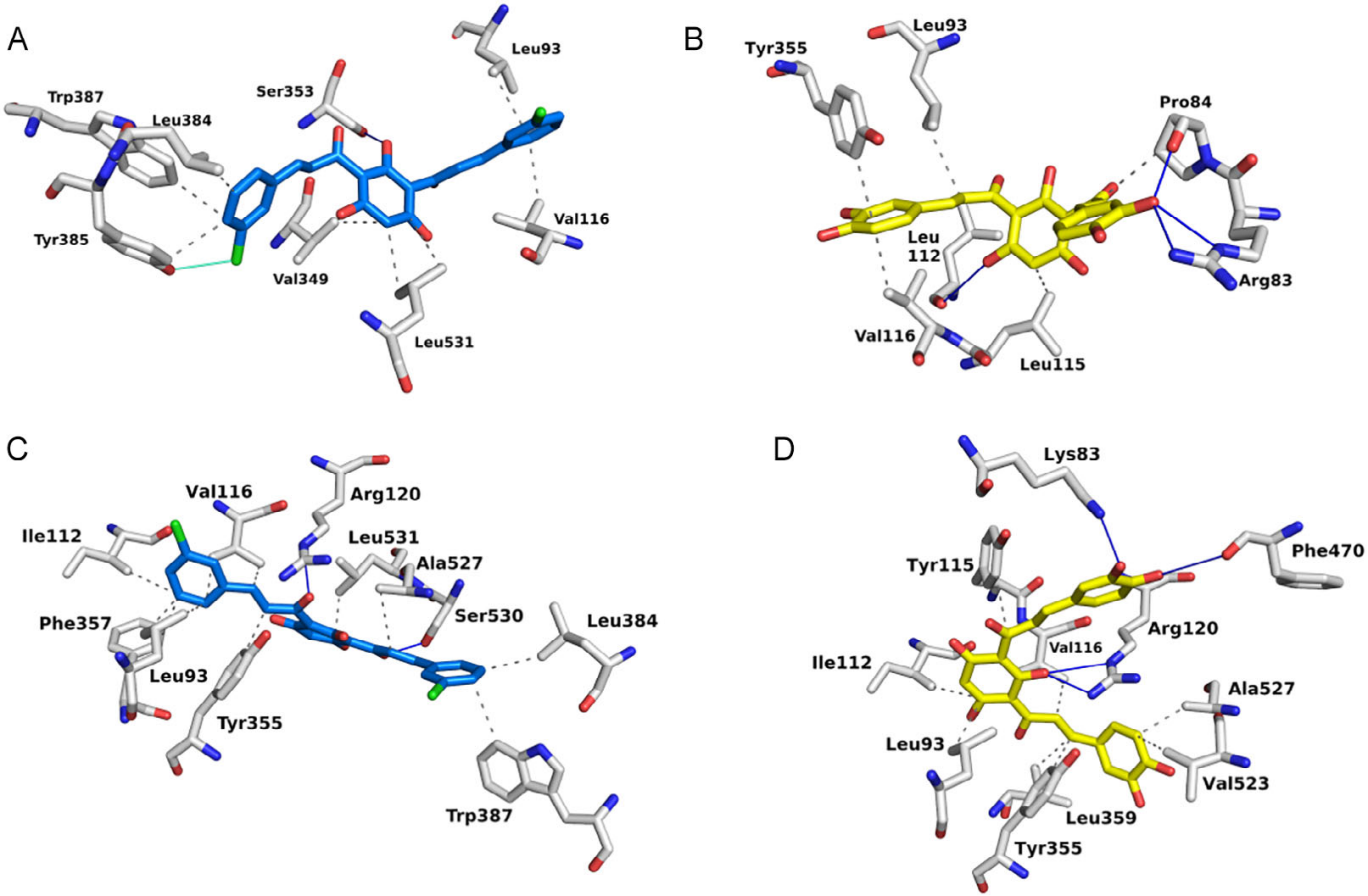
Binding modes within the COX‐1 and COX‐2 binding pockets. (A) Interactions between compound **30** and COX‐1. (B) Interactions between compound **31** and COX‐1. (C) Interactions between compound **30** and COX‐2. (D) Interactions between compound **31** and COX‐2. Dotted lines represent hydrophobic interactions, blue lines indicate hydrogen bonds, and green lines denote halogen bonds. (C) and (D) are turned so that the right‐hand side of the image faces the back of the pocket.

However, it is worth noting that celecoxib's X‐ray poses in COX‐1 and COX‐2 are almost identical (as shown in Figure 7). This led us to reformulate our hypothesis: the placement itself may not be the primary factor behind selectivity. Instead, it may indirectly indicate differences in interactions that result from the unique placement of compound **31.** Celecoxib was selected for this analysis as it is the only ligand that has been crystallized with COX‐1 and COX‐2. For compound **30**, the presence of a chlorine substituent facilitates a halogen bond and a hydrophobic interaction with Tyr385 (Figure 8), in contrast to the hydroxy substituent at this position in compound **31**. The hydroxy group may prevent this interaction, as no electrophilic atom is available at either end of the ligand to form such interactions. These interactions with Tyr385 are potentially crucial for the observed loss of selectivity of compound **30**, given that this residue participates in an intramolecular electron transfer essential for the conversion of arachidonic acid into an arachidonyl radical in COX‐1 [31].

Additionally, we hypothesize that there is no additional space for a *para‐*substituent in compound **30** near the pocket bend, as seen in Figure 6A. This spatial limitation might prevent compound **31** from penetrating deeply into the pocket, especially considering that the region where the binding site bends is mostly hydrophobic and would not easily accommodate a polar *para‐* substituent such as ‐OH (illustrated in Figure 7B). The presence of polar regions above and below the ligand in Figure 7 suggests that polar substitutions might be angled towards these two places (*ortho‐* or *para‐*). Another hypothesis is that compound **31**, which possesses both *meta*‐ and *para*‐hydroxy groups, may be too bulky to fit through the narrow entrance of the binding site.

The main difference between the two binding pockets is tied to three amino acid residue changes, resulting in COX‐2 possessing a binding channel that is approximately 20% larger and more accessible channel than that of COX‐1 [6]. As stated before, the key substitution from isoleucine to valine at position 532 that leads to a structural modification that provides access to a side pocket was reported as a feature in COX‐2 selectivity [6] but is not an absolute requirement (as evident by the fact that, all X‐ray ligands in COX‐2, except for celecoxib, occupy the placement shown in Figure S78). Although neither compound **30** nor compound **31** enters this side pocket, unlike celecoxib, compound **31** is positioned near its entrance (Figure 6B, highlighted in yellow), while compound **30** is situated farther away (shown in Figure 6A, in light blue).

**FIGURE 9:**
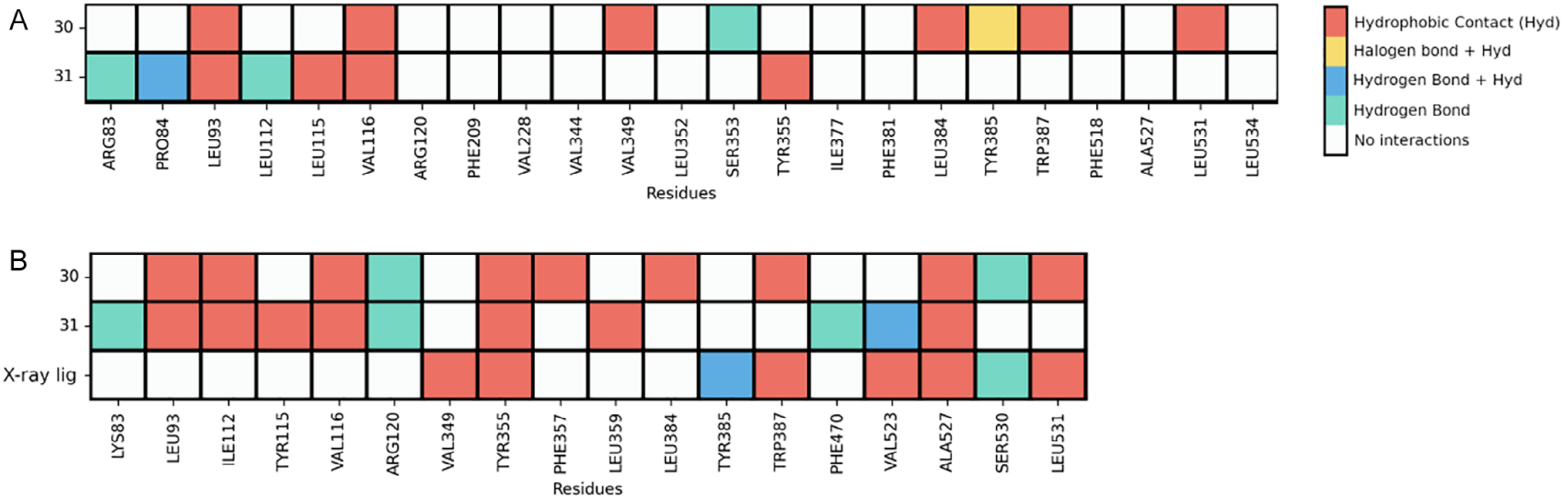
(A) Protein–Ligand interactions (PLIs) observed for the top docking poses for compounds **30** and **31** in COX‐1 (no X‐ray ligand existed for this structure). (B) PLIs for compounds **30**, **31,** and tolfenamic acid (COX‐2's X‐ray ligand from PDB ID: 5IKT) interacting with COX‐2.

Significant differences were observed in the specific molecular interactions established between compounds **30** and **31** with COX‐1 and COX‐2 (Figure 9), which illustrates the interaction profiles of these compounds compared with existing X‐ray ligands in the utilized structures (i.e., tolfenamic acid in COX‐2). Compound **31** is predicted to establish hydrogen bonding with two residues simultaneously in COX‐1 (either Arg83 or Pro84 and Leu112), whereas it forms hydrogen bonds with four residues in COX‐2 (Lys83, Arg120, Phe470, and Val523). Furthermore, it should be noted that the multiple hydrophobic bonds, such as those observed between compound **30** and COX‐2, do not necessarily indicate strong binding, as these are very weak interactions. Therefore, the primary binding drivers are hydrogen bonds and, to a lesser extent, halogen bonds. The pronounced difference in the number of hydrogen bonds formed by the two compounds aligns with the significant difference in biological activity, contributing to the increased COX‐2 selectivity observed for compound **31**. We hypothesize that the chlorine substituent in compound **30** may position this scaffold in a manner that leads to suboptimal binding interactions with COX‐2, explaining the loss of activity for compound **30** against this target. In terms of potency, compounds **30** and **31** exhibit COX‐2 inhibitory activity in the low micromolar range (8.6 and 1.5 µM, respectively), compared to tolfenamic acid (IC_50_ = 640 nM, measured in rabbit cell [32]. This is noteworthy, given that compound **31** shares only three overlapping contacts with tolfenamic acid and compound **30** only five (Figure 9B). Similar to both *bis*‐chalcones, tolfenamic acid relies heavily on hydrophobic interactions to stabilize its position within the COX‐2 pocket.

The reduced potency of compound **30** relative to compound **31** and tolfenamic acid may be associated with its lower hydrogen‐bonding capacity within the COX‐2 binding pocket. In contrast, compound **31** is predicted to form two hydrogen‐bond interactions, which may contribute to its improved binding affinity and inhibitory activity. Additionally, interactions with Tyr385, a key residue in COX‐2 catalysis, may further modulate both selectivity and activity. Nevertheless, other factors, including ligand orientation and hydrophobic interactions, are also likely to play a significant role.

### 2.5 ADME Profile Studies

Alongside the molecular docking studies, the pharmacokinetic properties of compounds **30** and **31** were evaluated to assess their potential bioavailability. Several computational tools are available to predict the absorption, distribution, metabolism, excretion, and toxicity (ADMET) profiles of drug candidates at early stages of drug development, as well as their overall drug‐likeness. In this study, the online software PreADMET was used to predict these properties for the two novel *bis*‐chalcones. The predictions were based on approximately 2,500 molecular descriptors, including constitutional, topological, electrostatic, physicochemical, and geometrical parameters [33].

Drug‐likeness was assessed using Lipinski's rule of five and additional criteria derived from drug‐like compound databases such as the CMC, World Drug Index (WDI), and MDDR [33]. ADME properties were evaluated using multiple parameters, including predicted cytochrome P450 interactions, Caco‐2 and MDCK cell permeability models, blood–brain barrier penetration, human intestinal absorption, plasma protein binding, and skin permeability [33]. All data acquired can be consulted in the supporting information on Tables S1 and S2.

Compound **30** was classified as drug‐like according to Lipinski's rule of five (Table S1), despite exhibiting one violation related to its AlogP98 value (an atom‐based method for LogP calculation). It was classified as drug‐like according to the MDDR criteria, with no reported violations. However, it failed in all other categories. Compound **30** did not qualify as drug‐like according to the CMC criteria, presenting a single violation also associated with the AlogP98 value. It also violated the lead‐like rule, with two infractions related to molecular weight and AlogP98 value. Finally, compound **30** fell outside the 90% cutoff for the World Drug Index (WDI), presenting two violations associated with AlogP98 value and molar refractivity (AMolRef).

With respect to ADME properties, compound **30** exhibited a high predicted human intestinal absorption rate (94.9%) and complete plasma protein binding (Table S1). The compound showed moderate Caco‐2 permeability but very low MDCK permeability, suggesting limited transcellular transport. Blood–brain barrier penetration was predicted to be relatively high, likely related to its higher lipophilicity. Compound **30** was predicted to act as an inhibitor of several cytochrome P450 isoforms. Additionally, the compound was predicted to inhibit P‐glycoprotein. Solubility predictions indicated low aqueous solubility in both pure water and buffer, consistent with its high lipophilicity. Skin permeability was predicted to be moderate. Overall, the elevated lipophilicity and poor solubility of compound **30** may limit its bioavailability despite favorable intestinal absorption.

Compound **31** showed superior predicted behavior compared to compound **30**. It was classified as drug‐like according to the CMC, MDDR, Lipinski's rule of five, and WDI criteria, although it violated one parameter in the latter two rules (Table S2). Specifically, the number of hydrogen bond donors (7) exceeded the recommended limit. In addition, compound **31** did not comply with the lead‐like criteria.

Regarding its ADME properties, compound **31** exhibited a moderate predicted human intestinal absorption rate (55.0%) and showed complete plasma protein binding. The compound displayed moderate Caco‐2 permeability and very low MDCK permeability. Blood–brain barrier penetration was predicted to be low, linked to higher hydrophilicity. Compound **31** was predicted to inhibit several cytochrome P450 isoforms, including CYP2C19, CYP2C9, and CYP3A4. In addition, compound **31** was predicted to inhibit P‐glycoprotein. Solubility predictions indicated high solubility in buffer and moderate solubility in pure water, consistent with its lower lipophilicity. Skin permeability was predicted to be low. Overall, the improved solubility and reduced lipophilicity of compound **31**, provided by the additional hydroxy groups, may favor its bioavailability, although its moderate intestinal absorption and extensive plasma protein binding could limit its applicability.

Overall, both compounds exhibited weaknesses commonly found in hit‐like molecules. Both compounds showed complete plasma protein binding, which may be attributed to extensive hydrogen bonding involving the hydroxy groups and/or covalent interactions between plasma protein cysteine residues and the α,β‐unsaturated moiety acting as a Michael acceptor [34]. In addition, both compounds are likely to undergo rapid metabolism by cytochrome P450 isoforms.

Compound **30** displayed high lipophilicity, which may limit its applicability in most biological systems. Compound **31** exhibited more favorable drug‐like properties. However, it still presents some pharmacokinetic liabilities that require further optimization.

Additional strategies will therefore be necessary to improve the pharmacokinetic profiles of both compounds without compromising their biological potency. Rational molecular design will be the primary approach, including the incorporation of bioisosteric modifications to enhance solubility and reduce metabolic susceptibility while preserving the key interactions with the COX enzymes.

## 3 Conclusion

*Bis*‐chalcones are an emerging family of chalcone derivatives that have yet to demonstrate their full potential as anti‐inflammatory agents. In this context, four different groups of novel *bis*‐chalcones were synthesized for the first time, resulting in 22 new compounds that were fully characterized. From this new library of chalcone derivatives, 18 were tested as inhibitors of both COX‐1 and ‐2 isoforms, to establish an accurate structure–activity relationship, supported by inhibition type determination and molecular docking calculations. The most active compound for COX‐2, with the best SI, was **31**, which contains a catechol moiety in the side rings and three hydroxy groups on the central A‐ring, and it was identified as a mixed‐type inhibitor with good selectivity towards COX‐2. Its selectivity was lower than that of celecoxib, as lower selectivity was intentionally sought to achieve a more balanced selectivity profile towards COX‐2. *Bis*‐chalcone **30,** with a chlorine substituent in the *meta*‐ position of B‐rings, was described as the most active for COX‐1 with a competitive type of inhibition and no relevant selectivity towards either COX isoforms. Molecular docking suggests that the chlorine substituent allows compound **30** to penetrate more deeply into the binding pockets of COX‐1 and COX‐2, potentially leading to a loss of selectivity. In contrast, compound **31**, where two hydroxy groups replace a single chlorine, appears too bulky to fit deeply into the binding pockets. Consequently, it assumes an alternative binding pose, partially interacting with the exterior of the binding site. Unlike *bis*‐chalcone **30**, **31** adopts distinct poses in COX‐1 and COX‐2, with each isoform accommodating the compound in slightly different locations within the binding site. This variation may stem from distinct molecular interactions with each isoform, which could be a key factor driving the selectivity of compound **31**. Intriguingly, the docking results support the biochemical evidence that compound **31** acts as a mixed inhibitor, possibly binding in a non‐competitive manner at an allosteric site. These findings suggest that structural modifications of the substitution patterns of *bis*‐chalcones significantly influence their interaction dynamics and selectivity profiles, even more so than the *bis*‐chalcone skeleton. These results underline the importance of detailed molecular characterization in developing selective COX inhibitors.

## 4 Experimental Section

### 4.1 Chemistry

Reagents and solvents were purchased as reagent grade and used without further purification unless otherwise stated. The following reagents were purchased from Sigma‐Aldrich. Chloromethyl methyl ether; Chloromethyl ethyl ether; substituted benzaldehydes (4‐methoxybenzaldehyde, 3,4‐dimethoxybenzaldehyde, 4‐bromobenzaldehyde; 3‐chlorobenzaldehyde, 4‐hydroxybenzaldehyde, 4‐hydroxy‐3‐methoxybenzaldehyde, 3,4‐dihydroxybenzaldehyde). Phloroglucinol was purchased from BDH Laboratory Reagents.

Reactions were controlled by thin layer chromatography (TLC) using silica gel 60 HF254 plates. Column chromatography was performed using flash silica gel (40‐60 µm, 60A). Melting points were determined with a Büchi melting point B‐540 apparatus and are uncorrected. NMR spectra were recorded with 300 or 500 MHz (300 MHz (^1^H), 75 MHz (^13^C), or 500 MHz (^1^H), 125 MHz (^13^C)). Bruker Avance III NMR spectrometers, with tetramethylsilane (TMS) as the internal reference. Unequivocal ^1^H assignments (δ, ppm) and ^13^C assignments (δ, ppm) were made based on the analysis of 2D HSQC, HMBC, and NOESY experiments, if necessary. Peaks positions are given in parts per million (ppm) using the residual nondeuterated solvent as the internal standard. Data are reported as follows: chemical shift (ppm), integrated intensity, multiplicity (indicated as: s, singlet; br s, broad singlet; d, doublet; t, triplet; q, quartet; m, multiplet and combination thereof), coupling constants (*J*) values in Hertz (Hz). Positive‐ion electrospray (ESI^+^) mass spectra were performed using a linear ion trap mass spectrometer LXQ (ThermoFinnigan, San Jose, CA). Data acquisition and analysis were performed using the Xcalibur Data System (version 2.0, ThermoFinnigan, San Jose, CA). High‐mass‐resolving ESI‐MS were conducted in a *Q*‐Exactive hybrid quadrupole Orbitrap mass spectrometer (Thermo Fisher Scientific, Bremen, Germany).

Untested intermediary compounds containing the protection groups MOM or MEM might not have their mass spectra analysis included, due to the instability of these groups under the ionization conditions. Instability of the final compounds prevented further elemental analysis, so purity is ensured by clean NMR spectra.

### 4.2 Synthesis

#### 4.2.1 General Procedure for the Synthesis of 1,1′‐(2,4,6‐Trihydroxy‐1,3‐phenylene)*bis*(ethan‐1‐one) (2)

Acetic anhydride (1.50 mL, 15.86 mmol) was added to a solution of boron trifluoride diethyl etherate (8 mL, 63.43 mmol). After 15 min, phloroglucinol **1** (1.0 g, 7.920 ml) was added slowly in portions. The mixture was stirred at 90°C for 14h under nitrogen, poured into ice, quenched with a solution of potassium acetate (4.1 M, 10 mL), and the product was extracted with ethyl ether (3 × 50 mL). The organic layers were washed with a saturated solution of potassium bicarbonate and water, dried over anhydrous sodium sulfate, and concentrated under reduced pressure. The residue was purified by silica gel column chromatography with hexane/ethyl acetate (4:1) as eluent.

**1,1′‐(2,4,6‐Trihydroxy‐1,3‐Phenylene)*bis*(Ethan‐1‐one)** (**2**) white solid, 80% yield, m.p. 161.6–163.4ºC. ^1^ **H NMR** (300 MHz, Acetone‐*d*_6_) δ 5.94 (s, 1H, H‐5), 2.66 (s, 6H, 1‐COCH_3_, 3‐COCH_3_). ^13^ **C NMR** (75 MHz, Acetone‐*d*_6_) δ 203.8 (1‐COCH_3_, 3‐COCH_3_), 171.9 (C‐2, C‐4, C‐6), 103.8 (C‐1, C‐3), 94.7 (C‐5), 32.0 (1‐COCH_3_, 3‐COCH_3_). **MS** (ESI^+^) m/z (%): 211.0 [M + H]^+^ (60). **HRMS** (ESI^+^) m/z calcd for C_10_H_11_O_5_: 211.0601 [M + H]^+^; found: 211.0600.

#### 4.2.2 General Procedure for the Synthesis of 1,1′‐(2,4,6‐Trimethoxy‐1,3‐Phenylene)*bis*(ethan‐1‐one) (3)

Dimethyl sulfate (0.28 mL, 2.95 mmol) was added to a suspension of **2** (0.20 g, 0.95 mmol) and potassium carbonate (0.66 g, 4.76 mmol) in acetone (30 mL). The mixture was stirred at reflux overnight, quenched with NaOH solution (1M), and the product was extracted with DCM (30 mL). The organic layer was dried over anhydrous sodium sulfate, evaporated to dryness, and the residue was purified by silica gel column chromatography using dichloromethane as eluent to yield compound **3** in 85% yield as a white solid after several hours in the high vacuum pump.

**1,1′‐(2,4,6‐Trimethoxy‐1,3‐phenylene)*bis*(ethan‐1‐one)** (**3**) white solid, 85% yield, m.p. 107.5–108.2ºC. ^1^ **H NMR** (300 MHz, Acetone‐*d*_6_) δ 6.62 (s, 1H, H‐5), 3.92 (s, 6H, 4‐OCH_3_, 6‐OCH_3_), 3.70 (s, 3H, 2‐OCH_3_), 2.41 (s, 6H, 1‐COCH_3,_ 3‐COCH_3_). ^13^ **C NMR** (75 MHz, Acetone‐*d*_6_) δ 199.4 (1‐COCH_3_, 3‐COCH_3_), 158.7 (C‐4, C‐6), 155.0 (C‐2), 118.6 (C‐1, C‐3), 91.8 (C‐5), 63.6 (2‐OCH_3_), 55.7 (4‐OCH_3_, 6‐OCH_3_), 31.6 (1‐COCH_3_, 3‐COCH_3_). **MS** (ESI^+^) m/z (%): 253.1 [M + H] ^+^ (100).

#### 4.2.3 General Procedure for Synthesis of *Bis*‐Chalcones 10–15

Compound **3** (0.20 g, 0.79 mmol) was dissolved in methanol (40 mL) and NaOH (4.0 g) was added, and, after stirring for 30 min at room temperature, the corresponding benzaldehyde (2.1 equiv.) was added to the mixture. (**10**: 3,4‐dimethoxybenzaldehyde; **11**: 4‐methoxybenzaldehyde; **12**: 4‐hydroxybenzaldehyde; **13** 4‐hydroxy‐3‐methoxybenzaldehyde; **14** 4‐bromobenzaldehyde; **15**: 3‐chlorobenzaldehyde). The mixture was stirred at room temperature for 24 – 48 h, then it was poured into ice and quenched with diluted hydrochloric acid to adjust the pH to 4. The product was extracted with DCM (3 × 20 mL). The organic layers were dried over anhydrous sodium sulfate, evaporated to dryness, and the residue was purified by silica gel column chromatography using a hexane/ethyl acetate 3:2 mixture as eluent.

**(*2E*,*2′E*)−1,1′‐(2,4,6‐Trimethoxy‐1,3‐phenylene)*bis*[3‐(3,4‐dimethoxyphenyl)prop‐2‐en‐1‐one]** (**10**), yellow solid, 84% yield, m.p. 176.4–177.0ºC. ^1^ **H NMR** (300 MHz, Acetone‐*d*_6_) δ 7.37 (d, *J* = 16.1 Hz, 2H, H‐β, H‐β’), 7.35 (d, *J* = 2.1 Hz, 2H, H‐2′, H‐2″), 7.23 (dd, *J* = 8.4, 2.1 Hz, 2H, H‐6′, H‐6″), 7.00 (d, *J* = 8.4 Hz, 2H, H‐5′, H‐5″), 6.93 (d, *J* = 16.1 Hz, 2H, H‐α, H‐α’), 6.70 (s, 1H, H‐5), 3.89 (s, 6H, 6‐OCH_3_, 4‐OCH_3_), 3.89 (s, 6H, 4′‐OCH_3_, 4″‐OCH_3_), 3.87 (s, 6H, 3′‐OCH_3_, 3″‐OCH_3_), 3.65 (s, 3H, 2‐OCH_3_). ^13^ **C NMR** (75 MHz, Acetone‐*d*_6_) δ 192.5 (1‐COCH‐, 3‐COCH‐), 159.0 (C‐4, C‐6), 156.2 (C‐2), 151.8 (C‐4′, C‐4″), 149.8 (C‐3′, C‐3″), 144.9 (C‐β, C‐β’), 127.7 (C‐1′, C‐1″), 126.9 (C‐α, C‐α’), 123.1 (C‐6′, C‐6″), 116.8 (C‐5′, C‐5″), 110.4 (C‐2′, C‐2″), 91.9 (C‐5), 62.8 (2‐OCH_3_), 55.64 (6‐OCH_3_, 4‐OCH_3_), 55.2 (4′‐OCH_3_, 4″‐OCH_3_, 3′‐OCH_3_, 3″‐OCH_3_). **MS** (ESI^+^) m/z (%): 549.3 [M+H]^+^ (35). **HRMS** (ESI^+^) m/z calcd for C_31_H_33_O_9_: 549.2046 [M+H]^+^; found: 549.2097.

**(*2E*,*2′E*)−1,1′‐(2,4,6‐Trimethoxy‐1,3‐phenylene)*bis*[3‐(4‐methoxyphenyl)prop‐2‐en‐1‐one]** (**11**), yellow solid, 74% yield (82% reported yield [21]). Full characterization is presented in the supporting information.

**(*2E*,*2′E*)−1,1′‐(2,4,6‐Trimethoxy‐1,3‐phenylene)*bis*[3‐(4‐hydroxyphenyl)prop‐2‐en‐1‐one]** (**12**) yellow solid, 21% yield, m.p. 219.5–220.7ºC, ^1^ **H NMR** (300 MHz, CDCl_3_) δ 7.57 (d, *J* = 8.6 Hz, 4H, H‐2′, 6′, H‐2″, 6″), 7.37 (d, *J* = 16.1 Hz, 2H, H‐α, H‐α’), 6.91 (d, *J* = 8.6 Hz, 4H, H‐3′, 5′, H‐3″, 5″), 6.87 (d, *J* = 16.1 Hz, 2H, H‐β, H‐β’), 6.68 (s, 1H, H‐5), 3.88 (s, 6H, 6‐OCH_3_, 4‐OCH_3_), 3.65 (s, 3H, 2‐OCH_3_). ^13^ **C NMR** (75 MHz, CDCl_3_) δ 192.6 (1‐COCH‐, 3‐COCH‐), 159.9 (C‐4′, C‐4″), 159.0 (C‐4, C‐6), 156.3 (C‐2), 144.7 (C‐β, C‐β’), 130.4 (C‐2′, 6′, C‐2″, 6″), 126.4 (C‐1′, C‐1″), 126.1 (C‐α, C‐α’), 116.8 (C‐3, C‐1), 115.9 (C‐3′, 5′, C‐3″, 5″), 91.9 (C‐5), 62.8 (2‐OCH_3_), 55.6 (6‐OCH_3_, 4‐OCH_3_). **MS** (ESI^+^) m/z (%): 461.2 [M + H]^+^ (33). **HRMS** (ESI^+^) m/z calcd for C_27_H_25_O_7_: 461.1595 [M + H]^+^; found: 461.1590.

**(*2E*,*2′E*)−1,1′‐(2,4,6‐Trimethoxy‐1,3‐phenylene)*bis*[3‐(4‐hydroxy‐3‐methoxyphenyl)prop‐2‐en‐1‐one]** (**13**) yellow solid, 8% yield, m.p. 122.0–123.1ºC. ^1^ **H NMR** (300 MHz, Acetone‐*d*_6_) δ 8.24 (s, 2H, 3′‐OH, 3″‐OH), 7.36 (d, *J* = 1.9 Hz, 2H, H‐2′, H‐2″), 7.35 (d, *J* = 16.1 Hz, 2H, H‐β, H‐β’), 7.17 (dd, *J* = 8.2, 1.9 Hz, 2H, H‐5′, H‐5″), 6.90 (d, *J* = 16.1 Hz, 2H, H‐α, H‐α’), 6.88 (d, *J* = 8.2 Hz, 2H, H‐6′, H‐6″), 6.69 (s, 1H, H‐5), 3.92 (s, 6H, 3′‐OCH_3_, 3″‐OCH_3_), 3.89 (s, 6H, 6‐OCH_3_, 4‐OCH_3_), 3.64 (s, 3H, 2‐OCH_3_). ^13^ **C NMR** (75 MHz, CDCl_3_) δ 194.0 (1‐COCH‐, 3‐COCH‐), 159.3 (C‐4, C‐6), 156.7 (C‐2), 148.4 (C‐4′, C‐4″), 146.8 (C‐3′, C‐3″), 146.1 (C‐β, C‐β’), 127.1 (C‐1´, C‐1´´), 126.4 (C‐α, C‐α’), 123.7 (C‐5′, C‐5″), 116.1 (C‐1′, C‐1″), 114.8 (C‐6′, C‐6″), 109.9 (C‐2′, C‐2″), 91.1 (C‐5), 63.2 (2‐OCH_3_), 56.2 (3′‐OCH_3_, 3″‐OCH_3_), 56.0 (6‐OCH_3_, 4‐OCH_3_). **MS** (ESI^+^) m/z (%): 521.2 [M+H]^+^ (100). **HRMS** (ESI^+^) m/z calcd for C_29_H_29_O_9_: 521.1806 [M+H]^+^; found: 521.1813.

**(*2E*,*2′E*)−1,1′‐(2,4,6‐Trimethoxy‐1,3‐phenylene)*bis*[3‐(4‐bromophenyl)prop‐2‐en‐1‐one]** (**14**) light yellow solid, 67% yield (62% reported yield [21]). Full characterization is presented in the supporting information.

**(*2E*,*2′E*)−1,1′‐(2,4,6‐Trimethoxy‐1,3‐phenylene)*bis*[3‐(3‐chlorophenyl)prop‐2‐en‐1‐one]** (**15**) light yellow solid, 60% yield, (50% reported yield [21]). Full characterization is presented in the supporting information.

**General procedure for the synthesis of 1,1′‐[2,4,6‐Tris(methoxymethoxy)−1,3‐phenylene]*bis*(ethan‐1‐one)** (**16a**)

Methoxymethyl chloride (MOMCl) (0.72 mL, 9.52 mmol) was slowly added to a solution of **2** (0.50 g, 2.38 mmol) in DCM (20 mL) with *N*, *N*‐diisopropylethylamine (DIPEIA) (4.14 mL, 23.80 mmol) at 0ºC. Then, the mixture was left stirring overnight at room temperature. The mixture was diluted with water, and the organic layer was separated and washed with saturated aqueous NaHCO_3_ solution and then with brine, dried with anhydrous sodium sulfate, and concentrated under reduced pressure. The resulting residue was purified by silica gel column chromatography using hexane/ethyl acetate (3:2) as eluent to yield compound **16 a** in 65% yield as a colorless oil.

**1,1′‐[2,4,6‐Tris(methoxymethoxy)−1,3‐phenylene]*bis*(ethan‐1‐one)** (**16a**) colorless oil, 65% yield. ^1^ **H NMR** (500 MHz, CDCl_3_) δ 6.76 (s, 1H, H‐5), 5.17 (s, 4H, 4‐OCH_2_OCH_3_, 6‐OCH_2_OCH_3_), 4.90 (s, 2H, 2‐OCH_2_OCH_3_), 3.46 (s, 6H, 4‐OCH_2_OCH_3_, 6‐OCH_2_OCH_3_), 3.39 (s, 3H, 2‐OCH_2_OCH_3_), 2.49 (s, 6H, 1‐COCH_3,_ 3‐COCH_3_). ^13^ **C NMR** (126 MHz, CDCl_3_) δ 200.9 (1‐COCH_3_, 3‐COCH_3_), 155.8 (C‐4, C‐6), 152.1 (C‐2), 121.4 (C‐3, C‐1), 102.0 (2‐OCH_2_‐), 97.8 (C‐5), 94.7 (4‐OCH_2_‐, 6‐OCH_2_‐), 57.3 (2‐OCH_2_OCH_3_), 56.6 (4‐OCH_2_OCH_3_, 6‐OCH_2_OCH_3_), 32.6 (1‐COCH_3_, 3‐COCH_3_).

#### 4.2.4 General Procedure for the Synthesis of 1,1′‐{2,4‐*Bis*[(2‐Methoxyethoxy)methoxy]‐6‐[(3‐Methoxypropoxy)methoxy]−1,3‐Phenylene}*Bis*(ethan‐1‐One) (16b)

2‐Methoxyethoxymethyl chloride (MEM‐Cl) (0.72 mL, 9.52 mmol) was slowly added to a solution of **2** (0.50 g, 2.38 mmol) in DCM (20 mL) with *N*, *N*‐diisopropylethylamine (DIPEIA) (4.14 mL, 23.80 mmol) at 0ºC. Then, the mixture was left stirring overnight at room temperature. The mixture was diluted with water, and the organic layer was separated and washed with saturated aqueous NaHCO_3_ solution and then with brine, dried with anhydrous sodium sulfate, and concentrated under reduced pressure. The resulting residue was purified by silica gel column chromatography using hexane/ethyl acetate (1:1) as eluent to yield compound **16.**

**1,1′‐{2,4‐*Bis*[(2‐methoxyethoxy)methoxy]‐6‐[(3‐methoxypropoxy)methoxy]−1,3‐phenylene}*bis*(ethan‐1‐one)** (**16b**). colorless oil, 60% yield. ^1^ **H NMR** (300 MHz, CDCl_3_) δ 6.83 (s, 1H, H‐5), 5.27 (s, 4H, 4‐OCH_2_OCH_2_, 6‐OCH_2_OCH_2_), 5.01 (s, 2H, 2‐OCH_2_OCH_2_), 3.82 – 3.77 (m, 4H, 4‐OCH_2_OCH_2_‐, 6‐OCH_2_OCH_2_‐), 3.75 – 3.71 (m, 2H, 2‐OCH_2_OCH_2_‐), 3.58 – 3.51 (m, 6H, 2‐OCH_2_OCH_2_CH_2_O‐, 4‐OCH_2_OCH_2_CH_2_O‐, 6‐OCH_2_OCH_2_CH_2_O‐), 3.38 (s, 6H, 4OCH_2_OCH_2_CH_2_OCH_3_, 6‐OCH_2_OCH_2_CH_2_OCH_3_), 3.37 (s, 3H, 2‐OCH_2_OCH_2_CH_2_OCH_3_), 2.50 (s, 6H, 1‐COCH_3_, 3‐COCH_3_). ^13^ **C NMR** (75 MHz, CDCl_3_) δ 200.9 (C‐3, C‐1), 155.8 (C‐4, C‐6), 152.0 (C‐2), 121.4 (C‐1, C‐3), 100.8 (2‐OCH_2_OCH_2_), 98.3 (C‐5), 93.8 (4‐OCH_2_OCH_2_, 6‐OCH_2_OCH_2_), 71.51 (2‐OCH_2_OCH_2_‐), 71.45 (4‐OCH_2_OCH_2_‐, 6‐OCH_2_OCH_2_‐), 69.2 (2‐OCH_2_OCH_2_ CH_2_O‐), 68.2 (4‐OCH_2_OCH_2_ CH_2_O‐, 6‐OCH_2_OCH_2_ CH_2_O‐), 59.0 (2‐OCH_2_OCH_2_CH_2_OCH_3_, 4‐OCH_2_OCH_2_CH_2_OCH_3_, 6‐OCH_2_OCH_2_CH_2_OCH_3_), 32.7 (1‐COCH_3_, 3‐COCH_3_).

#### 4.2.5 General Procedure for Synthesis of *Bis*‐Chalcones 19–25

Compound **16a/b** (1 mmol, 0.34/0.59 g) was dissolved in methanol (40 mL) and NaOH (4.0 g) was added and after 30 min of stirring at room temperature, the corresponding aldehyde (2.1 equiv) was added to the mixture. The mixture was stirred at room temperature for 24 – 48 h, then it was poured into ice and quenched with diluted hydrochloric acid to adjust the pH to 4. The product was extracted with DCM (3 × 20 mL). The organic layers were dried over anhydrous sodium sulfate, evaporated to dryness, and the residue was purified by silica gel column chromatography using a hexane/ethyl acetate 3:2 mixture as eluent.

**(*2E*,*2′E*)−1,1′‐[2,4,6‐Tris(methoxymethoxy)−1,3‐phenylene]*bis*[3‐(3,4‐dimethoxyphenyl)prop‐2‐en‐1‐one]** (**19**) yellow solid, 59% yield, m.p. 188.4–190.4ºC. ^1^ **H NMR** (300 MHz, Acetone‐*d*_6_) δ 7.43 (d, *J* = 16.1 Hz, 2H, H‐β, H‐β’), 7.37 (d, *J* = 2.0 Hz, 2H, H‐2′, H‐2″), 7.25 (dd, *J* = 8.5, 2.0 Hz, 2H, H‐6′, H‐6″), 7.00 (d, *J* = 16.1 Hz, 2H, H‐α, H‐α’), 6.99 (d, *J* = 8.5 Hz, 2H, H‐5′, H‐5″), 6.94 (s, 1H, H‐5), 5.25 (s, 4H, 4‐OCH_2_OCH_3_, 6‐OCH_2_OCH_3_), 4.91 (s, 2H, 2‐OCH_2_OCH_3_), 3.88 (s, 6H, 3′‐OCH_3_, 3″‐OCH_3_), 3.87 (s, 6H, 4′‐OCH_3_, 4″‐OCH_3_), 3.42 (s, 6H, 4‐OCH_2_OCH_3_, 6‐OCH_2_OCH_3_), 3.26 (s, 3H, 2‐OCH_2_OCH_3_). ^13^ **C NMR** (75 MHz, CDCl_3_) δ 193.4 (1‐COCH‐, 3‐COCH‐), 156.4 (C‐4, C‐6), 153.3 (C‐2), 151.5 (C‐4′, C‐4″), 149.3 (C‐3′, C‐3″), 145.7 (C‐β, C‐β’), 127.5 (C‐1′, C‐1″), 126.6 (C‐α, C‐α’), 123.2 (C‐6′, C‐6″), 119.5 (C‐3, C‐1), 111.1 (C‐5′, C‐5″), 110.0 (C‐2′, C‐2″), 100.8 (2‐OCH_2_‐), 98.1 (C‐5), 94.7 (4‐OCH_2_‐, 6‐OCH_2_‐), 57.3 (2‐OCH_2_OCH_3_), 56.6 (4‐OCH_2_OCH_3_, 6‐OCH_2_OCH_3_), 56.0 (3′‐OCH_3_, 3″‐OCH_3_), 55.9 (4′‐OCH_3_, 4″‐OCH_3_)_._ **MS** (ESI^+^) m/z (%): 638.8 [M+H] ^+^ (100).

**(*2E*,*2′E*)−1,1′‐[2,4,6‐Tris(methoxymethoxy)−1,3‐phenylene]*bis*[3‐(4‐methoxyphenyl)prop‐2‐en‐1‐one]** (**20**), yellow solid, 65% yield, m.p. 113.4–114.5ºC. ^1^ **H NMR** (500 MHz, CDCl_3_) δ 7.52 (d, *J* = 8.8 Hz, 4H, H‐2′, 6′, H‐2″, 6″), 7.40 (d, *J* = 16.0 Hz, 2H, H‐β, H‐β’), 6.91 (d, *J* = 8.8 Hz, 4H, H‐3′, 5′, H‐3″, 5″), 6.90 (d, *J* = 16.0 Hz, 2H, H‐α, H‐α’), 6.85 (s, 1H, H‐5), 5.17 (s, 4H, 4‐OCH_2_OCH_3_, 6‐OCH_2_OCH_3_,), 4.92 (s, 2H, 2‐OCH_2_OCH_3_), 3.84 (s, 6H, 4′‐OCH_3_, 4″‐OCH_3_), 3.42 (s, 6H, 4‐OCH_2_OCH_3_, 6‐OCH_2_OCH_3_), 3.28 (s, 3H, 2‐OCH_2_OCH_3_). ^13^ **C NMR** (126 MHz, CDCl_3_) δ 193.4 (1‐COCH‐, 3‐COCH‐), 161.7 (C‐4′, C‐4″), 156.4 (C‐4, C‐6), 153.4 (C‐2), 145.4 (C‐β, C‐β’), 130.3 (C‐2′, 6′, C‐2″, 6″), 127.3 (C‐1′, C‐1″), 126.4 (C‐α, C‐α’), 119.5 (C‐3, C‐1), 114.4 (C‐3′, 5′, C‐3″, 5″), 100.9 (2‐OCH_2_‐), 98.1 (C‐5), 94.7 (4‐OCH_2_‐, 6‐OCH_2_‐), 57.3 (2‐OCH_2_OCH_3_), 56.5 (4‐OCH_2_OCH_3_, 6‐OCH_2_OCH_3_), 55.4 (4′‐OCH_3_, 4″‐OCH_3_). **MS** (ESI^+^) m/z (%): 578.8 [M+H] ^+^ (100). **HRMS** (ESI^+^) m/z calcd for C_32_H_35_O_10_: 579.2225 [M+H]^+^; found: 579.2203.

**(*2E*,*2′E*)−1,1′‐[2,4,6‐Tris(methoxymethoxy)−1,3‐phenylene]*bis*{3‐[3‐methoxy‐4‐(methoxymethoxy)phenyl]prop‐2‐en‐1‐one}** (**21**), yellow oil, 60% yield. ^1^ **H NMR** (300 MHz, DMSO*‐d*_6_) δ 7.41 (d, *J* = 1.9 Hz, 2H, H‐2′, H‐2″), 7.35 (d, *J* = 16.1 Hz, 2H, H‐β, H‐β’), 7.27 (dd, *J* = 8.5, 1.9 Hz, 2H, H‐6′, H‐6′), 7.08 (d, *J* = 8.5 Hz, 2H, H‐5′, H‐5″), 7.07 (d, *J* = 16.1 Hz, 2H, H‐α, H‐α’), 6.87 (s, 1H, H‐6′, H‐6″), 5.29 – 5.18 (m, 8H, 4‐OCH_2_‐, 6‐OCH_2_‐, 4′‐OCH_2_‐, 4″‐OCH_2_), 4.80 (s, 2H, 2‐OCH_2_‐), 3.38 (s, 6H, 3′‐OCH_3_, 3″‐OCH_3_), 3.33 (s, 6H, 4‐OCH_2_OCH_3_, 6‐OCH_2_OCH_3_), 3.17 (s, 3H, 2‐OCH_2_OCH_3_). ^13^ **C NMR** (75 MHz, DMSO‐*d*_6_) δ 193.1 (1‐COCH‐, 3‐COCH‐), 156.0 (C‐4, C‐6), 152.6 (C‐2), 150.3 (C‐4′, C‐4″), 148.8 (C‐3′, C‐3″), 145.6 (C‐β, C‐β’), 129.0 (C‐1′, C‐1″), 127.5 (C‐α, C‐α’), 123.3 (C‐5′, C‐5″), 119.4 (C‐3, C‐1), 116.3 (C‐6′, C‐6″), 111.9 (C‐2′, C‐2″), 100.5 (2‐OCH_2_‐), 98.2 (C‐5), 94.92 and 94.71 (4′‐OCH_2_‐, 4″‐OCH_2_‐, 4‐OCH_2_‐, 6‐OCH_2_‐) 57.1 (2‐OCH_2_OCH_3_), 56.6 (4‐OCH_2_OCH_3_, 6‐OCH_2_OCH_3_), 56.3 (4′‐OCH_2_OCH_3_, 4″‐OCH_2_OCH_3_), 56.1 (3′‐OCH_3_, 3″‐OCH_3_). **MS** (ESI^+^) m/z (%): 699.3 [M+H] ^+^ (100); **HRMS** (ESI^+^) m/z calcd for C_36_H_43_O_14_: 699.2647 [M+H]^+^; found: 699.2639.

**(*2E*,*2′E*)−1,1′‐[2,4,6‐Tris(methoxymethoxy)−1,3‐phenylene]*bis*(3‐(4‐bromophenyl)prop‐2‐en‐1‐one)** (**22**) yellow solid, 67% yield, m.p. 128.9–131.1ºC. ^1^ **H NMR** (500 MHz, Acetone‐*d*_6_) δ 7.70 (d, *J* = 8.6 Hz, 4H, H‐2′, 6′, H‐2″, 6″), 7.64 (d, *J* = 8.5 Hz, 4H, H‐3′, 5′, H‐3″, 5″), 7.47 (d, *J* = 16.2 Hz, 2H, H‐β, H‐β’), 7.13 (d, *J* = 16.2 Hz, 2H, H‐α, H‐α’), 6.95 (s, 1H, H‐5), 5.26 (s, 4H, 4‐OCH_2_OCH_3_, 6‐OCH_2_OCH_3_), 4.89 (s, 2H, 2‐OCH_2_OCH_3_), 3.41 (s, 6H, 4‐OCH_2_OCH_3_, 6‐OCH_2_OCH_3_), 3.24 (s, 3H, 2‐OCH_2_OCH_3_). ^13^ **C NMR** (126 MHz, Acetone‐*d*_6_) δ 192.1 (1‐COCH‐, 3‐COCH‐), 156.6 (C‐4, C‐6), 153.2 (C‐2), 142.8 (C‐β, C‐β’), 134.2 (C‐1′, C‐1″), 132.1 (C‐3′, 5′, C‐3″, 5″), 130.2 (C‐2′, 6′, C‐2″, 6″), 129.4 (C‐α, C‐α’), 124.1 (C‐4′, C‐4″), 119.4 (C‐3, C‐1), 100.9 (2‐OCH_2_OCH_3_), 97.9 (C‐5), 94.8 (4‐OCH_2_OCH_3_, 6‐OCH_2_OCH_3_), 56.4 (2‐OCH_2_OCH_3_), 55.9 (4‐OCH_2_OCH_3_, 6‐OCH_2_OCH_3_). **MS** (ESI^+^) m/z (%): 676.2 [M+H]^+^ (^79^Br, 95), 678.5 [M+H]^+^ (^81^Br, 45). **HRMS** (ESI^+^) m/z calcd for C_30_H_29_Br_2_O_8_ : 677.0204 ([M+H]^+ 79^Br); found: 677.0212.

**(*2E*,*2′E*)−1,1′‐[2,4,6‐Tris(methoxymethoxy)−1,3‐phenylene]*bis*[3‐(3‐chlorophenyl)prop‐2‐en‐1‐one] (23)**, yellow solid, 47% yield, m.p. 135.6–137.4ºC. ^1^ **H NMR** (500 MHz, Acetone‐*d*_6_) δ 7.78 (d, *J* = 1.1 Hz, 2H, H‐2′, H‐2″), 7.68 (m, 2H, H‐5′, H‐5″), 7.50 (d, *J* = 16.3 Hz, 2H, H‐β, H‐β’), 7.46 (m, 4H, H‐4′, 6′, H‐4″, 6″), 7.17 (d, *J* = 16.3 Hz, 2H, H‐α, H‐α’), 6.97 (s, 1H, H‐5), 5.27 (s, 4H, 4‐OCH_2_OCH_3_, 6‐OCH_2_OCH_3_), 4.91 (s, 2H, 2‐OCH_2_OCH_3_), 3.42 (s, 3H, 4‐OCH_2_OCH_3_, 6‐OCH_2_OCH_3_), 3.26 (s, 3H, 2‐OCH_2_OCH_3_). ^13^ **C NMR** (126 MHz, Acetone‐*d*_6_) δ 192.1 (1‐COCH‐, 3‐COCH‐), 156.7 (C‐4, C‐6), 153.3 (C‐2), 142.5 (C‐β, C‐β’), 137.1 (C‐1′, C‐1″), 134.5 (C‐3′, C‐3^″^), 130.6 (C‐6′, C‐6″), 130.1 (C‐4′, C‐4″), 130.1 (C‐α, C‐α’), 128.1 (C‐2′, C‐2″), 126.8 (C‐5′, C‐5^″^), 119.3 (C‐3, C‐1), 100.9 (2‐OCH_2_OCH_3_), 98.0 (C‐5), 94.8 (4‐OCH_2_OCH_3_, 6‐OCH_2_OCH_3_), 56.5 (2‐OCH_2_OCH_3_), 55.9 (4‐OCH_2_OCH_3_, 6‐OCH_2_OCH_3_). **MS** (ESI^+^) m/z (%): 542.9 [M+H ‐ MOM]^+^ (^35^Cl, 100), 545.0 [M+H – MOM]^+^ (^37^Cl, 70).

**(*2E*,*2′E*)−1,1′‐{2,4,6‐Tris[(2‐methoxyethoxy)methoxy]−1,3‐phenylene}*bis*(3‐{3,4‐*bis*[(2‐methoxyethoxy)methoxy]phenyl}prop‐2‐en‐1‐one)** (**24**). colorless oil, 52% yield. ^1^ **H NMR** (300 MHz, CD_3_CN) δ 7.45 (d, *J* = 2.1 Hz, 2H, H‐2′, H‐2″), 7.36 (d, *J* = 16.1 Hz, 2H, H‐β, H‐β’), 7.28 (dd, *J* = 8.6, 2.1 Hz, 2H, H‐6′, H‐6″), 7.17 (d, *J* = 8.6 Hz, 2H, H‐5′, H‐5″), 6.95 (s, 1H, H‐5), 6.92 (d, *J* = 16.1 Hz, 2H, H‐α, H‐α’), 5.32 (s, 4H, 4‐OCH_2_‐, 6‐OCH_2_‐), 5.28 (s, 8H, 3′‐OCH_2_‐, 3″‐OCH_2_‐, 4′‐OCH_2_‐, 4″‐OCH_2_‐), 4.92 (s, 2H, 2‐OCH_2_‐), 3.86 – 3.75 (m, 8H, 3′‐OCH_2_OCH_2_‐, 4′‐OCH_2_OCH_2_‐, 3″‐OCH_2_OCH_2_‐, 4″‐OCH_2_OCH_2_‐), 3.76 – 3.67 (m, 4H, 4‐OCH_2_OCH_2_‐, 6‐OCH_2_OCH_2_‐), 3.61 – 3.42 (m, 2H, 2‐OCH_2_OCH_2_CH_2_O‐), 3.38 – 3.29 (m, 12H, 3′‐OCH_2_OCH_2_CH_2_O‐, 4′‐OCH_2_OCH_2_CH_2_O‐, 3″‐OCH_2_OCH_2_CH_2_O‐, 4″‐OCH_2_OCH_2_CH_2_O‐, 4‐OCH_2_OCH_2_CH_2_O‐, 6‐OCH_2_OCH_2_CH_2_O‐), 3.29 (s, 6H, 4′‐OCH_2_OCH_2_CH_2_OCH_3_, 4″‐OCH_2_OCH_2_CH_2_OCH_3_), 3.28 (s, 6H, 3′‐OCH_2_OCH_2_CH_2_OCH_3_, 3″‐OCH_2_OCH_2_CH_2_OCH_3_), 3.27 (s, 6H, 4‐OCH_2_OCH_2_CH_2_OCH_3_, 6‐OCH_2_OCH_2_CH_2_OCH_3_), 3.16 (s, 3H, 2‐OCH_2_OCH_2_CH_2_OCH_3_). ^13^ **C NMR** (75 MHz, CD_3_CN) δ 193.0 (1‐COCH‐, 3‐COCH‐), 156.3 (C‐4, C‐6), 152.5 (C‐2), 145.0 (C‐4′, C‐4″), 147.3 (C‐3′, C‐3″), 145.1 (C‐β, C‐β’), 128.8 (C‐1′, C‐1″), 127.4 (C‐α, C‐α’), 124.2 (C‐6′, C‐6″), 119.2 (C‐1, C‐3), 117.1 (C‐2′, C‐2″), 116.7 (C‐5′, C‐5″), 99.5 (C‐5), 94.44 (4′‐OCH_2_O‐, 4″‐OCH_2_O‐), 93.9 (3′‐OCH_2_O‐, 3″‐OCH_2_O‐), 93.8 (4‐OCH_2_O‐, 6‐OCH_2_O‐), 71.3 (4′‐OCH_2_OCH_2_ CH_2_O‐, 4″‐OCH_2_OCH_2_ CH_2_O‐), 71.3 (3′‐OCH_2_OCH_2_ CH_2_O‐, 3″‐OCH_2_OCH_2_ CH_2_O‐), 71.2 (4‐OCH_2_OCH_2_ CH_2_O‐, 6‐OCH_2_OCH_2_ CH_2_O‐), 71.1 (2‐OCH_2_OCH_2_ CH_2_O‐), 68.8 (2‐OCH_2_OCH_2_‐), 68.1 (4′‐OCH_2_OCH_2_‐, 4″‐OCH_2_OCH_2_‐), 68.0 (3′‐OCH_2_OCH_2_‐, 3″‐OCH_2_OCH_2_‐), 67.90 (4‐OCH_2_OCH_2_‐, 6‐OCH_2_OCH_2_‐), 57.9 (3′‐OCH_2_OCH_2_CH_2_OCH_3_, 3″‐OCH_2_OCH_2_CH_2_OCH_3_, 4′‐OCH_2_OCH_2_CH_2_OCH_3_, 4″‐OCH_2_OCH_2_CH_2_OCH_3_), 57.9 (4‐OCH_2_OCH_2_CH_2_OCH_3_, 6‐OCH_2_OCH_2_CH_2_OCH_3_), 57.8 (2‐OCH_2_OCH_2_CH_2_OCH_3_). **MS** (ESI^+^) m/z (%): 1067.5 [M+H] ^+^ (80). **HRMS** (ESI^+^) m/z calcd for C_52_H_75_O_23_: [M+H]^+^ 1067.4694; found: 1067.4692.

**(*2E*,*2′E*)−1,1′‐[2,4,6‐Tris(methoxymethoxy)−1,3‐phenylene]*bis*{3‐[4‐(methoxymethoxy)phenyl]prop‐2‐en‐1‐one}** (**25**) yellow oil, 59% yield, ^1^ **H NMR** (500 MHz, Acetone‐*d*_6_) δ 7.67 (d, *J* = 8.8 Hz, 4H, H‐2′, 6′, H‐2″, 6″), 7.47 (d, *J* = 16.1 Hz, 2H, H‐β, H‐β’), 7.10 (d, *J* = 8.8 Hz, 4H, H‐3′, 5′, H‐3″, 5″), 7.01 (d, *J* = 16.1 Hz, 2H, H‐α, H‐α’), 6.97 (s, 1H, H‐5), 5.26 (s, 8H, 4‐OCH_2_OCH_3_, 6‐OCH_2_OCH_3_, 4′‐OCH_2_OCH_3_, 4″‐OCH_2_OCH_3_), 4.92 (s, 2H, 2‐OCH_2_OCH_3_), 3.44 (s, 6H, 6‐OCH_2_OCH_3_, 4′‐OCH_2_OCH_3_), 3.41 (s, 6H, 4′‐OCH_2_OCH_3_, 4″‐OCH_2_OCH_3_), 3.26 (s, 3H, 2‐OCH_2_‐OCH_3_). ^13^ **C NMR** (126 MHz, Acetone‐*d*_6_) δ 192.3 (1‐COCH‐, 3‐COCH‐), 159.4 (C‐4′, C‐4″), 156.3 (C‐4, C‐6), 153.0 (C‐2), 144.4 (C‐β, C‐β’), 130.2 (C‐2′, 6′, C‐2″, 6″), 128.4 (C‐1′, C‐1″), 127.1 (C‐α, C‐α’), 119.7 (C‐3, C‐1), 116.5 (C‐3′, 5′, C‐3″, 5″), 100.8 (2‐OCH_2_‐), 98.0 (C‐5), 94.7 (4′‐OCH_2_, 4″‐OCH_2_), 94.0 (4‐OCH_2_, 6‐OCH_2_) 56.5 (2‐OCH_2_OCH_3_), 55.9 (4′‐OCH_2_OCH_3_, 4″‐OCH_2_OCH_3_), 55.4 (4‐OCH_2_OCH_3_, 6‐OCH_2_OCH_3_). **MS** (ESI^+^) m/z (%): 639.2 [M+H] ^+^ (40). **HRMS** (ESI^+^) m/z calcd for C_34_H_39_O_12_: 639.2436 [M+H]^+^; found: 639.2421.

#### 4.2.6 General Procedure for the Synthesis of 26–33

Trifluoroacetic acid (TFA) (1 mL) was added to a solution of **26–32** (0.5 mmol) in dry DCM (10 mL) at 0ºC. The mixture was stirred for 30–60 min. until total consumption of the starting material, controlled by TLC. The mixture was treated with methanol, followed by evaporation until the total removal of TFA.

**(*2E*,*2′E*)−1,1′‐(2,4,6‐Trihydroxy‐1,3‐phenylene)*bis*[3‐(3,4‐dimethoxyphenyl)prop‐2‐en‐1‐one]** (**26**) red solid, 99% yield, m.p. 160.0–160.4ºC. ^1^ **H NMR** (300 MHz, DMSO*‐d*_6_) δ 16.82 (s, 1H, 2‐OH), 13.27 (s, 2H, 4‐OH, 6‐OH), 7.95 (d, *J* = 15.6 Hz, 2H, H‐α, H‐α’), 7.74 (d, *J* = 15.6 Hz, 2H, H‐β, H‐β’), 7.31 (d, *J* = 6.7 Hz, 4H, H‐2′, 6′, H‐2″, 6″), 7.04 (d, *J* = 8.6 Hz, 2H, H‐5′, H‐5″), 5.99 (s, 1H, H‐5), 3.83 (s, 6H, 4′‐OCH_3_, 4″‐OCH_3_), 3.82 (s, 6H, 3′‐OCH_3_, 3″‐OCH_3_). ^13^ **C NMR** (75 MHz, DMSO*‐d*_6_) δ 192.7 (1‐COCH‐, 3‐COCH‐), 171.4 (C‐2), 168.4 (C‐4, C‐6), 151.8 (C‐4′, C‐4″), 149.4 (C‐3′, C‐3″), 144.2 (C‐β, C‐β’), 128.0 (C‐1′, C‐1″), 124.9 (C‐α, C‐α’), 123.5 (C‐6′, C‐6″), 112.2 (C‐5′, C‐5″), 111.4 (C‐2′, C‐2″), 104.7 (C‐3, C‐1), 95.6 (C‐5), 56.1 (3′‐OCH_3_, 3″‐OCH_3_), 55.9 (4′‐OCH_3_, 4″‐OCH_3_). **MS** (ESI^+^) m/z (%): 507.1 [M+H] ^+^ (100). **HRMS** (ESI^+^) m/z calcd for C_28_H_27_O_9_: 507.1650 [M+H]^+^; found: 507.1639.

**(*2E*,*2′E*)−1,1′‐(2,4,6‐Trihydroxy‐1,3‐phenylene)*bis*[3‐(4‐methoxyphenyl)prop‐2‐en‐1‐one]** (**27**) Orange solid, 97% yield, m.p. 234.4–236.3ºC. ^1^ **H NMR** (300 MHz, DMSO*‐d*_6_) δ 16.93 (s, 1H, 2‐OH), 13.34 (s, 2H, 4‐OH, 6‐OH), 7.96 (d, *J* = 15.7 Hz, 2H, H‐β, H‐β’), 7.77 (d, *J* = 15.7 Hz, 2H, H‐α, H‐α’), 7.70 (d, *J* = 8.7 Hz, 4H, H‐2′, 6′, H‐2″, 6″), 7.03 (d, *J* = 8.7 Hz, 4H, H‐3′, 5′, H‐3″, 5″), 5.99 (s, 1H, H‐5), 3.82 (s, 6H, 4′‐OCH_3_, 4″‐OCH_3_). ^13^ **C NMR** (75 MHz, DMSO*‐d*_6_) δ 192.7 (1‐COCH‐, 3‐COCH‐), 171.7 (C‐2), 168.6 (C‐4, C‐6), 161.9 (C‐4′, C‐4″), 143.8 (C‐β, C‐β’), 131.0 (C‐2′, 6′, C‐2″, 6″), 127.8 (C‐1′, C‐1″), 124.7 (C‐α, C‐α’), 115.1 (C‐3′, 5′, C‐3″, 5″), 104.7 (C‐3, C‐1), 95.6 (C‐5), 55.9 (4′‐OCH_3_, 4″‐OCH_3_). **MS** (ESI^+^) m/z (%): 491.0 [M + 2Na‐H]^+^ (100), **HRMS** (ESI^+^) m/z calcd for C_26_H_23_O_7_: 447.1438 [M+H]^+^; found: 447.1432.

**(*2E*,*2′E*)−1,1′‐(2,4,6‐Trihydroxy‐1,3‐phenylene)*bis*[3‐(4‐hydroxy‐3‐methoxyphenyl)prop‐2‐en‐1‐one]** (**28**), red solid, 97% yield, m.p. 156.2–157.4ºC. ^1^ **H NMR** (300 MHz, DMSO*‐d*_6_) δ 16.90 (s, 1H, 2‐OH), 13.30 (s, 2H, 4‐OH, 6‐OH), 9.79 (s, 2H, 4′‐OH, 4″‐OH), 7.93 (d, *J* = 15.5 Hz, 2H, H‐α, H‐α’), 7.74 (d, *J* = 15.5 Hz, 2H, H‐β, H‐β’), 7.30 (d, *J* = 2.0 Hz, 2H, H‐2′, H‐2″), 7.22 (dd, *J* = 8.2, 2.0 Hz, 2H, H‐6′, H‐6″), 6.86 (d, *J* = 8.2 Hz, 2H, H‐5′, H‐5″), 5.99 (s, 1H, H‐5), 3.85 (s, 6H, 3′‐OCH_3_, 3″‐OCH_3_). ^13^ **C NMR** (75 MHz, DMSO*‐d*_6_) δ 192.7 (1‐COCH‐, 3‐COCH‐), 168.4 (C‐4, C‐6), 150.3 (C‐4′, C‐4″), 148.4 (C‐3′, C‐3″), 144.8 (C‐β, C‐β’), 126.8 (C‐1′, C‐1″), 123.9 (C‐α, C‐α’), 123.7 (C‐6′, C‐6″), 116.3 (C‐5′, C‐5″), 112.5 (C‐2′, C‐2″), 104.7 (C‐3, C‐1), 95.5 (C‐5), 56.1 (3′‐OCH_3_, 3″‐OCH_3_). **MS** (ESI^+^) m/z (%): 479.1 [M+H] ^+^ (100), **HRMS** (ESI^+^) m/z calcd for C_26_H_23_O_9_: 479.1337 [M+H]^+^; found: 479.1317.

**(*2E*,*2′E*)−1,1′‐(2,4,6‐Trihydroxy‐1,3‐phenylene)*bis*[3‐(4‐bromophenyl)prop‐2‐en‐1‐one]** (**29**) yellow solid, 99% yield, m.p. 224.0–225.6ºC. ^1^ **H NMR** (500 MHz, DMSO*‐d*_6_) δ 16.54 (s, 1H, 2‐OH), 13.20 (s, 2H, 4‐OH, 6‐OH), 8.03 (d, *J* = 15.7 Hz, 1H, H‐β, H‐β’), 7.71 (d, *J* = 15.7 Hz, 1H, H‐α, H‐α’), 7.69 – 7.65 (m, 8H, H‐3′, 5′, H‐2′, 6′, H‐3″, 5″, H‐2″, 6″), 6.01 (s, 1H, H‐5). ^13^ **C NMR** (126 MHz, CDCl_3_) δ 192.7 (1‐COCH‐, 3‐COCH‐), 171.4 (C‐4, C‐6), 168.6 (C‐2), 142.0 (C‐β, C‐β’), 134.5 (C‐1′, C‐1″), 132.5 (C‐3′, 5′, C‐3″, 5″), 130.9 (C‐2′, 6′, C‐2″, 6″), 128.1 and 129.0 (C‐α, C‐α’), 124.4 (C‐4′, C‐4″), 104.9 (C‐3, C‐1), 95.7 (C‐5). **MS** (ESI^‐^) m/z (%): 542.9 [M‐H]^−^ (^79^Br, 95), 544.9 [M‐H]^−^ (^81^Br, 45). **HRMS** (ESI^‐^) m/z calcd for C_24_H_15_Br_2_O_5_: 542.9271 ([M‐H]^− 81^Br); found: 542.9290.

**(*2E*,*2′E*)−1,1′‐(2,4,6‐Trihydroxy‐1,3‐phenylene)*bis*[3‐(3‐chlorophenyl)prop‐2‐en‐1‐one]** (**30**) yellow solid, 96% yield, m.p. 146.6–148.0ºC. ^1^ **H NMR** (300 MHz, DMSO*‐d*_6_) δ 16.33 (s, 1H, 2‐OH), 13.10 (s, 2H, 4‐OH, 6‐OH), 8.03 (d, *J* = 15.8 Hz, 2H, H‐α, H‐α’), 7.82 (d, *J* = 2.1 Hz, 2H, H‐2′, H‐2″), 7.75 – 7.67 (m, 2H, H‐β, H‐β’, H‐4′, H‐4″), 7.55 – 7.46 (m, 4H, H‐5′, 6′, H‐5″, 6″), 6.02 (s, 1H, H‐5). ^13^ **C NMR** (75 MHz, DMSO*‐d*_6_) δ 192.7 (1‐COCH‐, 3‐COCH‐), 141.4 (C‐β, C‐β’), 137.5 (C‐1′, C‐1″), 134.3 (C‐3′, C‐3″), 131.4 (C‐5′, C‐5″), 130.6 (C‐6′, C‐6″), 129.1 (C‐α, C‐α’), 128.6 (C‐2′, C‐2″), 127.4 (C‐4′, C‐4″), 105.0 (C‐3, C‐1), 96.0 (C‐5). **MS** (ESI‐) m/z (%): 453.0 [M‐H]^−^ (100). **HRMS** (ESI^‐^) m/z calcd for C_24_H_15_Cl_2_O_5_: 454.0302 [M‐H]^‐^; found: 454.0319.

**(*2E*,*2′E*)−1,1′‐(2,4,6‐Trihydroxy‐1,3‐phenylene)*bis*[3‐(3,4‐dihydroxyphenyl)prop‐2‐en‐1‐one]** (**31**), dark red solid, 98% yield, m.p. ≥ 230ºC decomposition. ^1^ **H NMR** (300 MHz, DMSO*‐d*_6_) δ 17.23 (s, 1H, 2‐OH), 13.47 (s, 2H, 4‐OH, 6‐OH), 9.73 (s, 2H, 4′‐OH, 4″‐OH), 9.33 (s, 2H, 3′‐OH, 3″‐OH), 7.88 (d, *J* = 15.5 Hz, 2H, H‐β, H‐β’), 7.68 (d, *J* = 15.5 Hz, 2H, H‐α, H‐α’), 7.14 (d, *J* = 2.1 Hz, 2H, H‐2′, H‐2″), 7.05 (dd, *J* = 8.2, 2.1 Hz, 2H, H‐6′, H‐6″), 6.81 (d, *J* = 8.2 Hz, 2H, H‐5′, H‐5″), 5.98 (s, 1H, H‐5). ^13^ **C NMR** (75 MHz, Acetone‐*d*_6_) δ 193.0 (1‐COCH‐,3‐COCH‐), 148.5 (C‐4′, C‐4″), 145.6 (C‐3′, C‐3″), 144.3 (C‐β, C‐β’), 127.6 (C‐α, C‐α’), 122.7 (C‐6′, C‐6″), 115.7 (C‐5′, C‐5″), 114.5 (C‐2′, C‐2″), 103.5 (C‐5). **MS** (ESI^+^) m/z (%): 451.1 [M+H] ^+^ (25).

**(*2E*,*2′E*)−1,1′‐(2,4,6‐Trihydroxy‐1,3‐phenylene)*bis*[3‐(4‐hydroxyphenyl)prop‐2‐en‐1‐one]** (**32**) red solid, 99% yield, m.p. 182.7–185.5ºC. ^1^ **H NMR** (300 MHz, DMSO*‐d*_6_) δ 17.05 (s, 1H, 2‐OH), 13.41 (s, 2H, 4‐OH, 6‐OH), 10.14 (s, 2H, 4′‐OH, 4″‐OH) 7.93 (d, *J* = 15.6 Hz, 2H, H‐α, H‐α’), 7.74 (d, *J* = 15.6 Hz, 2H, H‐β, H‐β’), 7.59 (d, *J* = 8.6 Hz, 4H, H‐2′, 6′, H‐2″, 6″), 6.86 (d, *J* = 8.6 Hz, 4H, H‐3′, 5′, H‐3″, 5″), 6.02 (s, 1H, H‐5). ^13^ **C NMR** (75 MHz, DMSO*‐d*_6_) δ 192.7 (1‐COCH‐, 3‐COCH‐), 171.8 (C‐2), 168.6 (C‐4, C‐6), 160.8 (C‐4′, C‐4″), 144.4 (C‐β, C‐β’), 131.3 (C‐2′, 6′, C‐2″, 6″) 126.3 (C‐1′, C‐1″), 123.5 (C‐α, Cα’), 116.5 (C‐3′, 5′, C‐3″, 5″), 104.6 (C‐3, C‐1), 95.6 (C‐5). **MS** (ESI^‐^) m/z (%): 417.1 [M‐H] ^‐^ (100). **HRMS** (ESI^‐^) m/z calcd for C_24_H_17_O_7_: 417.0979 [M‐H]^−^; found: 417.0992.

### 4.3 COX‐1/2 Inhibition Activity

Celecoxib, 5‐(4‐chlorophenyl)‐1‐(4‐methoxyphenyl)‐3‐trifluoromethylpyrazole (SC‐560), dimethylsulfoxide (DMSO) and ethanol absolute were obtained from Fisher Chemical (Loughborough, Leics, UK). The following reagents were obtained from Sigma Chemical Co. (St. Louis, MO, USA): potassium phosphate monobasic (KH_2_PO_4_), sodium phosphate dibasic (Na_2_HPO_4_), hemin, ampliflu red, human recombinant COX‐1 and COX‐2, trizma hydrochloride, ethylenediaminetetraacetic acid (EDTA) disodium salt dihydrate. Potassium chloride (KCl) and sodium chloride (NaCl) were obtained from Pronalab (Abrunheira, Portugal) and VWR Chemicals (Alfragide, Portugal), respectively. Peroxide‐free AA was obtained from Cayman Chemicals (Ann Arbor, MI, USA).

### 4.4 In Vitro COX‐1/2 Inhibitory Assay

Human recombinant COX‐1 or COX‐2 enzymes oxidize the substrate AA yielding PGG_2_, which is further transformed by COX (peroxidase site of COX) alongside a fluorescence probe (ampliflu red), resulting in the production of PGH_2_ and resorufin, the latter being the product responsible for the fluorescence detected at λ_Em/Ex_ = 535/587 nm. Before the experiment, absorption spectra were traced for all *bis*‐chalcones (**10–15**, **19–25**, **26–31**) to guarantee that none of them interfered with the assays at the wavelengths used.

To conduct the assay, human recombinant COX‐1 or COX‐2 (2.5 ng/µL) was combined with diluted hemin (1 µM), COX cofactor, and ampliflu red (100 µM) in assay buffer (trizma hydrochloride pH = 8.0 and EDTA 0.1 µM). Subsequently, 85 μL of this reaction mixture were added to a 96‐well black plate containing 5 μL of compound (0 ‐ 100 μM) or DMSO and incubated for 5 min. Following this, 10 μL of diluted AA solution (25 µM) were added, and immediately proceeded to measure fluorescence for 15 min at 25°C using a microplate reader (Synergy HT, BIO‐TEK, Winooski, VT, USA) with excitation at 530 ± 25 nm and emission detection at 590 ± 35 nm. Celecoxib (0 ‐ 1 μM) was employed as the positive control for COX‐2, and SC‐560 (0–40 nM) was the positive control used for COX‐1. The amount of DMSO used [5% (v/v)] did not interfere with the assay, and none of the tested compounds showed fluorescence emission in the specified wavelength ranges of the assay. COX activity inhibitions were expressed as the percentage of inhibition of COX activity, calculated using a single point of relative fluorescence unit (RFU) after 5 min of reading. These results were obtained from a minimum of three distinct and independent experiments.

The selective index (SI) is a calculated parameter used to quickly evaluate the selectivity of a given compound to COX‐2 over COX‐1. These values were calculated according to the following formulas. For compounds presenting IC_50_, the calculation was performed according to the following equation:

(1) $$\mathrm{SI}=\frac{IC_{50}^{\mathrm{COX}-1}}{IC_{50}^{\mathrm{COX}-2}}$$

For compounds presenting inhibition at just 100 µM, the highest tested concentration, the calculation was proceeded according to the following equation:

(2) $$SI=\frac{\%\mathrm{Inib}._{x}^{\mathrm{COX}-2}}{\%\mathrm{Inib}._{x}^{\mathrm{COX}-1}}$$

For both formulas, a high SI value means higher selectivity for COX‐2.

### 4.5 Inhibitory Kinetic Analysis of COX‐1/2

The inhibition mechanism of both COX‐1 and COX‐2 enzymes was identified for the most active compound found in each screening assay. Specifically, for COX‐1, compound **30** was tested (IC_50_ = 2.0 µM), and for COX‐2, it was compound **31** (IC_50_ = 1.5 µM). To do so, a similar protocol to the in vitro COX inhibition assay was used. In a 96 well plate, these compounds, at different concentrations (**30**: 8, 10, 12.5 and 15 µM; **31**: 0.2, 0.78 and 3.1 µM), were incubated with the respective enzyme for 5 min, and three different concentrations of AA were tested (12.5, 25 and 100 µM) and the fluorescence generated was recorded using the same prior protocol.

The kinetics of the system were described using the generalized Michaelis‐Menten equation (Equation (3)), with various simplifications corresponding to different types of inhibition:

(3) $$v_{\mathrm{inic}}=\frac{V_{m´ax}(S)}{K_{m}\left(1+\frac{\left[I\right]}{K_{\mathrm{ic}}}\right)+(S)(1+\frac{\left[I\right]}{K_{\mathrm{iu}}})}$$

where *v*_inic_ = initial velocity of formation of resorufin in ΔRFU per minute, *V*_max_ = maximum achievable velocity when for the 2.5 ng/µL of enzyme had used all the catalytic sites and is saturated by the substrate, *S* = AA concentration in mM, *K*_m_ = Michaelis–Menten constant in μM, *K*_ic_ = inhibitor dissociation constant of enzyme inhibitor expressed in μM^−1^, *K*_iu_ = inhibitor dissociation constant of enzyme–substrate–inhibitor complex expressed in μM^−1^.

Each nonlinear regression of the results was conducted based on the data from a minimum of three independent experiments. Microsoft Excel augmented with the Solver add‐in was employed for the nonlinear regression analysis, according to the guidelines described by Bezerra et al. [29] and Dias et al. [35].

In each case, the parameters of the equation were estimated using Solver, with initial guesses based on the parameter values obtained from the simplest model (absence of inhibition). These initial estimates were then used for the models corresponding to competitive, noncompetitive, uncompetitive, and mixed inhibition.

To determine the actual mechanism of inhibition, the models were compared using the extra sum‐of‐squares F test and the Akaike information criterion (AIC) test. The error associated with the kinetic constant values was assessed using the Jackknife procedure, which involved calculating the standard deviation of parameter estimates obtained from Solver when each experimental data point was omitted iteratively.

Additionally, Lineweaver‐Burk plots were analyzed for each concentration of inhibitor and substrate to provide further insights into the kinetics of the system. The plots were traced using the linearized version of Equation (3) (Equation (4)).

(4) $$\frac{1}{v_{\mathrm{inic}}}=\frac{K_{m}}{V_{\max}}\times \frac{1}{\left[S\right]}+\frac{1}{V_{\max}}$$

### 4.6 Statistical Analysis

The results of the in vitro tested inhibitory activities of the tested compounds are expressed as half maximal inhibitory concentration (IC_50_). Results are presented as mean ± standard error of the mean (SEM) (*n *≥ 3). Statistical analysis was carried out using GraphPad Prism (version 8.0; GraphPad Software, San Diego, CA, USA). Group comparisons were evaluated using one‐way analysis of variance (ANOVA), followed by Dunnett's multiple comparison test. Statistical significance was established at *p *< 0.05.

## 5 Molecular Docking Studies

### 5.1 Proteins and Ligands Preparation

We retrieved all available 3D experimental structures of human and sheep COX‐1 (UniProt IDs P23219 and P05979, respectively), which share similar binding pockets. The corresponding PDB entries were then filtered to identify those containing ligands in the secondary pocket/hydrophobic region [36], resulting in a final list that includes one human PDB (apo) structure, 6Y3C (UniProt ID P23219), and 15 PDBs of sheep COX‐1 structures (UniProt IDs P05979): 1CQE, 1DIY, 1EBV, 1EQG, 1HT5, 1HT8, 1IGX, 1IGZ, 1PGE, 1Q4G, 3KK6, 3N8X, 401Z, 5U6X, 5WBE. Given the limited availability of human structures, sheep COX‐1 structures were included but used only to validate the docking protocol. Regarding COX‐2 (UniProt ID P23219), we retrieved six human holo structures (PDB IDs: 5IKT, 5IKR, 5KIR, 5IKV, 5IKQ, and 5F1A), making it unnecessary to include non‐human structures. These complexes were stripped of all small molecules except for the ligand. Subsequently, all structures underwent preparation using the Molecular Operating Environment (MOE 2020) [37], where minor issues, such as atoms with fractional occupancies, were addressed. The protein–ligand complexes were protonated using the Protonate3D tool implemented in MOE 2020. Ligands were separated from their corresponding proteins, and both 3D structures were saved in separate files. All ligands were energy minimized in MOE using the AMBER10:EHT force.

### 5.2 Docking Protocol Validation

Initially, a docking validation step was conducted, involving self‐ and cross‐docking calculations (with all co‐crystals for all selected PDBs) using the LeDock 1.0, GNINA 1.0, SMINA 1.0, and Glide 1.0 software packages, along with an exhaustive search of 1,000 runs. Optimal docking parameters, including docking software and protein 3D structures, were selected according to the most experimental ligand poses successfully reproduced (RMSD ≤ 2 Å between experimental and predicted poses). This criterion was met using Glide software applied to the PDB IDs: 6Y3C (COX‐1) and 5IKT (COX‐2) structures.

### 5.3 Data Analysis and Image Generation

To assess protein–ligand interactions, the top docking poses were analyzed for residue interactions using the Docker implementation of PLIP [38]. Data plots were produced using the seaborn Python data visualization library [39], while compound structures were managed and analyzed using BioPandas [40], fconv (for RMSD calculations) [41] and RDKit [42]. Docking results were processed, and trends were analyzed using a workflow implemented in Jupyter Notebook [43].

## Supporting Information

Additional supporting information can be found online in the Supporting Information section.

## Funding

This work was supported by Portuguese national funds through Fundação para a Ciência e a Tecnologia (FCT/MECI – Ministério da Educação, Ciência e Inovação) under project UID/50006/2025 (https://doi.org/10.54499/UID/50006/2025), within the framework of the Laboratório Associado para a Química Verde – Tecnologias e Processos Limpos. The authors also acknowledge the funding from the doctoral grant UI/BD/151269/2021.

## Conflicts of Interest

The authors declare no conflicts of interest.

## Supporting information

Supplementary Material

## Data Availability

The data that support the findings of this study are available in the supplementary material of this article.
