# Supplementary material for: Design, Synthesis, and Evaluation of New Polyhydroxylated B is‐Chalcones as Potential COX‐2 Selective Inhibitors
Source: ChemMedChem. 2026 Apr 25;21(8):e202500784. doi: 10.1002/cmdc.202500784 (PMC13110330; doi:10.1002/cmdc.202500784)
Supplement: Supplementary file 1 — Supplementary Material [file CMDC-21-e202500784-s001.pdf]

Design, Synthesis and Evaluation of New Polyhydroxylated Bis-Chalcones as Potential COX-2 Selective Inhibitors

Rui Pereira [1], Alberto N. Araújo [1], Daniela Ribeiro [1,2], Ismael Rufino [3], Nuno Martinho [3], Rita C. Guedes [3], Vera L. M. Silva [4], Eduarda Fernandes [1]

1- LAQV-REQUIMTE, Laboratory of Applied Chemistry, Department of Chemical Sciences, Faculty of Pharmacy, University of Porto, Rua de Jorge Viterbo Ferreira nº 228, 4050-313 Porto, Portugal;

2- Faculty of Agrarian Sciences and Environment, University of the Azores, 9700-042 Angra do Heroísmo, Açores, Portugal.

3- Research Institute for Medicines (iMed.ULisboa), Faculty of Pharmacy, University of Lisboa, Av. Prof. Gama Pinto, 1649-003 Lisboa, Portugal;

4- LAQV-REQUIMTE, Department of Chemistry, University of Aveiro, 3810-193 Aveiro, Portugal;

Figure S1: ^{1}H NMR spectrum of compound **2** (300 MHz, Acetone-d_6). 4
Figure S2: ^{13}C NMR spectrum of compound **2** (75 MHz, Acetone-d_6). 4
Figure S3: ^{1}H NMR spectrum of compound **3** (300 MHz, Acetone-d_6). 5
Figure S4: ^{13}C NMR spectrum of compound **3** (75 MHz, Acetone-d_6). 5
Figure S5: ^{1}H NMR spectrum of compound **10** (300 MHz, Acetone-d_6). 6
Figure S6: ^{13}C NMR spectrum of compound **10** (75 MHz, Acetone-d_6). 6
Figure S7: ^{1}H NMR spectrum of compound **11** (300 MHz, Acetone-d_6). 7
Figure S8: ^{13}C NMR spectrum of compound **11** (75 MHz, Acetone-d_6). 7
Figure S9: ^{1}H NMR spectrum of compound **12** (300 MHz, Acetone-d_6). 9
Figure S10: ^{13}C NMR spectrum of compound **12** (75 MHz, Acetone-d_6). 9
Figure S11: ^{1}H NMR spectrum of compound **13** (300 MHz, $CDCl_3$). 10
Figure S12: ^{13}C NMR spectrum of compound **13** (75 MHz, $CDCl_3$). 10
Figure S13: ^{1}H NMR spectrum of compound **14** (300 MHz, Acetone-d_6). 11
Figure S14: ^{13}C NMR spectrum of compound **14** (75 MHz, Acetone-d_6). 11
Figure S15: ^{1}H NMR spectrum of compound **15** (300 MHz, $CDCl_3$). 13
Figure S16: ^{13}C NMR spectrum of compound **15** (75 MHz, $CDCl_3$). 13
Figure S17: ^{1}H NMR spectrum of compound **16a** (300 MHz, $CDCl_3$). 15
Figure S18: ^{13}C NMR spectrum of compound **16** (75 MHz, $CDCl_3$). 15
Figure S19: ^{1}H NMR spectrum of compound **16b** (300 MHz, $CDCl_3$). 16
Figure S20: ^{13}C NMR spectrum of compound **16b** (75 MHz, $CDCl_3$). 16
Figure S21: ^{1}H NMR spectrum of compound **19** (300 MHz, $CDCl_3$). 17
Figure S22: ^{13}C NMR spectrum of compound **19** (75 MHz, $CDCl_3$). 17
Figure S23: ^{1}H NMR spectrum of compound **20** (500 MHz, $CDCl_3$). 18
Figure S24: ^{13}C NMR spectrum of compound **20** (126 MHz, $CDCl_3$). 18
Figure S25: ^{1}H NMR spectrum of compound **21** (300 MHz, Acetone-d_6). 19
Figure S26: ^{13}C NMR spectrum of compound **21** (75 MHz, Acetone-d_6). 19
Figure S27: ^{1}H NMR spectrum of compound **22** (300 MHz, Acetone-d_6). 20
Figure S28: ^{13}C NMR spectrum of compound **22** (75 MHz, Acetone-d_6). 20
Figure S29: ^{1}H NMR spectrum of compound **23** (300 MHz, Acetone-d_6). 21
Figure S30: ^{13}C NMR spectrum of compound **23** (75 MHz, Acetone-d_6). 21
Figure S31: ^{1}H NMR spectrum of compound **24** (300 MHz, CD_3CN). 22
Figure S32: ^{13}C NMR spectrum of compound **24** (75 MHz, CD_3CN). 22
Figure S33: ^{1}H NMR spectrum of compound **25** (300 MHz, Acetone-d_6). 23
Figure S34: ^{13}C NMR spectrum of compound **25** (75 MHz, Acetone-d_6). 23

Figure S35: ^{1}H NMR spectrum of compound **26** (300 MHz, DMSO-d_6), ... 24
Figure S36: ^{13}C NMR spectrum of compound **26** (75 MHz, DMSO-d_6). 24
Figure S37: ^{1}H NMR spectrum of compound **27** (300 MHz, DMSO-d_6), ... 25
Figure S38: ^{13}C NMR spectrum of compound **27** (75 MHz, DMSO-d_6). 25
Figure S39: ^{1}H NMR spectrum of compound **28** (300 MHz, DMSO-d_6), ... 26
Figure S40: ^{13}C NMR spectrum of compound **28** (75 MHz, DMSO-d_6). 26
Figure S41: ^{1}H NMR spectrum of compound **29** (500 MHz, DMSO-d_6), ... 27
Figure S42: ^{13}C NMR spectrum of compound **29** (126 MHz, DMSO-d_6). 27
Figure S43: ^{1}H NMR spectrum of compound **30** (300 MHz, DMSO-d_6) 28
Figure S44: ^{13}C NMR spectrum of compound **30** (75 MHz, DMSO-d_6). 28
Figure S45: ^{1}H NMR spectrum of compound **31** (300 MHz, DMSO-d_6) 29
Figure S46: ^{13}C NMR spectrum of compound **31** (75 MHz, DMSO-d_6). 29
Figure S47: ^{1}H NMR spectrum of compound **32** (300 MHz, DMSO-d_6) 30
Figure S48: ^{13}C NMR spectrum of compound **32** (75 MHz, DMSO-d_6). 30
Figure S49: Mass spectrum of compound **2.** 31
Figure S50: Mass spectrum of compound **3.** 32
Figure S51: Mass spectrum of compound **10.** 32
Figure S52: Mass spectrum of compound **11.** 33
Figure S53: Mass spectrum of compound **12.** 34
Figure S54: Mass spectrum of compound **13.** 35
Figure S55: Mass spectrum of compound **14.** 36
Figure S56: Mass spectrum of compound **15.** 37
Figure S57: Mass spectrum of compound **19** 38
Figure S58: Mass spectrum of compound **20.** 39
Figure S59: Mass spectrum of compound **21** 39
Figure S60: Mass spectrum of compound **24** 40
Figure S61: Mass spectrum of compound **25.** 40
Figure S62: Mass spectrum of compound **26.** 41
Figure S63: Mass spectrum of compound **27.** 42
Figure S64: Mass spectrum of compound **28.** 43
Figure S65: Mass spectrum of compound **29.** 44
Figure S66: Mass spectrum of compound **30.** 45
Figure S67: Mass spectrum of compound **31.** 46
Figure S68: Mass spectrum of compound **32.** 48
Figure S69: Mean values of the slopes (y values) and respective standard deviations as results of the in vitro inhibition of COX-2 (2.5 ng/µL) by *bis*-chalcone **31** (0–3.125 µM) using three substrate concentrations (x values: 6,25, 25 and 100 µM). 48
Figure S70: Sum of the squares (sum) of the different models (without inhibition, competitive inhibition, noncompetitive inhibition, uncompetitive inhibition, and mixed inhibition) determined from the results obtained from COX-2 inhibition by *bis*-chalcone **31**. 49
Figure S71: Comparison of the different models (without inhibition, competitive inhibition, noncompetitive inhibition, uncompetitive inhibition, and mixed inhibition), based on the COX-2 inhibition by *bis*-chalcone **31.** 49
Figure S72: Error parameters determination (V_{max}, K_m and K_{ic}) for competitive inhibition model of COX-2 by *bis*-chalcone **31**, through "Jackknife" procedure. 49
Figure S73: Mean values of the slopes (y values) and respective standard deviations as results of the in vitro inhibition of COX-1 (2.5 ng/µL) by *bis*-chalcone **30** (0–12.5 µM) using three substrate concentrations (x values: 6,25, 25 and 100 µM). 49
Figure S74: Sum of the squares (sum) of the different models (without inhibition, competitive inhibition, noncompetitive inhibition, uncompetitive inhibition, and mixed

inhibition) determined from the results obtained from COX-1 inhibition by *bis*-chalcone **30**. 51
Figure S75: Comparison of the different models (without inhibition, competitive inhibition, noncompetitive inhibition, uncompetitive inhibition, and mixed inhibition), based on the COX-1 inhibition by *bis*-chalcone **30.** 50
Figure S76: Error parameters determination (V_{max}, K_m and K_{ic}) for competitive inhibition model of COX-1 by *bis*-chalcone **30**, through "Jackknife" procedure. 51
Figure S77: Docking Protocol Validation for COX-1 52
Figure S78: Docking Protocol Validation for COX-2 532
Figure S79: Predicted binding poses for compounds **30** and **31** in COX-1 (PDB 6Y3C) and COX-2 (PDB 5IKT). Both proteins are overlaid, but the surface shown corresponds to COX-2. **Left)** X-ray pose of Celecoxib within COX-2 (PDB 5JW1) occupying the side pocket. **Right)** The X-ray ligand, tolfenamic acid, from the COX-2 structure used in the docking calculations. 532
Table S1: Molecular properties prediction of compound **30**.. 53
Table S2: Molecular properties prediction of compound **31**.. 54

NMR spectrum

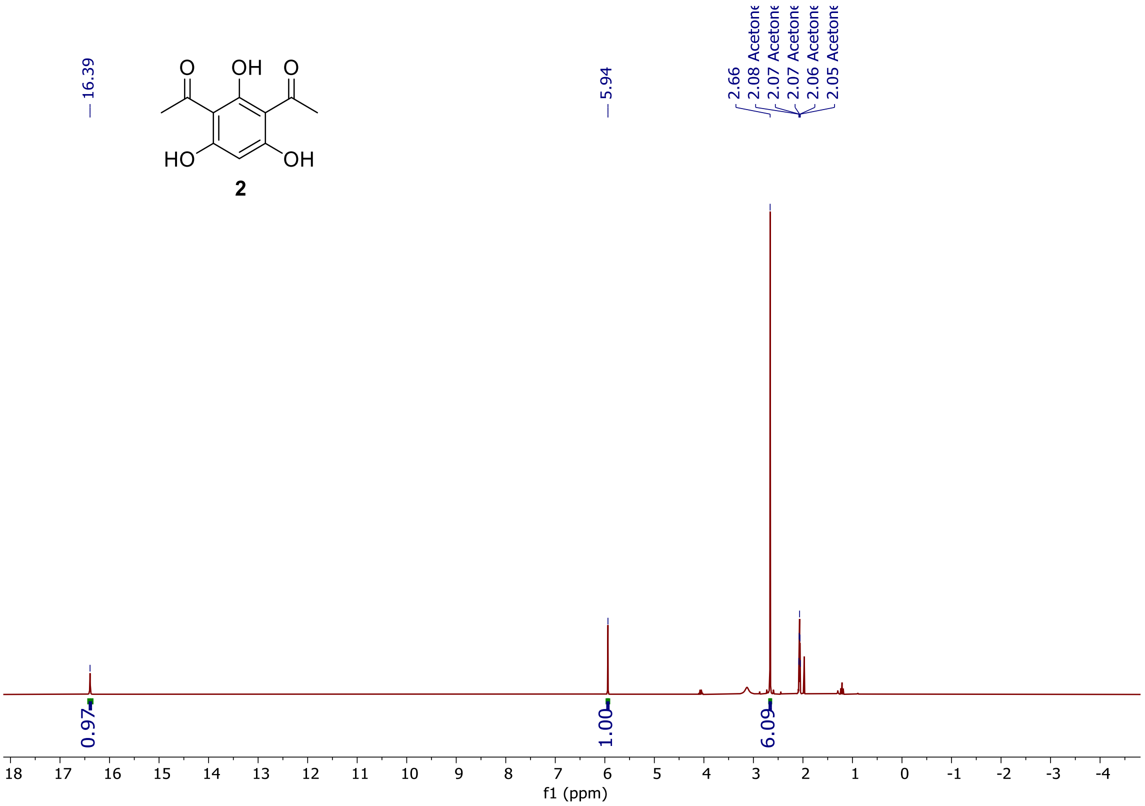

Figure S1: ^{1}H NMR spectrum of compound **2** (300 MHz, Acetone-d_6).

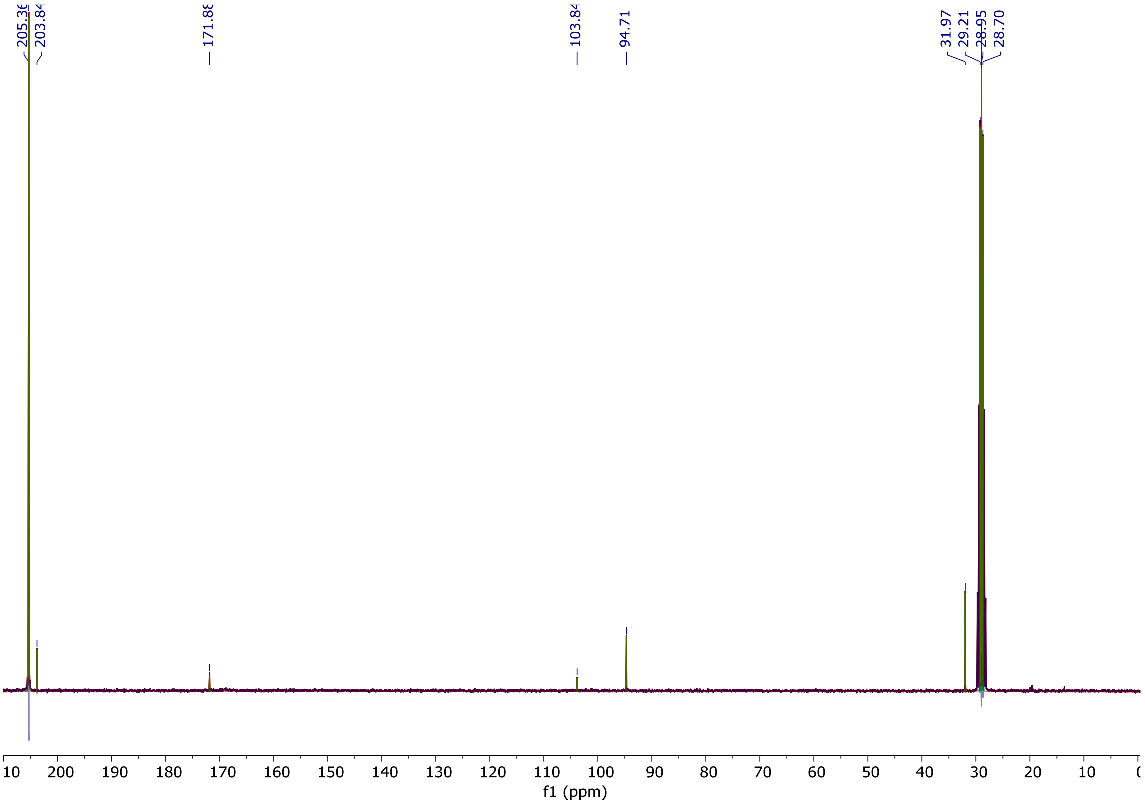

Figure S2: ^{13}C NMR spectrum of compound **2** (75 MHz, Acetone-d_6).

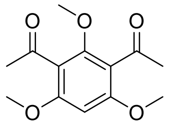

3

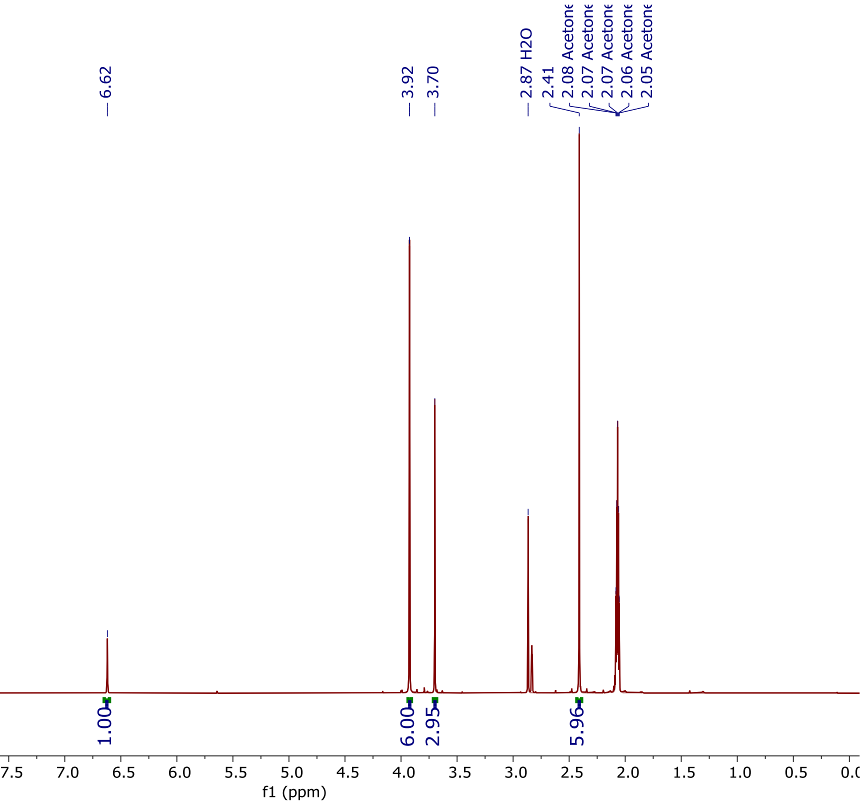

Figure S3: ^{1}H NMR spectrum of compound **3** (300 MHz, Acetone-d_6).

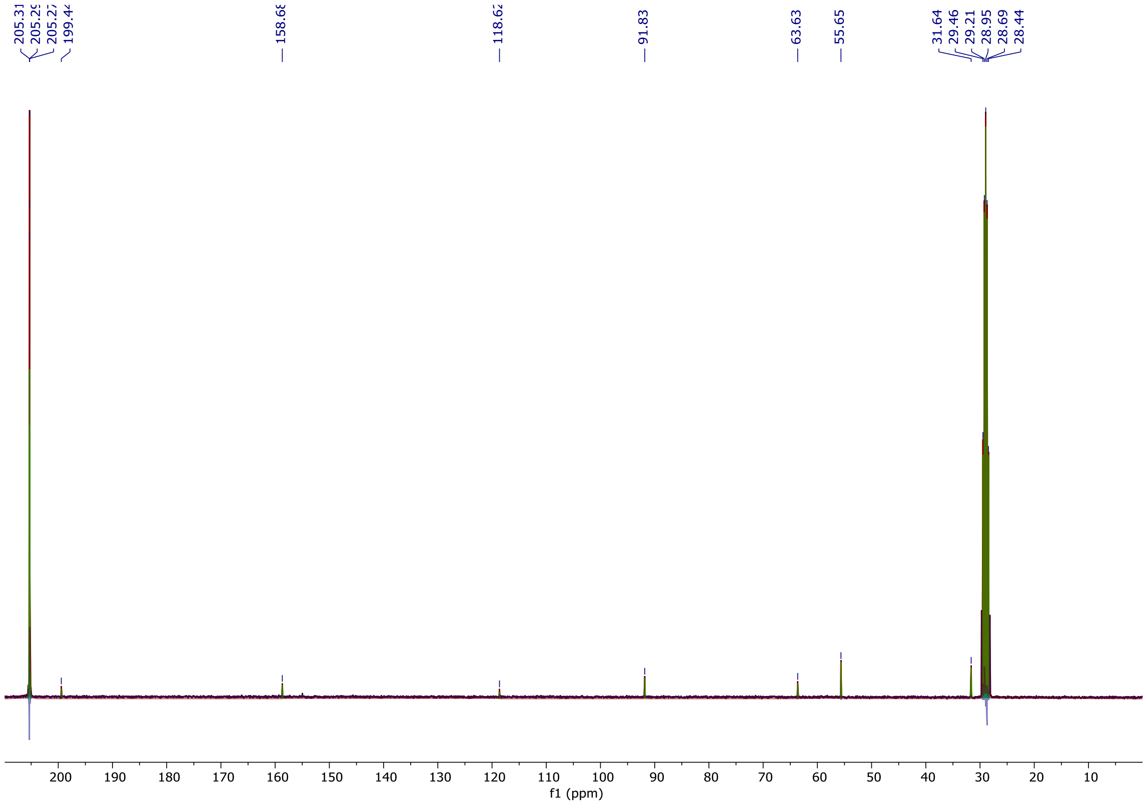

Figure S4: ^{13}C NMR spectrum of compound **3** (75 MHz, Acetone-d_6).

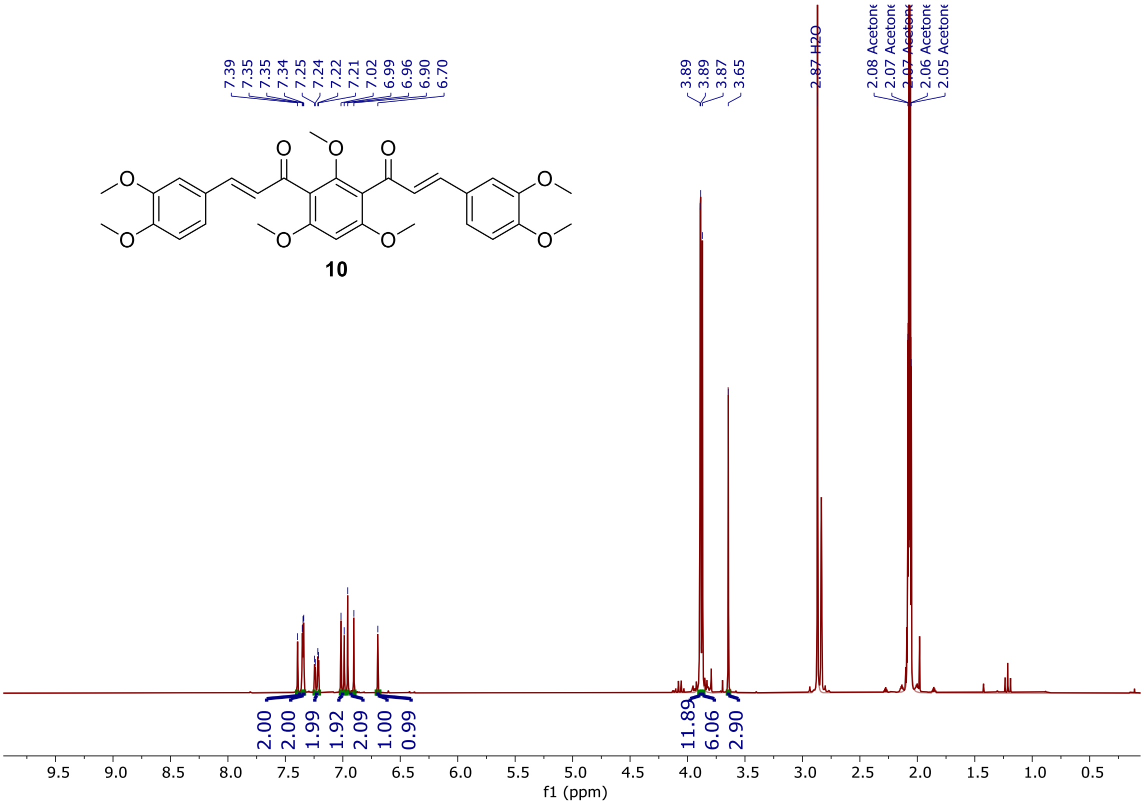

Figure S5: ^{1}H NMR spectrum of compound **10** (300 MHz, Acetone-d_6).

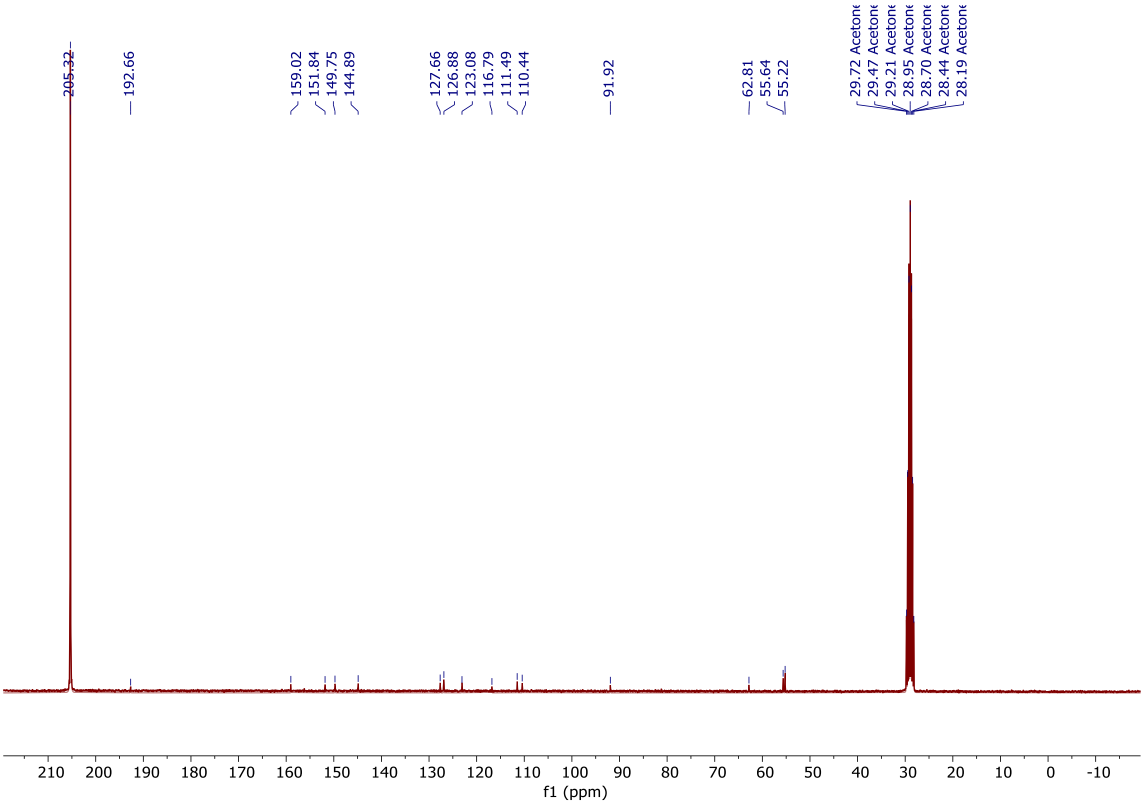

Figure S6: ^{13}C NMR spectrum of compound **10** (75 MHz, Acetone-d_6).

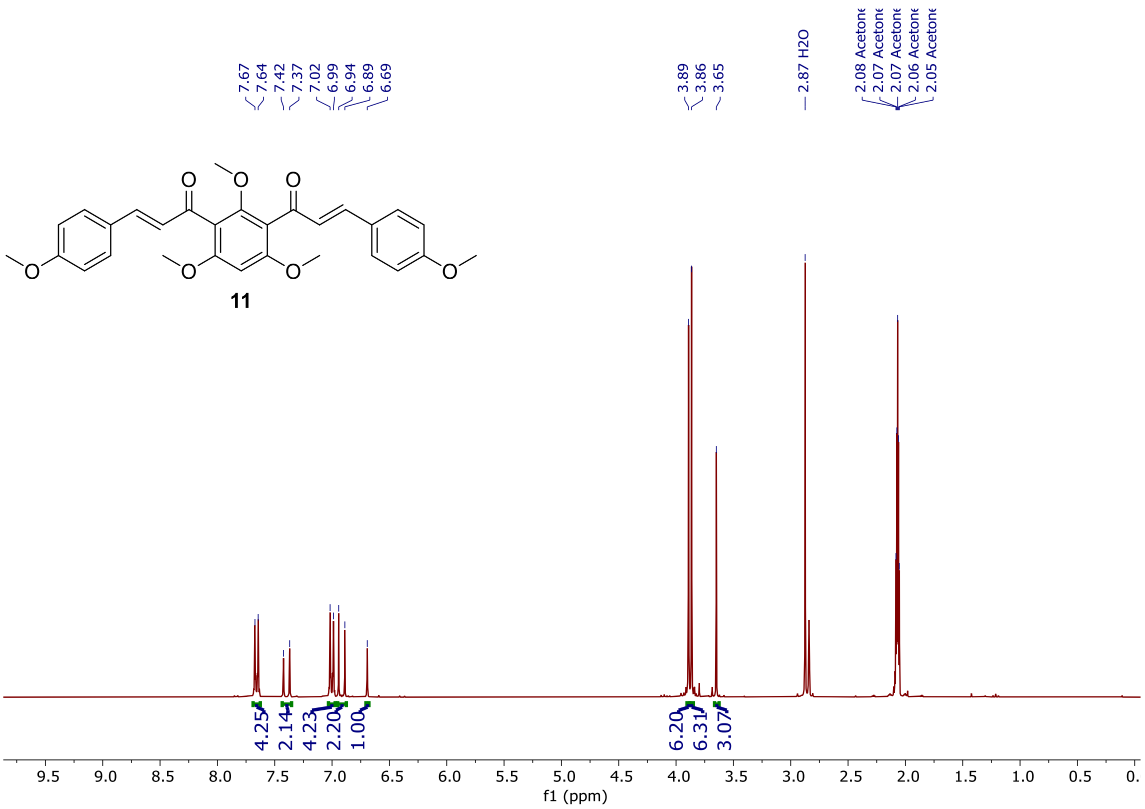

Figure S7: ^{1}H NMR spectrum of compound **11** (300 MHz, Acetone-d_6).

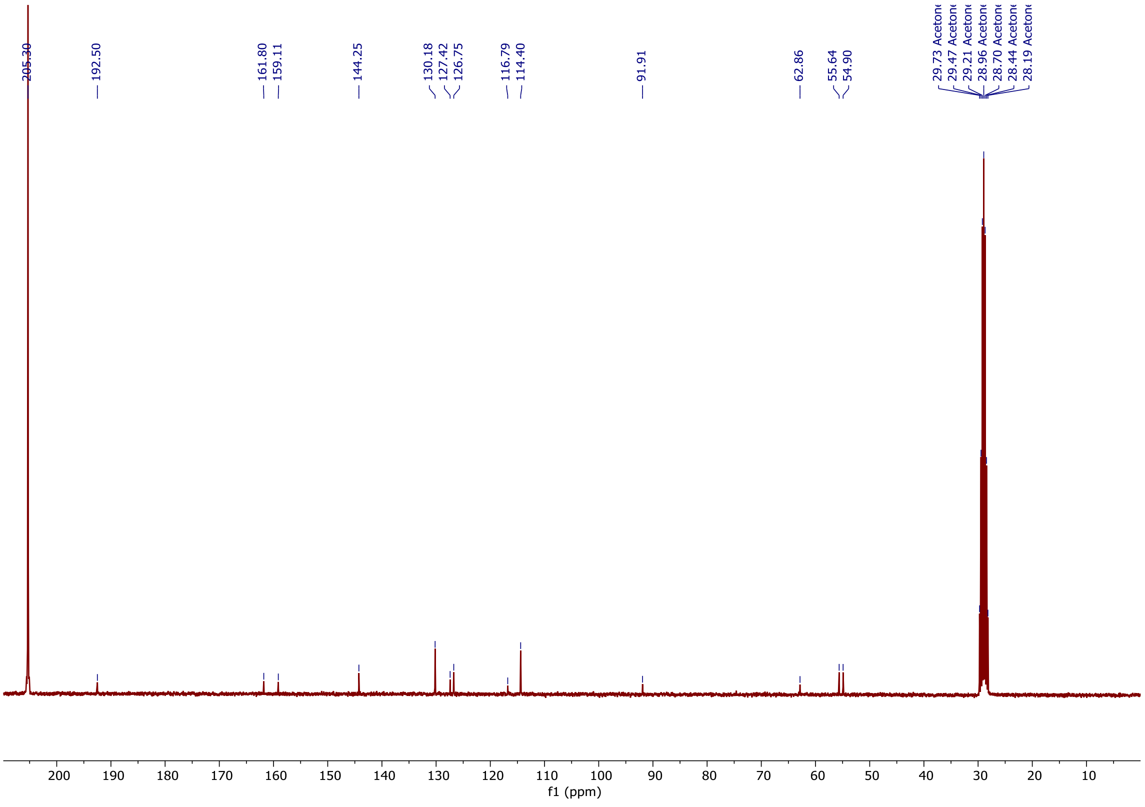

Figure S8: ^{13}C NMR spectrum of compound **11** (75 MHz, Acetone-d_6).

***(2E,2'E)*-1,1'-(2,4,6-Trimethoxy-1,3-phenylene)*bis*[3-(4-methoxyphenyl)prop-2-en-1-one]** (**11**), m.p. 175.5-176.8 ºC. **^{1}H NMR** (300 MHz, Acetone d_6) δ 7.66 (d, J = 8.8 Hz, 4H, H-2’, 6’, H-2’’, 6’’), 7.40 (d, J = 16.2 Hz, 2H, H-α, H-α’), 7.00 (d, J = 8.8 Hz, 4H, H-3’, 5’, H-3’’, 5’’), 6.92 (d, J = 16.2 Hz, 2H, H-β, H- β’), 6.69 (s, 1H, H-5), 3.89 (s, 6H, 6-OC$\underline{H}_3$, 4-OC$\underline{H}_3$), 3.86 (s, 6H, 4’-OC$\underline{H}_3$, 4’’-OC$\underline{H}_3$, 3.65 (s, 3H, 2-OC$\underline{H}_3$). **^{13}C NMR** (75 MHz, Acetone d_6) δ 192.5 (1-$\underline{C}$OCH-, 3-$\underline{C}$OCH-), 161.8 (C-4’, C-4’’), 159.1 (C-4, C-6), 156.3 (C-2), 144.3 (C-α, C-α’), 130.2 (C-2’, 6’, C-2’’, 6’’), 127.4 (C-1’,C-1’’), 126.8 (C-β, C-β’), 116.8 (C-1, C-3), 114.4 (C-3’, 5’, C-3’’, 5’’), 91.9 (C-5), 62.9 (2-O$\underline{C}$H$_3$), 55.6 (6-O$\underline{C}$H$_3$, 4-O$\underline{C}$H$_3$), 54.9 (4’-O$\underline{C}$H$_3$, 4’’-O$\underline{C}$H$_3$). **MS** (ESI$^+$) m/z (%): 489.02 $[M + H]^+$ (100). **HRMS** (ESI$^+$) m/z calcd for $C_{29}H_{29}O_7$: 489.1908 $[M + H]^+$; found: 489.1917.

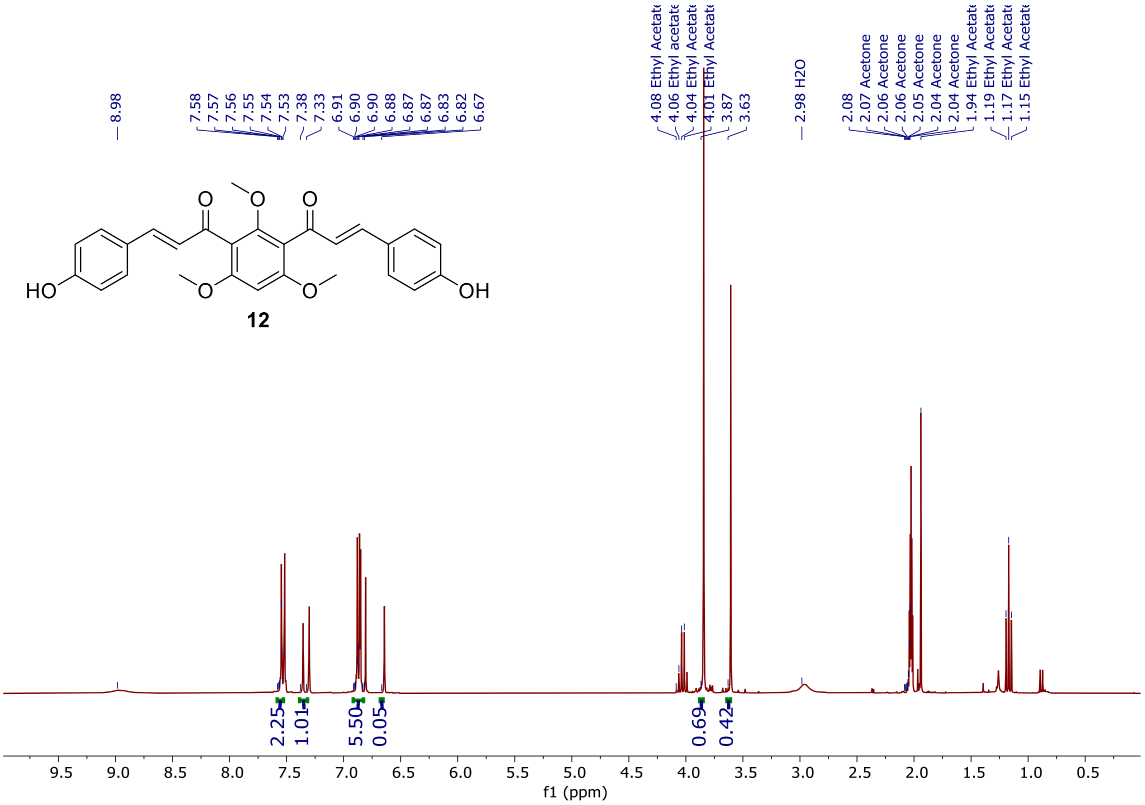

Figure S9: ^{1}H NMR spectrum of compound **12** (300 MHz, Acetone-*d6*).

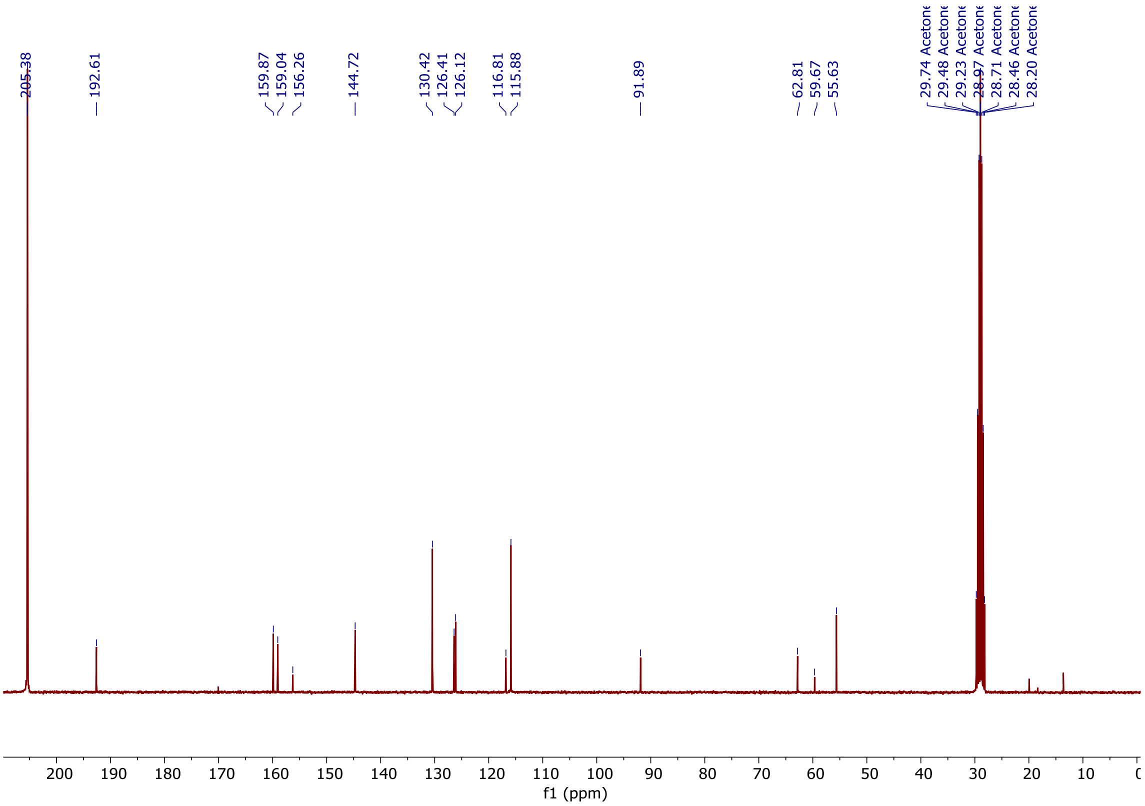

Figure S10: ^{13}C NMR spectrum of compound **12** (75 MHz, Acetone-*d6*).

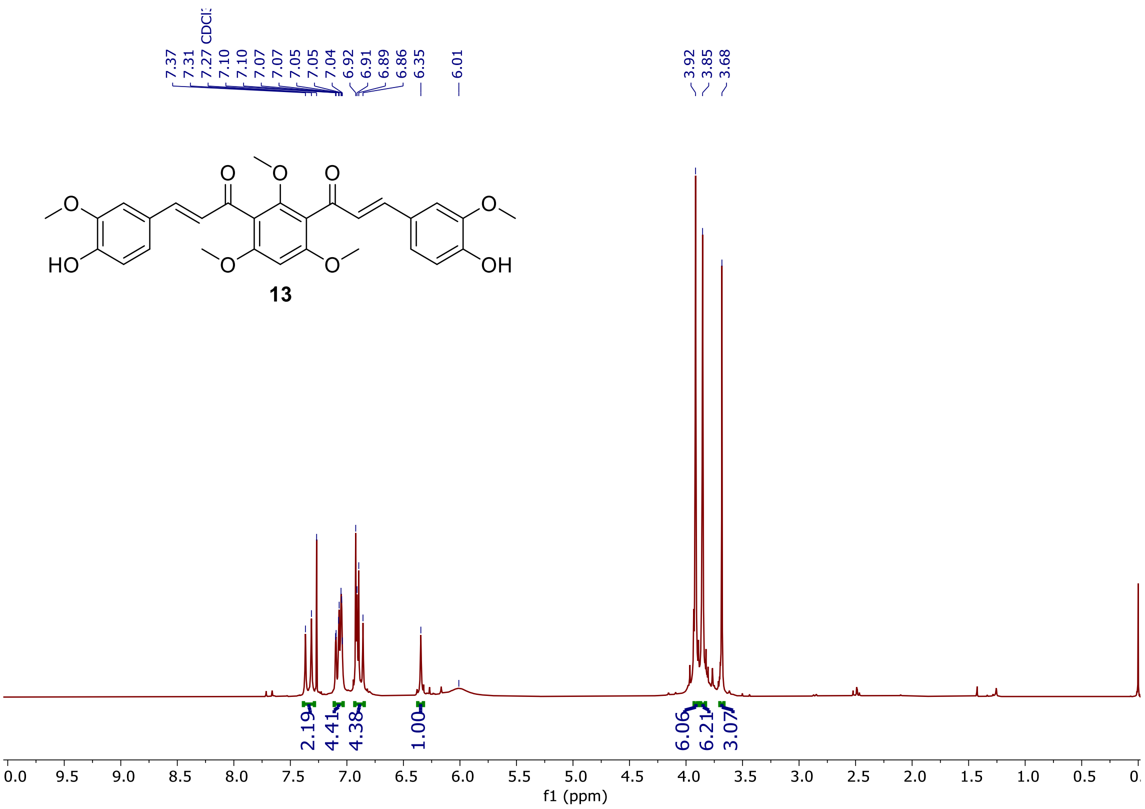

Figure S11: ^{1}H NMR spectrum of compound **13** (300 MHz, $CDCl_3$).

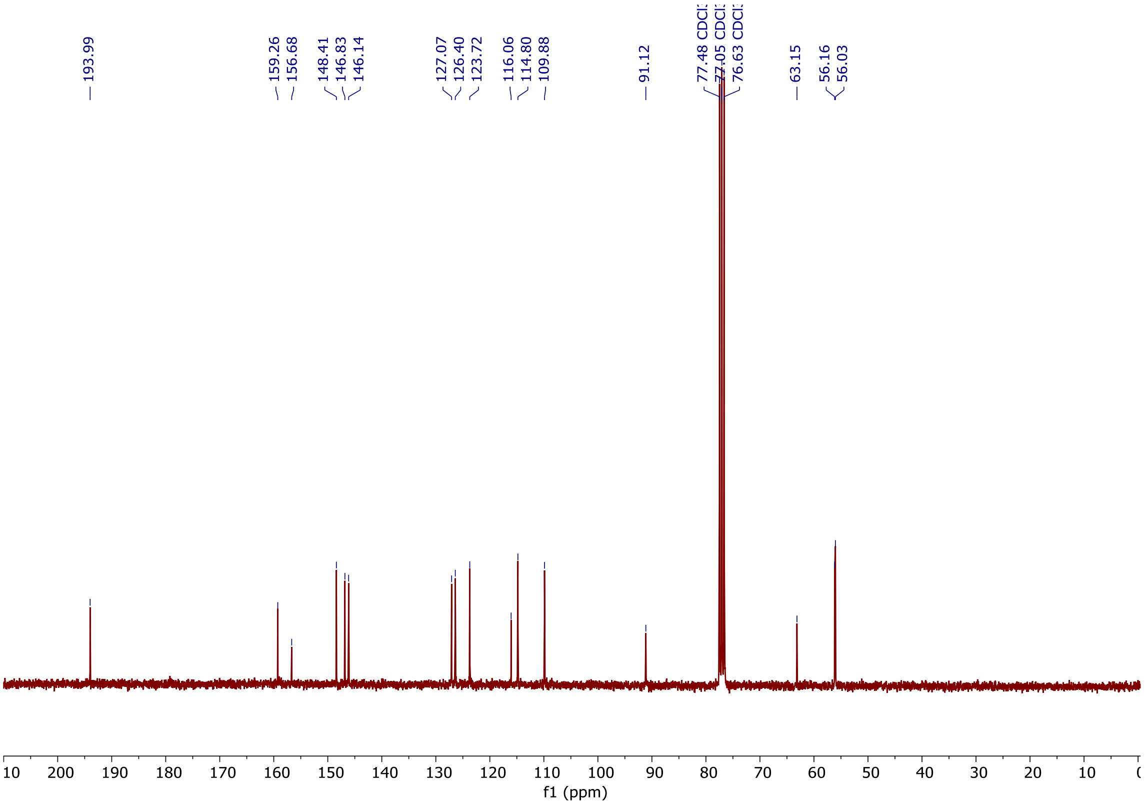

Figure S12: ^{13}C NMR spectrum of compound **13** (75 MHz, $CDCl_3$).

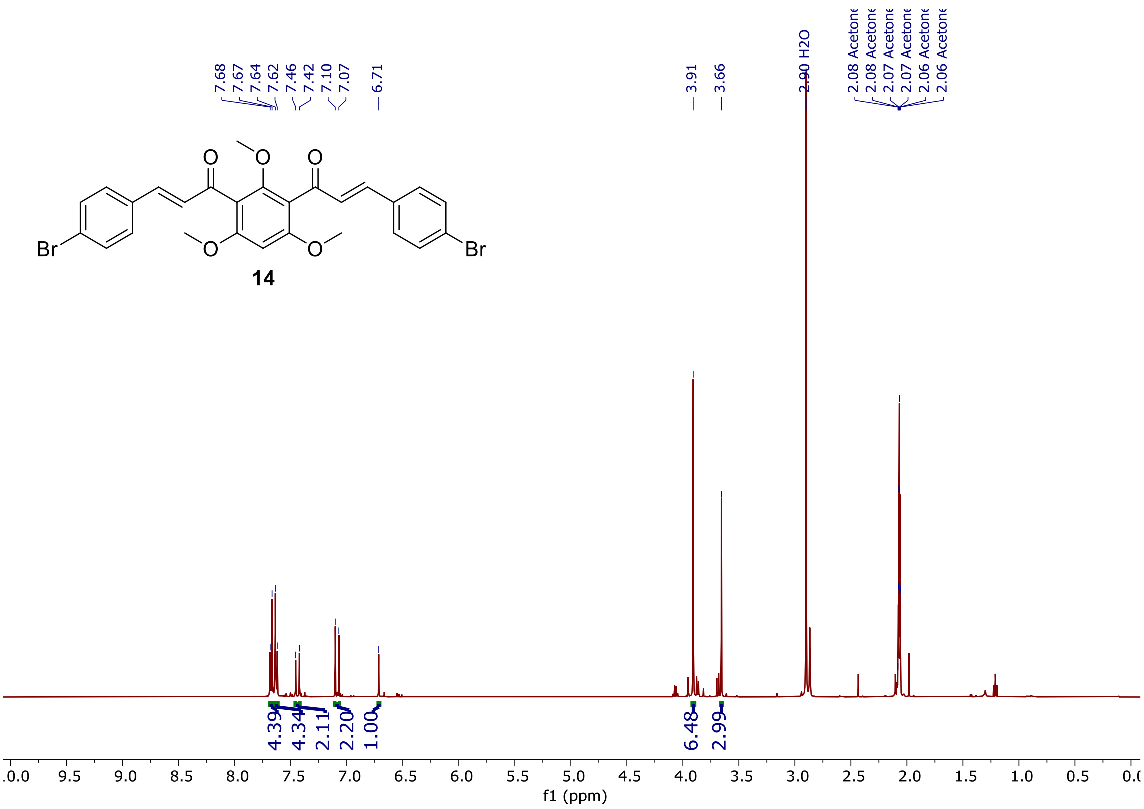

Figure S13: ^{1}H NMR spectrum of compound **14** (300 MHz, Acetone-d_6).

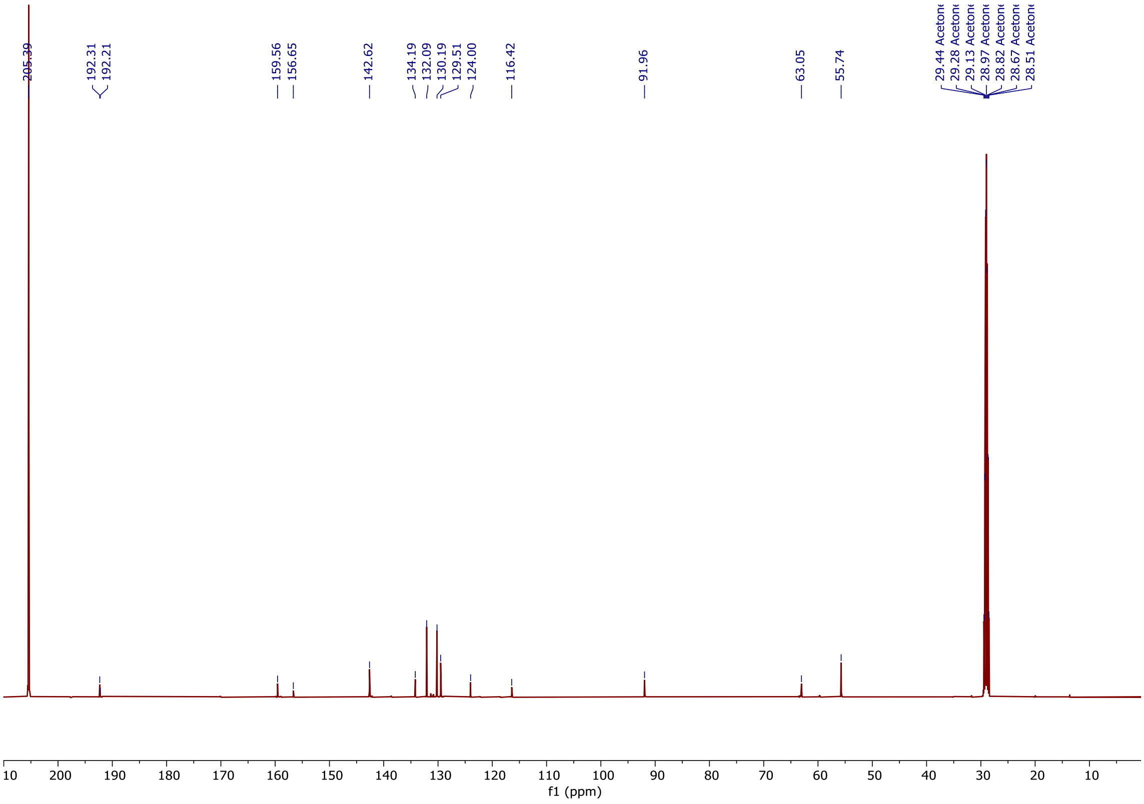

Figure S14: ^{13}C NMR spectrum of compound **14** (75 MHz, Acetone-d_6).

(*2E,2'E*)-1,1'-(2,4,6-Trimethoxy-1,3-phenylene)*bis*[3-(4-bromophenyl)prop-2-en-1-one] (**14**) light yellow solid, 67 % yield, m.p. 192.9-193.9 ºC. **^{1}H NMR** (500 MHz, Acetone-d_6) *δ* 7.68 (d, *J* = 8.6 Hz, 4H, H-2', 6', H-2'', 6''), 7.63 (d, *J* = 8.6 Hz, 4H, H-3', 5', H-3'', 5''), 7.44 (d, *J* = 16.2 Hz, 2H, H-β, H-β'), 7.09 (d, *J* = 16.2 Hz, 2H, H-α, H-α'), 6.71 (s, 1H, H-5), 3.91 (s, 6H, 6-OC$\underline{H}_3$, 4-OC$\underline{H}_3$), 3.66 (s, 3H, 2-OC$\underline{H}_3$). **^{13}C NMR** (126 MHz, Acetone-d_6) δ 192.3 (1-$\underline{C}$OCH-, 3-$\underline{C}$OCH-), 159.6 (C4, C6), 156.7 (C-2), 142.6 (C-α, C-α'), 134.2 (C-4', C-4'') 132.1 (C-3', 5', C-3'', 5''), 130.2 (C-2', 6', C-2'', 6''), 129.5 (C-β, C-β') 124.0 (C-1',C-1''), 116.4 (C-1, C-3), 91.9 (C-5), 63.1 (2-O$\underline{C}H_3$), 55.7 (6-O$\underline{C}H_3$, 4-O$\underline{C}H_3$). **MS** (ESI$^+$) m/z (%): 587.0 [M + H]$^+$ (^{79}Br, 100), 589.0 [M + H]$^+$ (^{81}Br, 50). **HRMS** (ESI$^+$) m/z calcd for $C_{27}H_{23}Br_2O_5$: 586.9887 [M + H, ^{79}Br]$^+$; found: 586.9867.

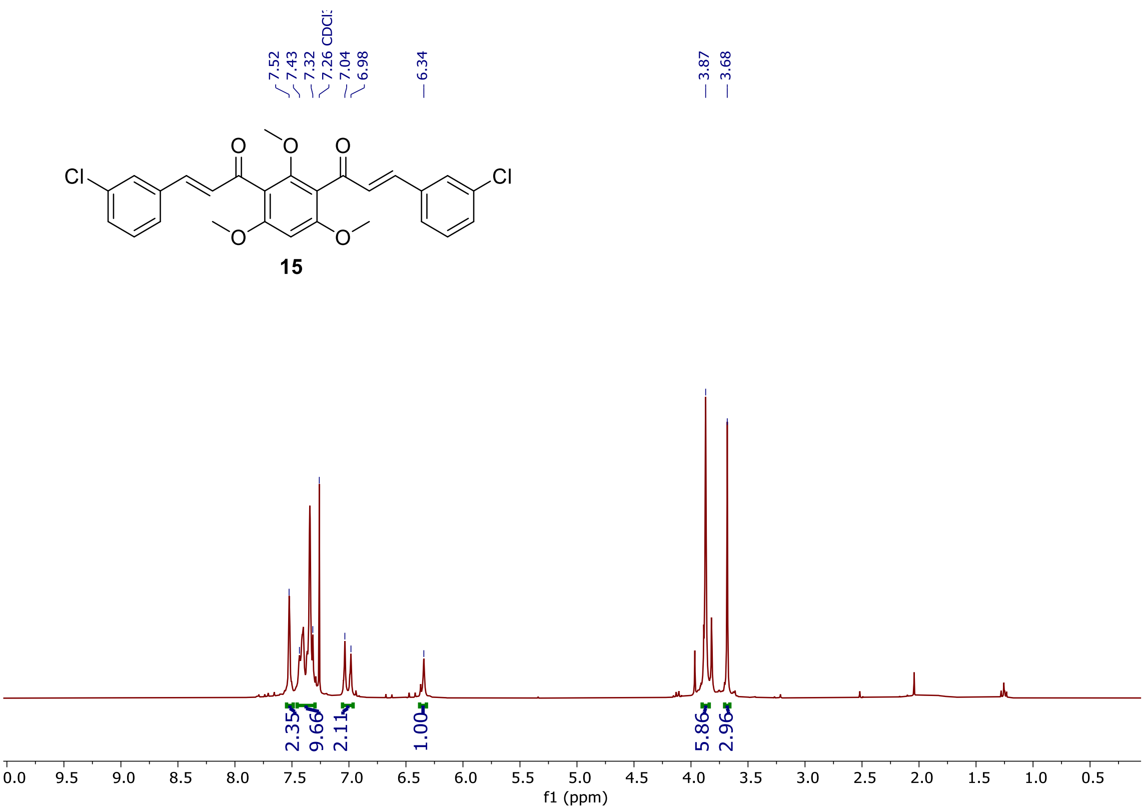

Figure S15: ^{1}H NMR spectrum of compound **15** (300 MHz, $CDCl_3$).

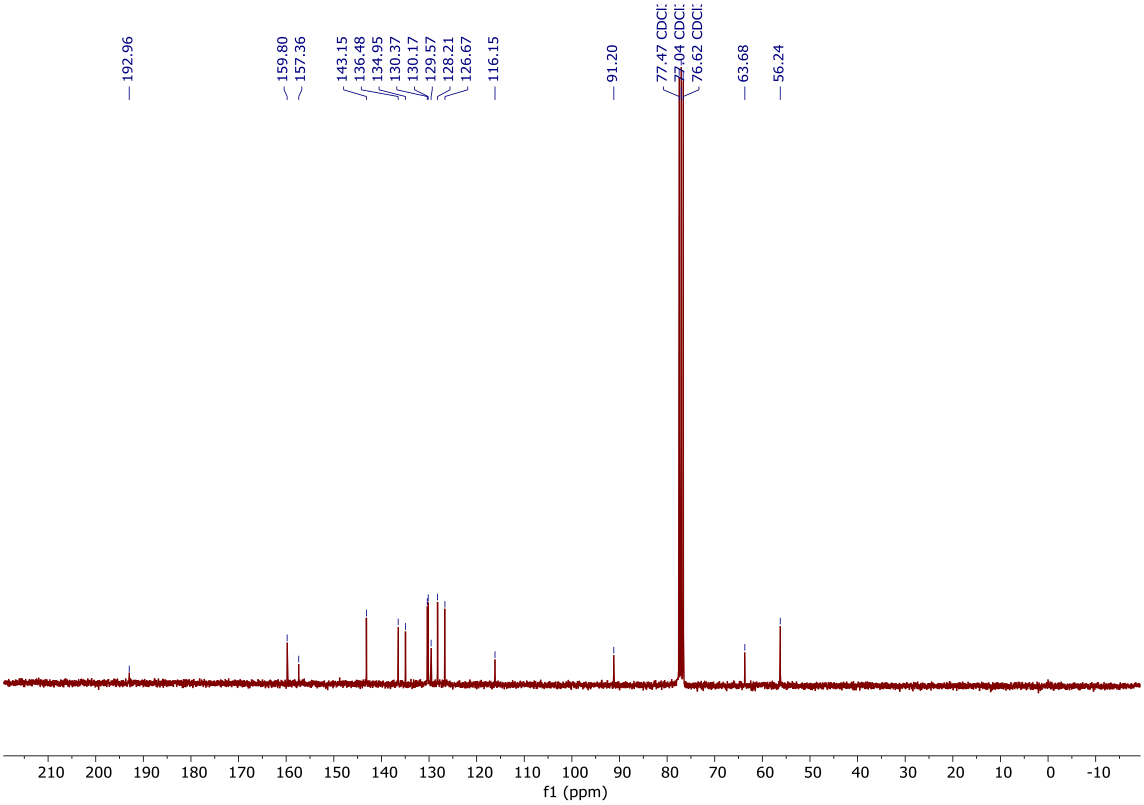

Figure S16: ^{13}C NMR spectrum of compound **15** (75 MHz, $CDCl_3$).

(*2E,2'E*)-1,1'-(2,4,6-Trimethoxy-1,3-phenylene)*bis*[3-(3-chlorophenyl)prop-2-en-1-one] (**15**) light yellow solid, 60 % yield, m.p. 144.3-145.2 ºC. **^{1}H NMR** (300 MHz, $CDCl_3$) δ 7.53 (d, J = 1.8 Hz, 2H, H-2', H-2''), 7.42 (m, 3H,), 7.38 – 7.30 (m, 5H), 7.01 (d, J = 16.0 Hz, 2H, H-α, H-α'), 6.35 (s, 1H, H-5), 3.88 (s, 6H, 4-OC$\underline{H}_3$, 6-OC$\underline{H}_3$), 3.69 (s, 3H, 2-OC$\underline{H}_3$).**^{13}C NMR** (75 MHz, $CDCl_3$) δ 192.8 (1-$\underline{C}$OCH-, 3-$\underline{C}$OCH-), 159.8 (C4, C6), 157.4 (C-2), 143.2 (C-β, C-β'), 136.5 (C-2), 135.0 (C-3', C-3''), 130.4 (C-4', C-4''), 130.2 (C-6', C-6''), 129.6 (C-α, C-α'), 128.2 (C-2', C-2''), 126.7 (C-5', C-5''), 116.2 (C-1, C-3), 91.2 (C-5), 63.7 (2-O$\underline{C}H_3$), 56.2 (6-O$\underline{C}H_3$, 4-O$\underline{C}H_3$). **S** (ESI$^+$) m/z (%): 497.1 $[M + H]^+$ (^{35}Cl, 95), 499.1 $[M + H]^+$ (^{37}Cl, 50). **HRMS** (ESI$^+$) m/z calcd for $C_{27}H_{23}Cl_2O_5$: 497.0918 ($[M+H]^+$,^{35}Cl) ; found: 497.0900.

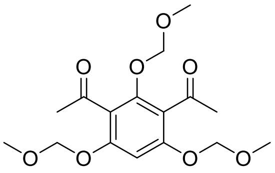

16a

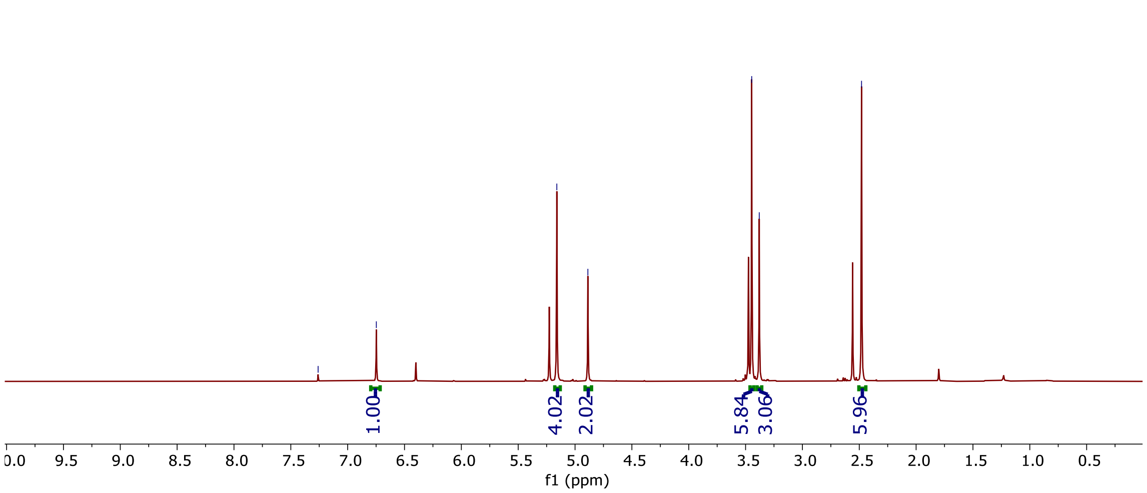

Figure S17: ^{1}H NMR spectrum of compound **16a** (300 MHz, $CDCl_3$).

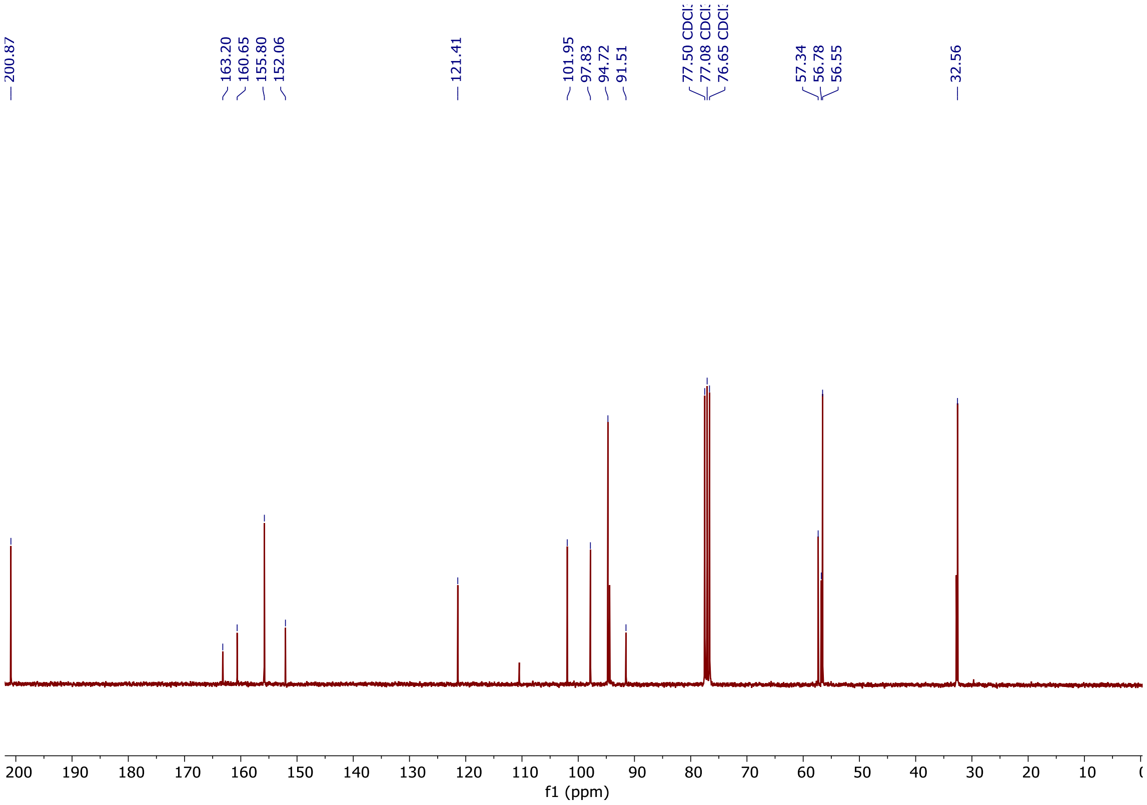

Figure S18: ^{13}C NMR spectrum of compound **16** (75 MHz, $CDCl_3$).

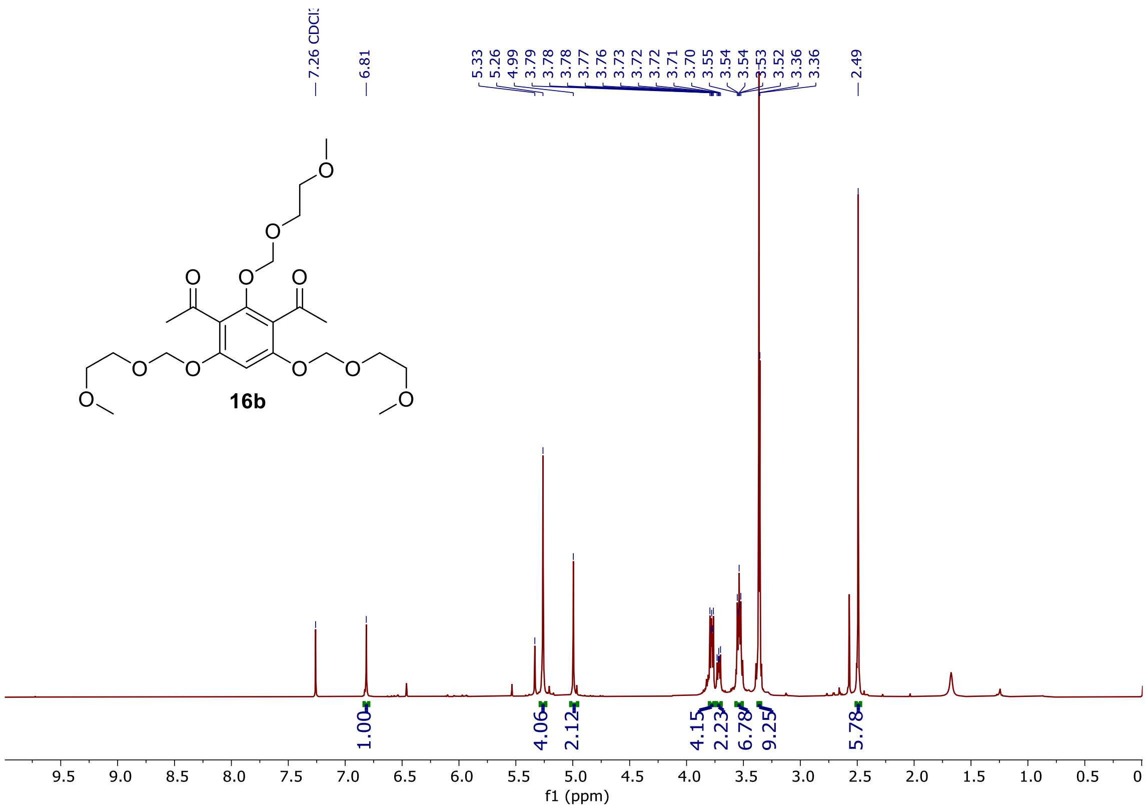

Figure S19: ^{1}H NMR spectrum of compound **16b** (300 MHz, $CDCl_3$).

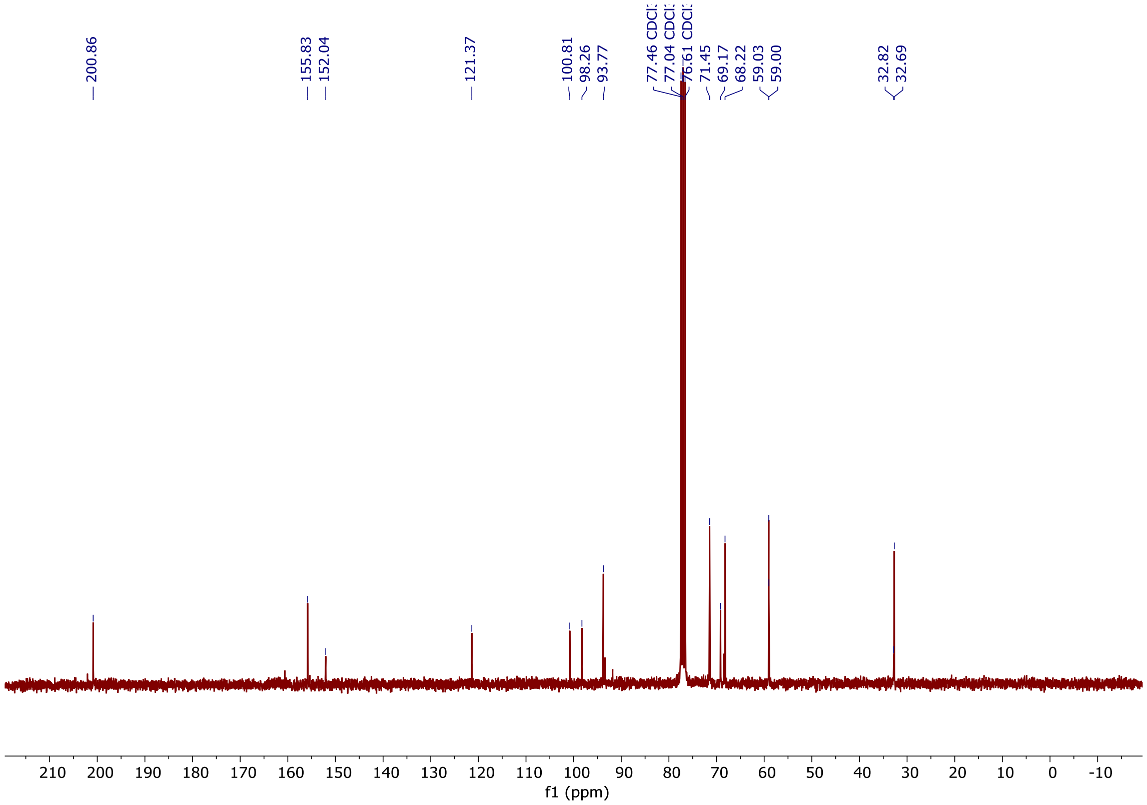

Figure S20: ^{13}C NMR spectrum of compound **16b** (75 MHz, $CDCl_3$).

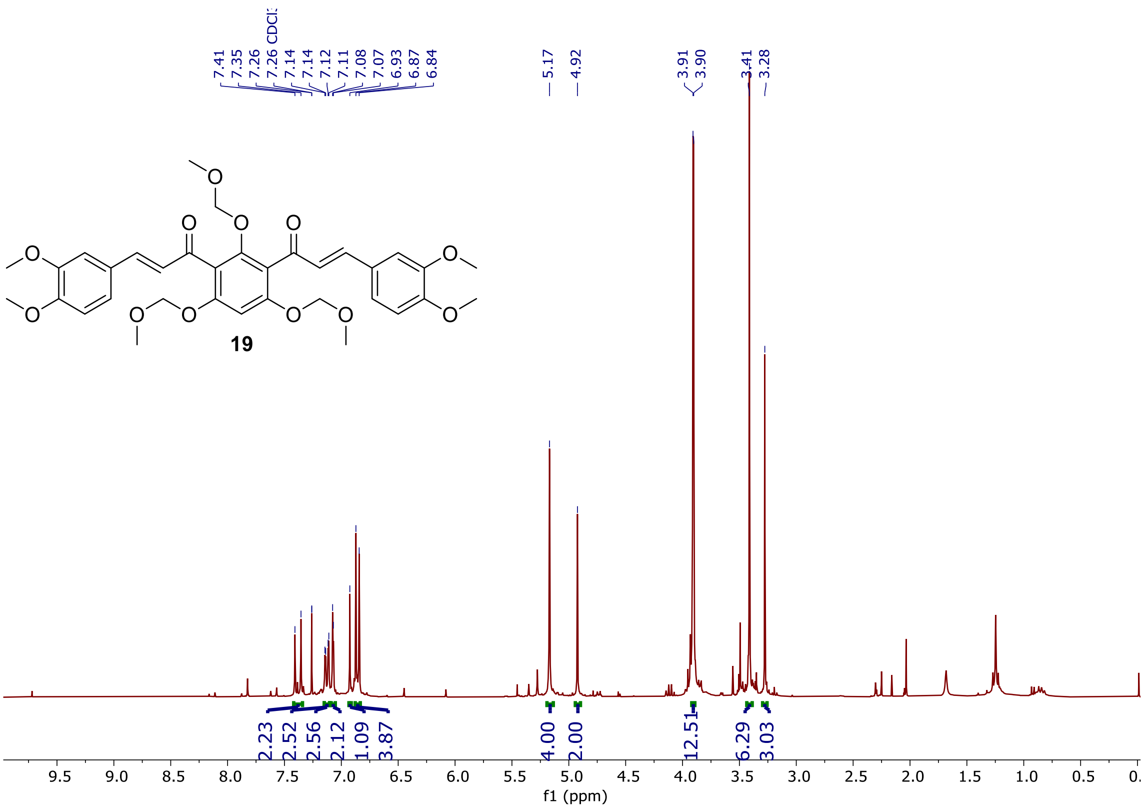

Figure S21: ^{1}H NMR spectrum of compound **19** (300 MHz, $CDCl_3$).

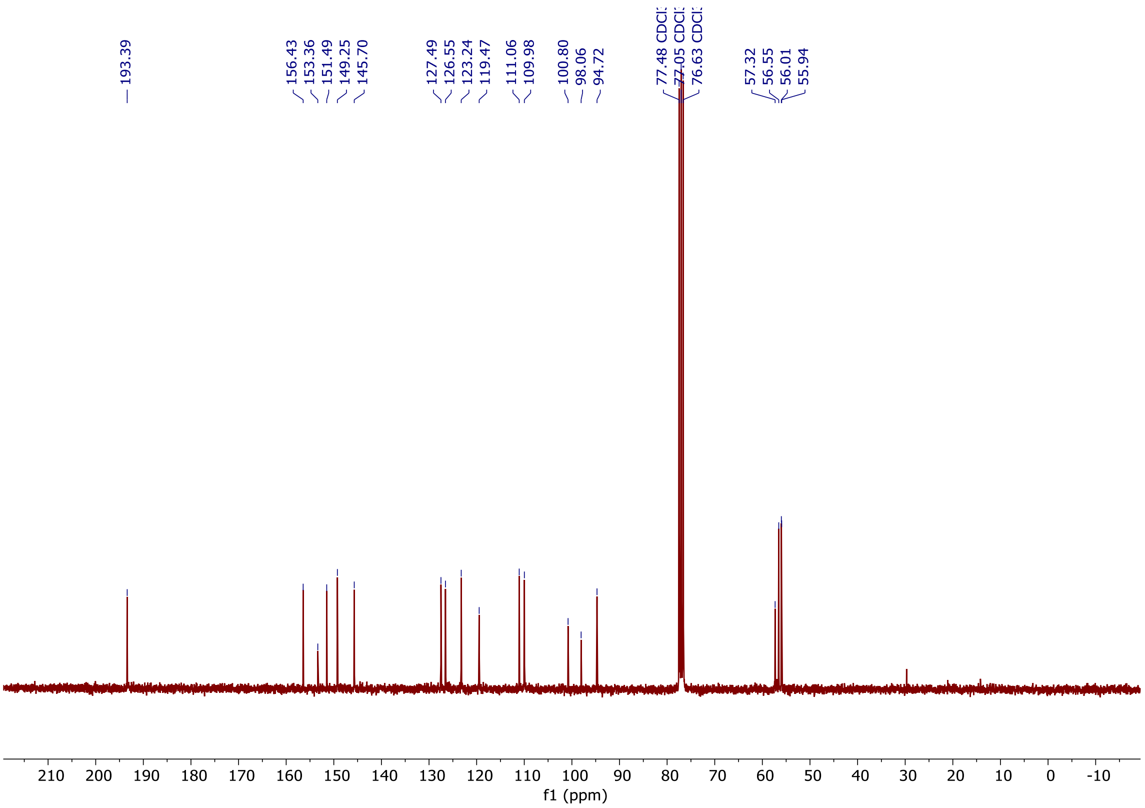

Figure S22: ^{13}C NMR spectrum of compound **19** (75 MHz, $CDCl_3$).

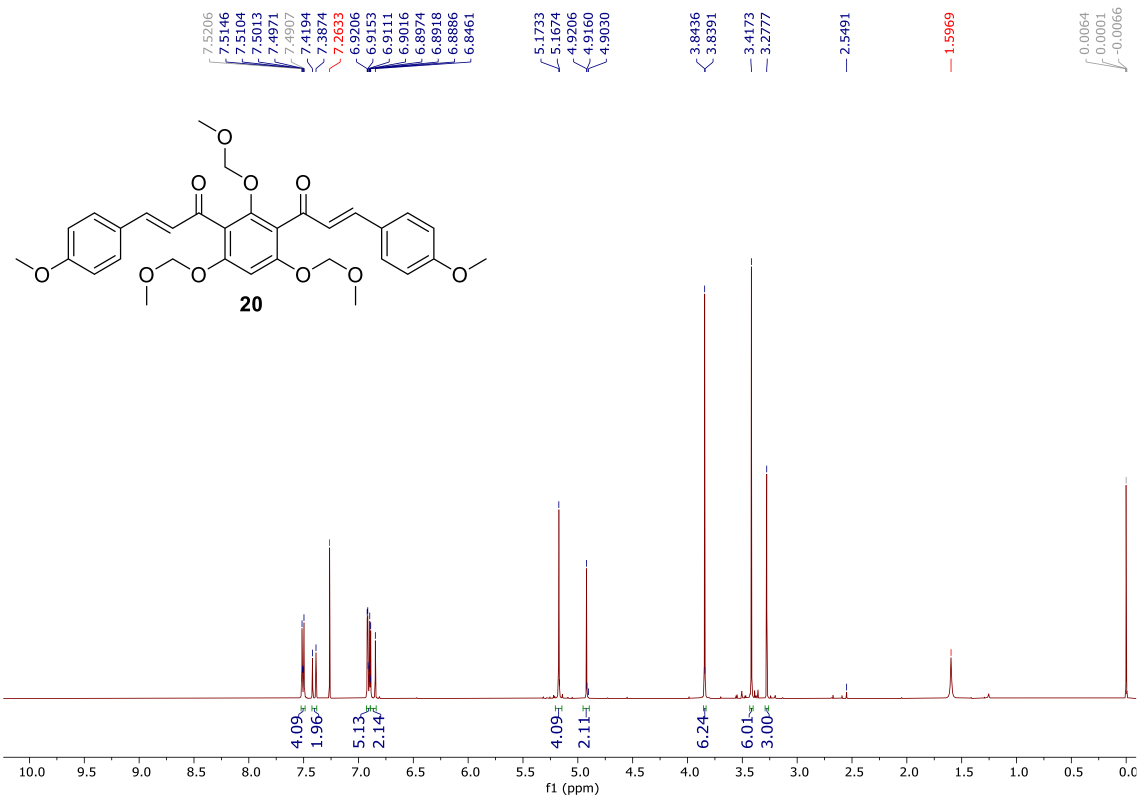

Figure S23: ^{1}H NMR spectrum of compound **20** (500 MHz, $CDCl_3$).

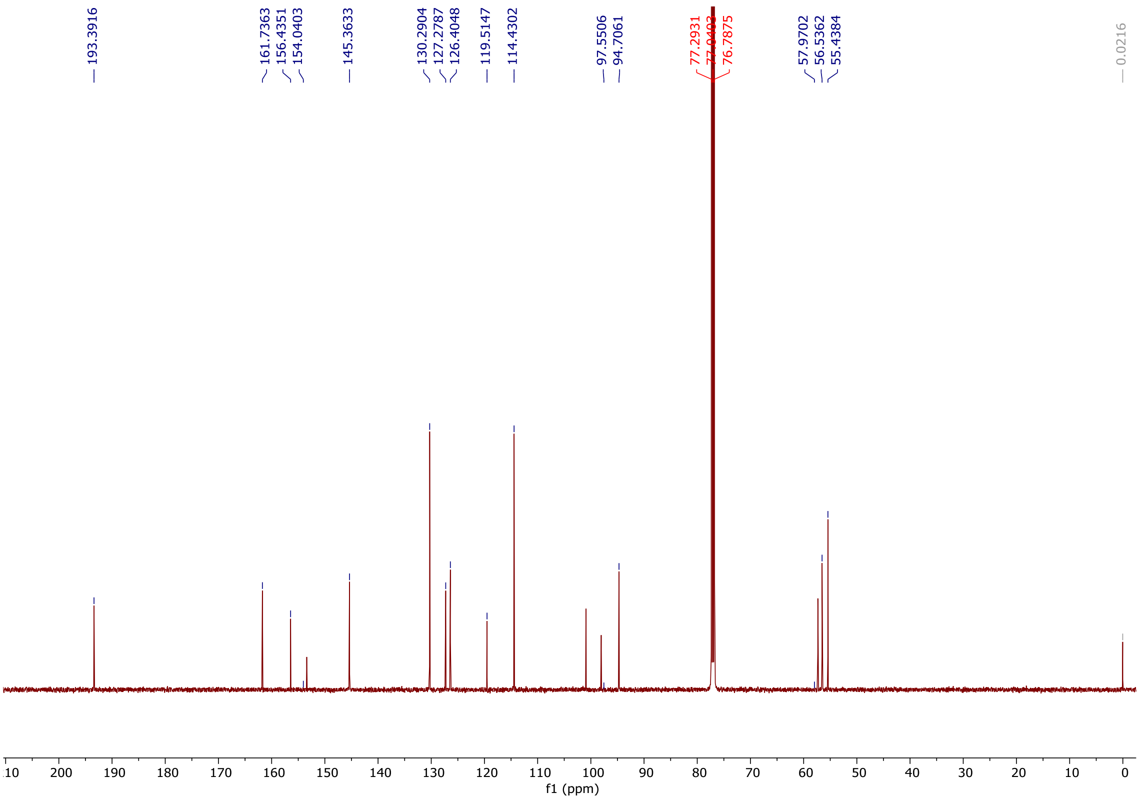

Figure S24: ^{13}C NMR spectrum of compound **20** (126 MHz, $CDCl_3$).

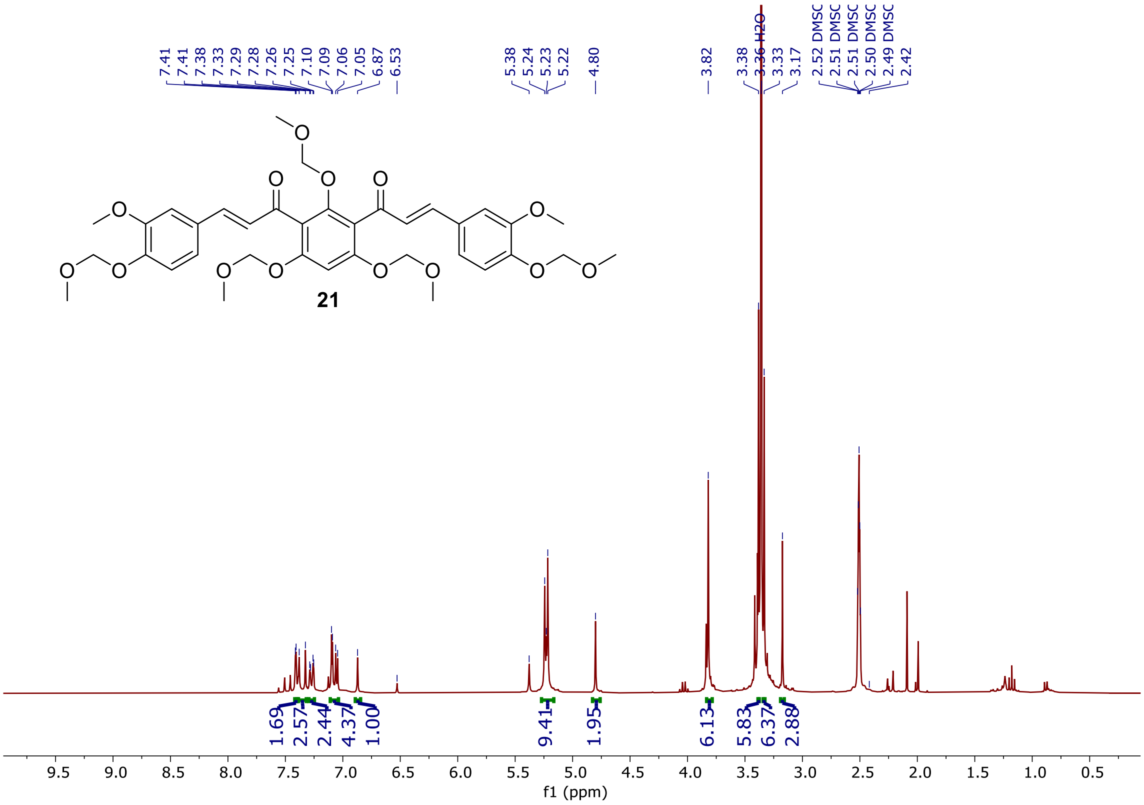

Figure S25: ^{1}H NMR spectrum of compound **21** (300 MHz, Acetone-d_6).

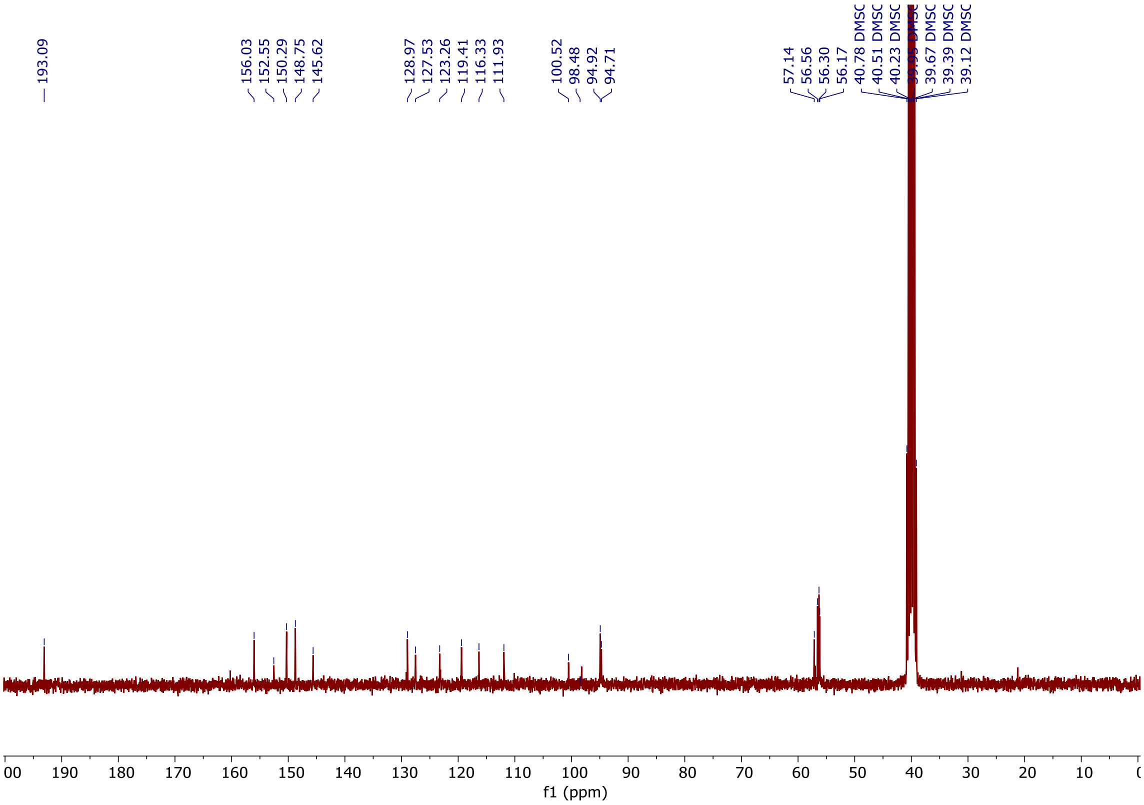

Figure S26: ^{13}C NMR spectrum of compound **21** (75 MHz, Acetone-d_6).

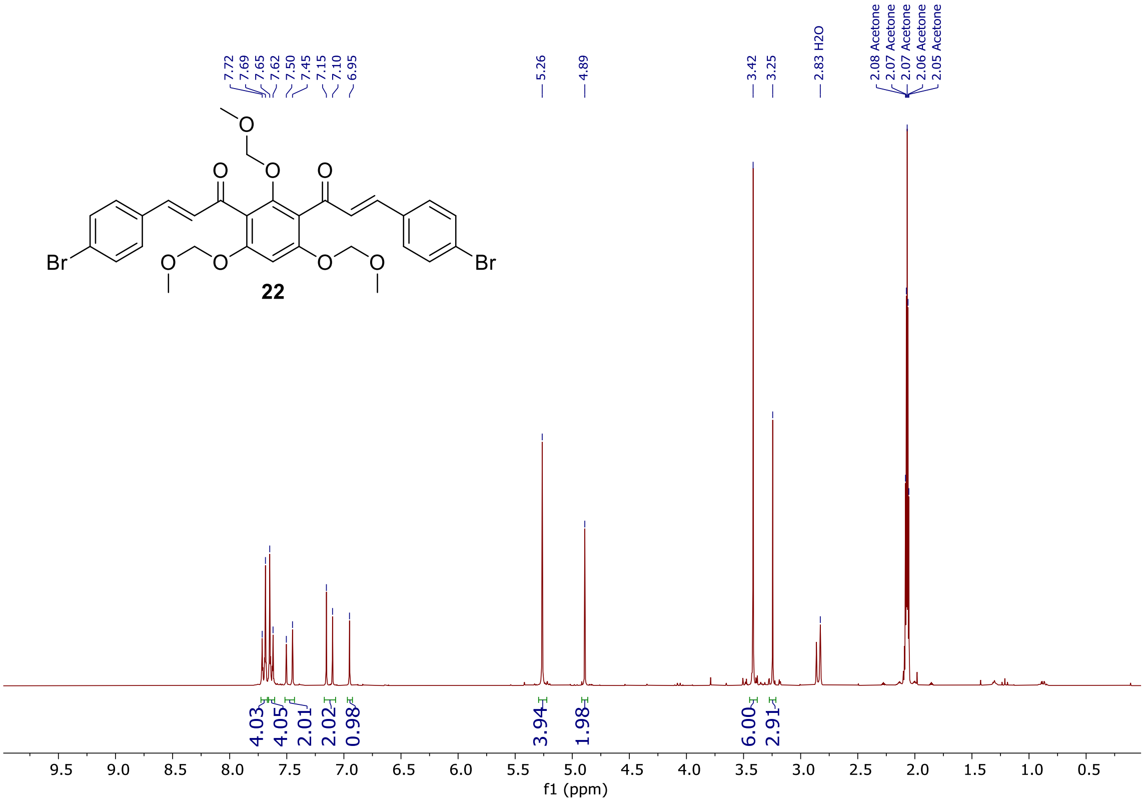

Figure S27: ^{1}H NMR spectrum of compound **22** (300 MHz, Acetone-d_6).

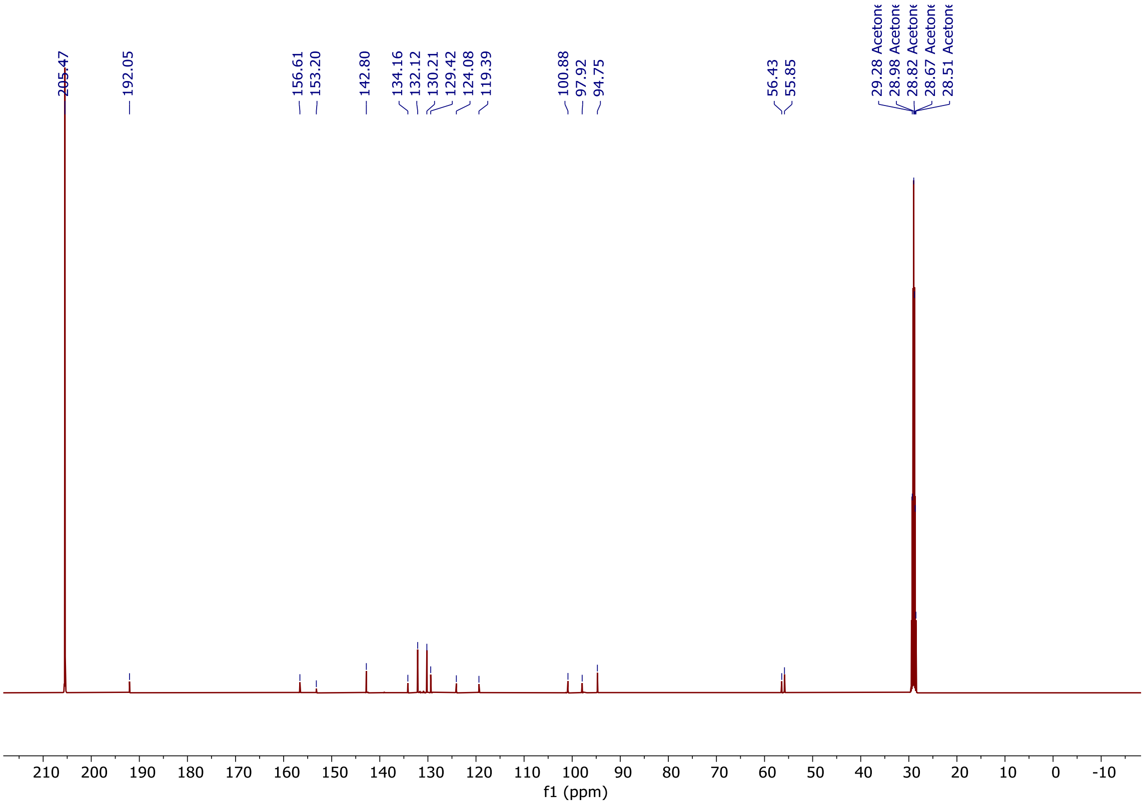

Figure S28: ^{13}C NMR spectrum of compound **22** (75 MHz, Acetone-d_6).

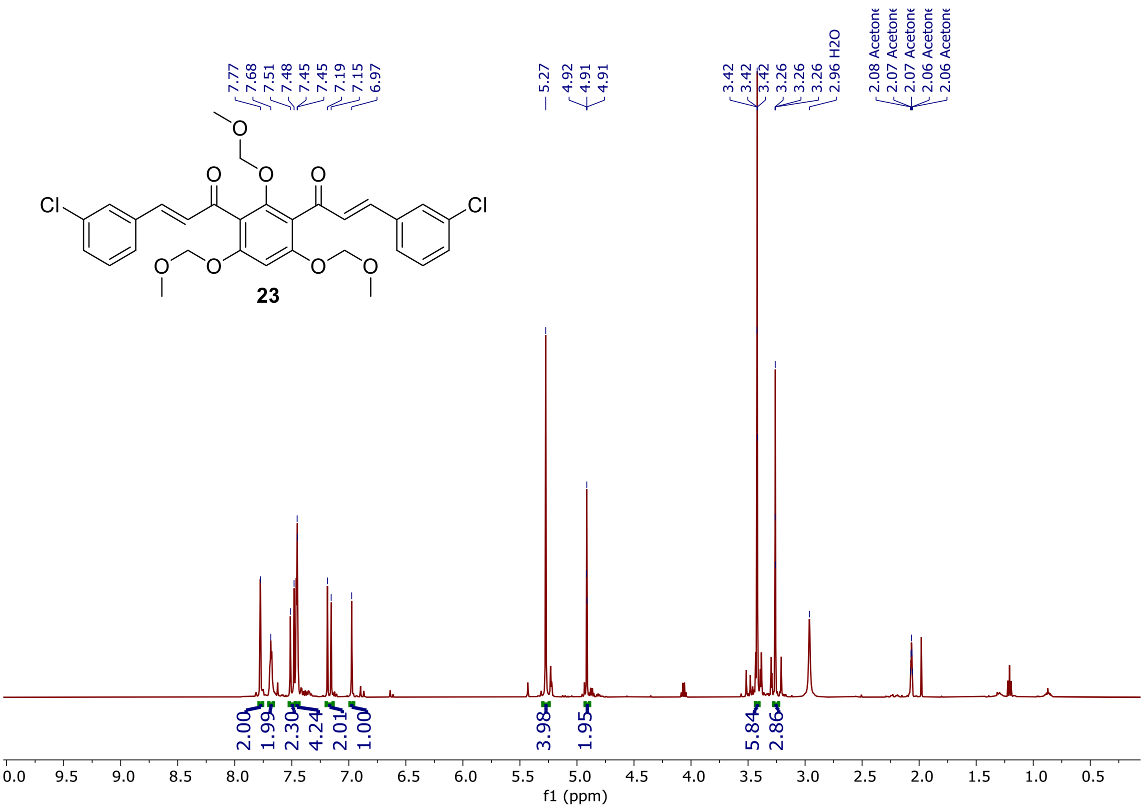

Figure S29: ^{1}H NMR spectrum of compound **23** (300 MHz, Acetone-d_6).

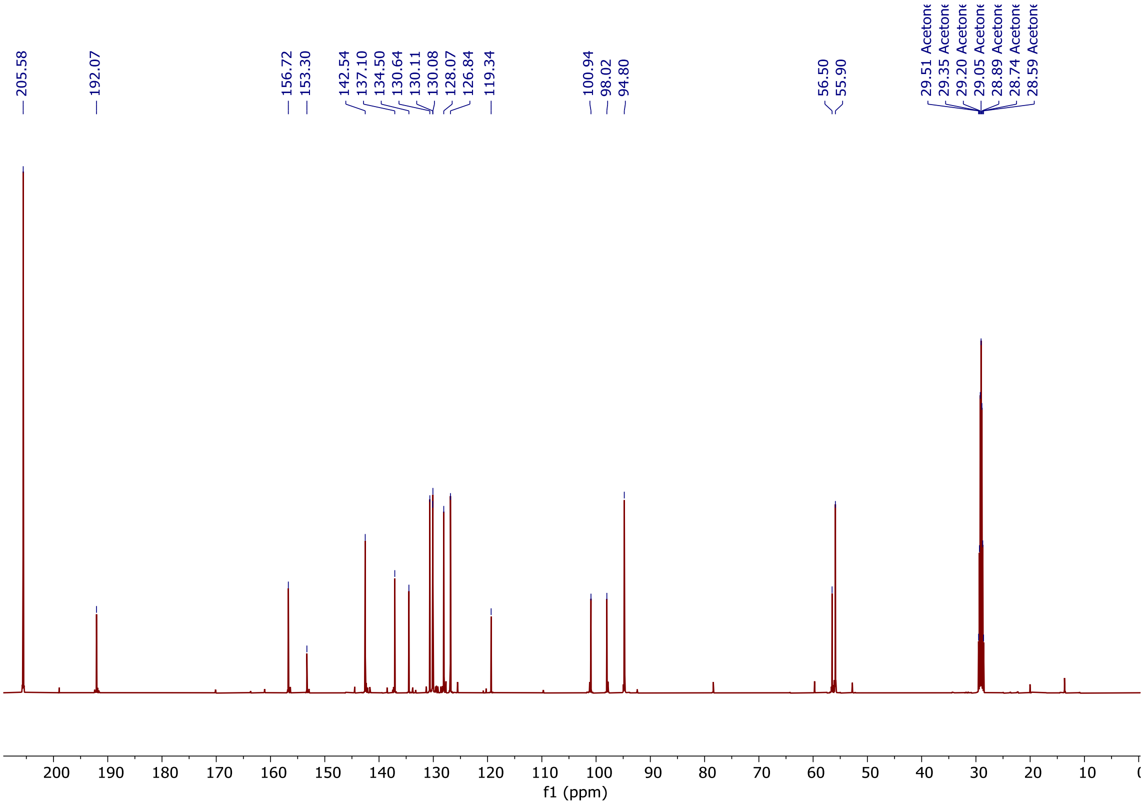

Figure S30: ^{13}C NMR spectrum of compound **23** (75 MHz, Acetone-d_6).

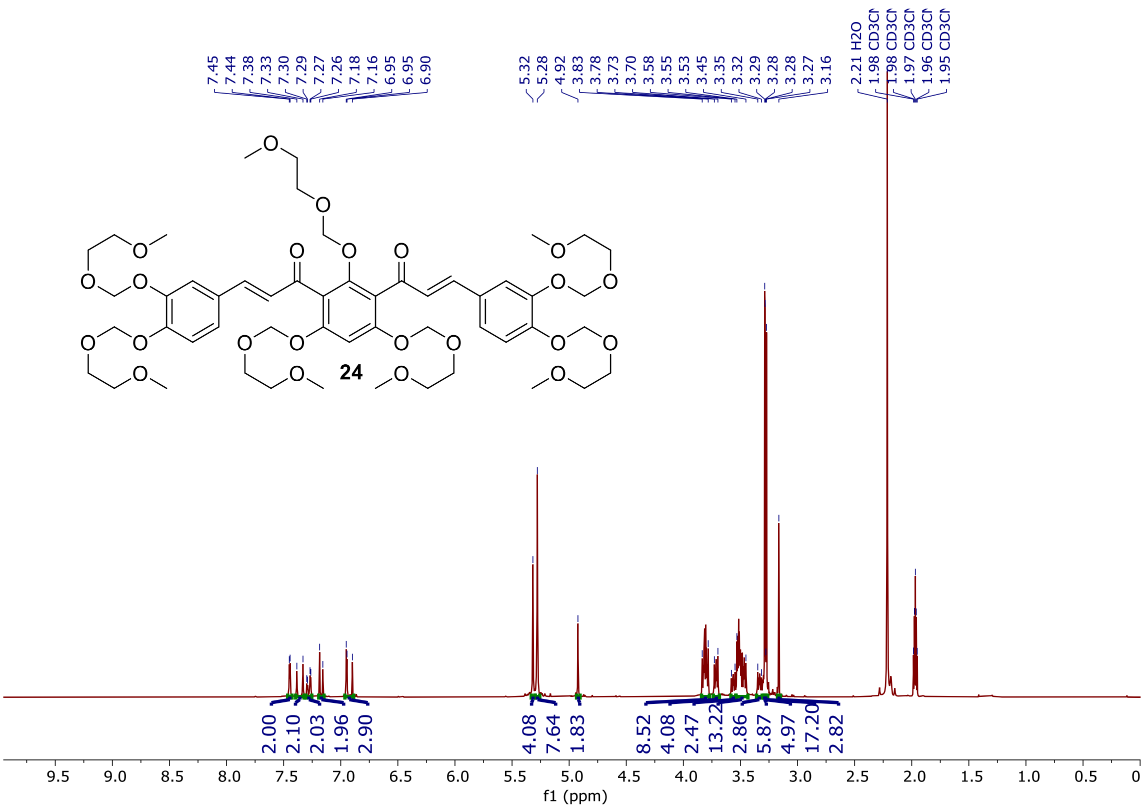

Figure S31: ^{1}H NMR spectrum of compound **24** (300 MHz, CD_3CN).

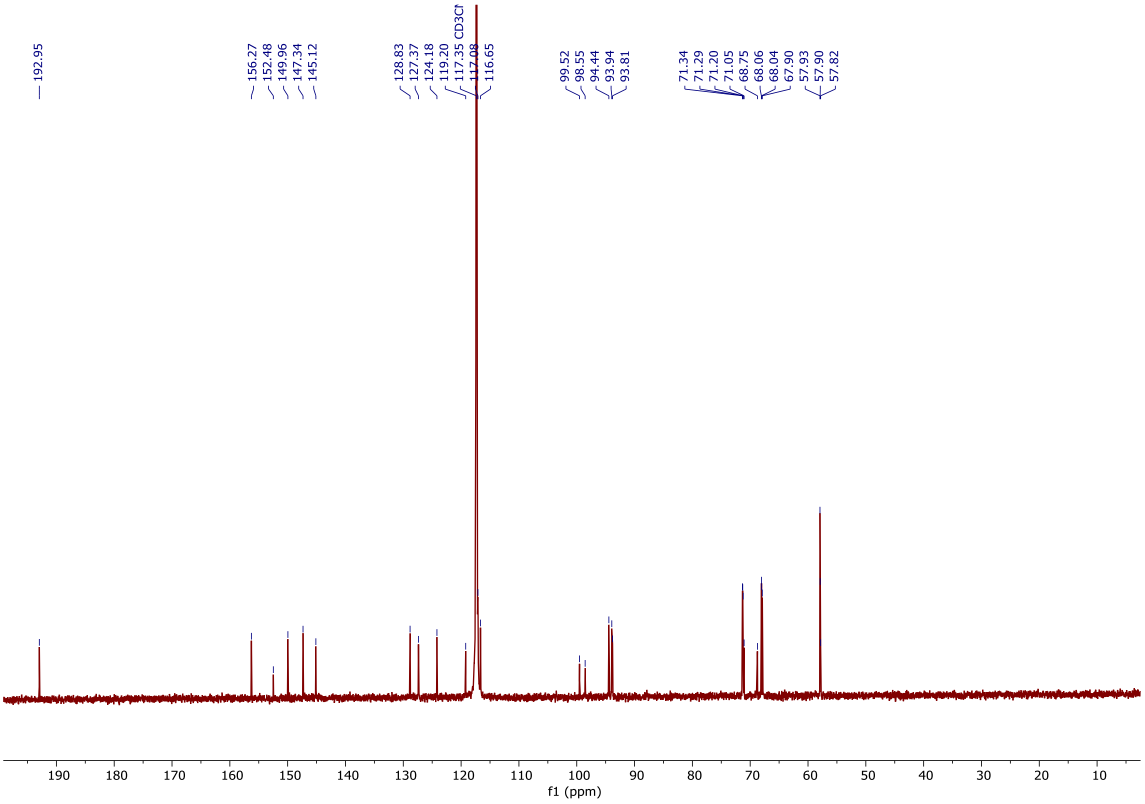

Figure S32: ^{13}C NMR spectrum of compound **24** (75 MHz, CD_3CN).

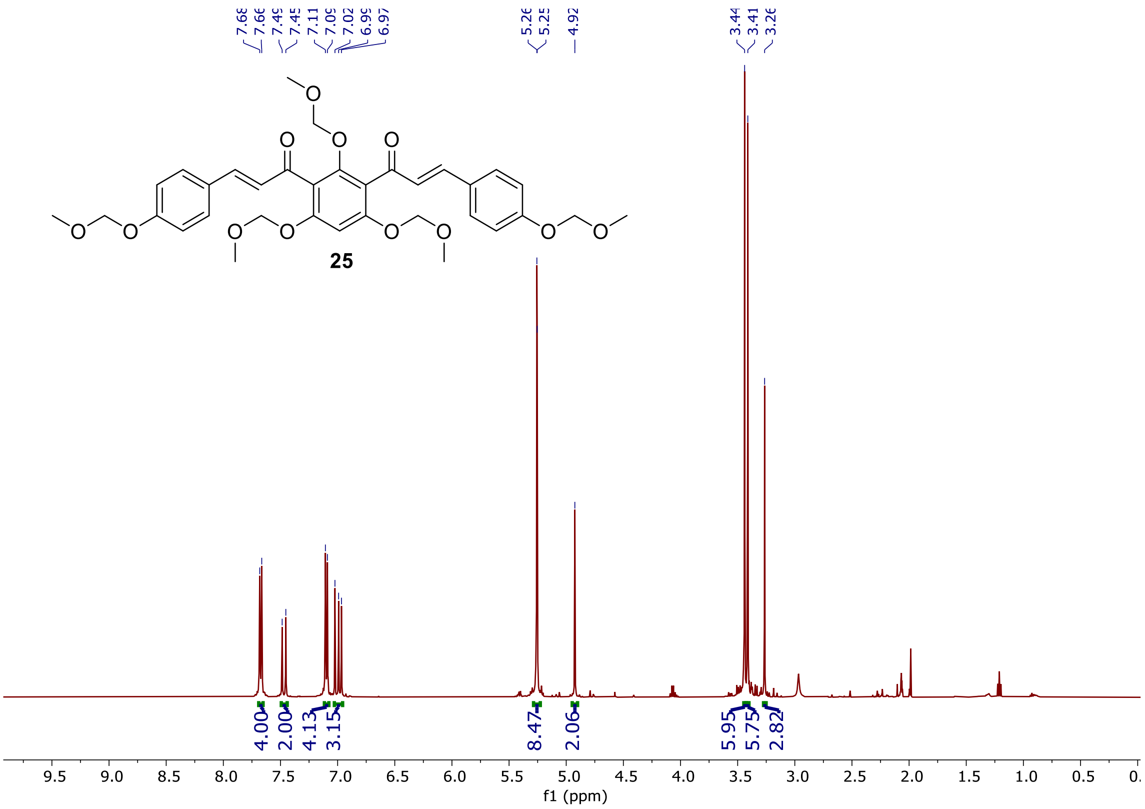

Figure S33: ^{1}H NMR spectrum of compound **25** (300 MHz, Acetone-d_6).

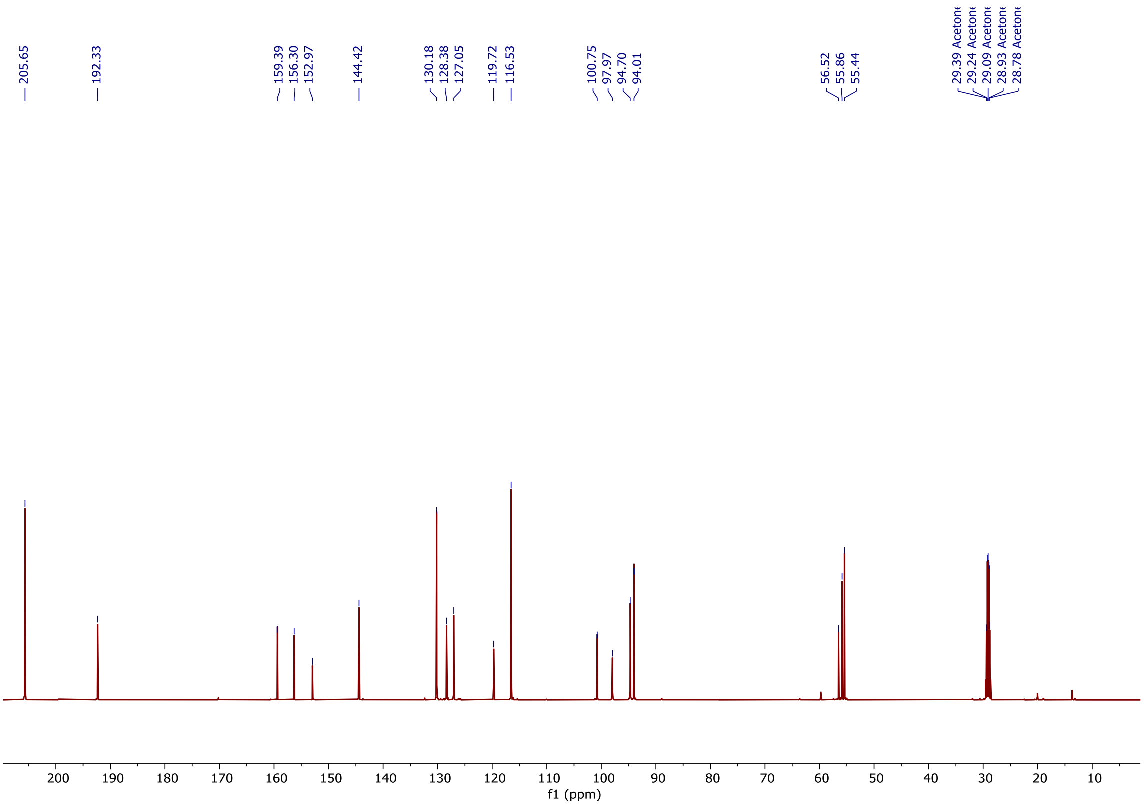

Figure S34: ^{13}C NMR spectrum of compound **25** (75 MHz, Acetone-d_6).

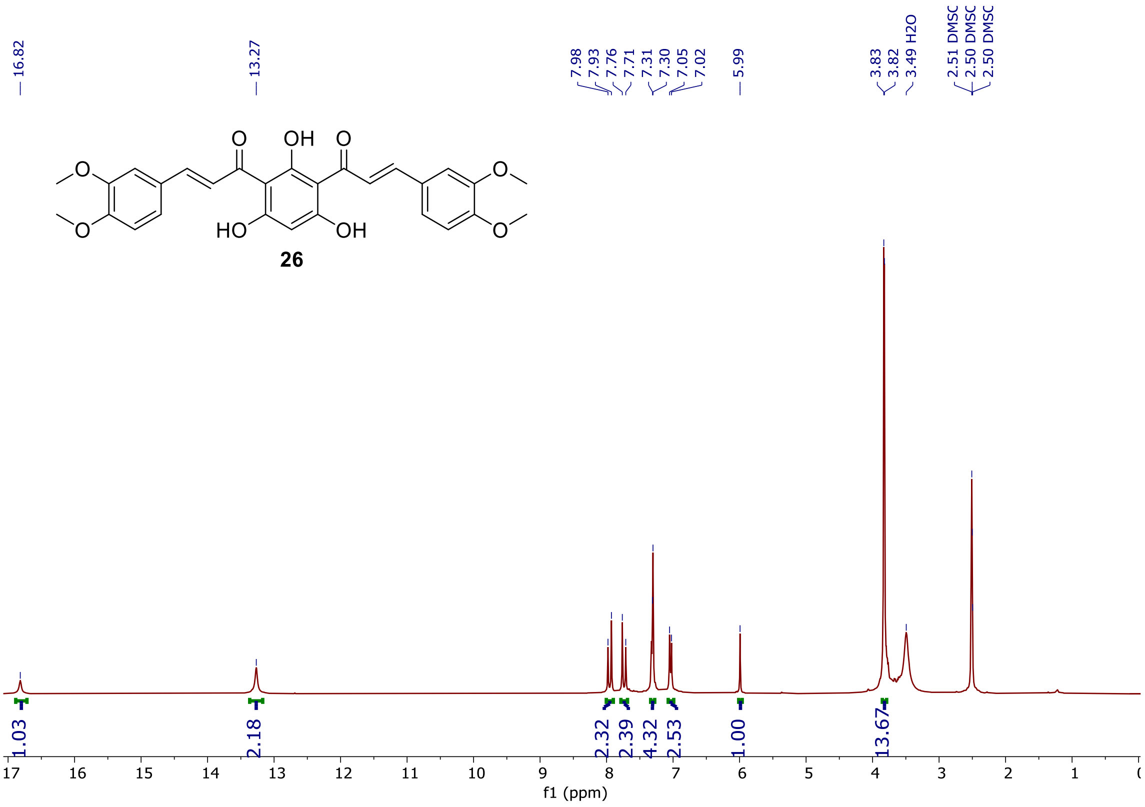

Figure S35: ^{1}H NMR spectrum of compound **26** (300 MHz, DMSO-d_6),

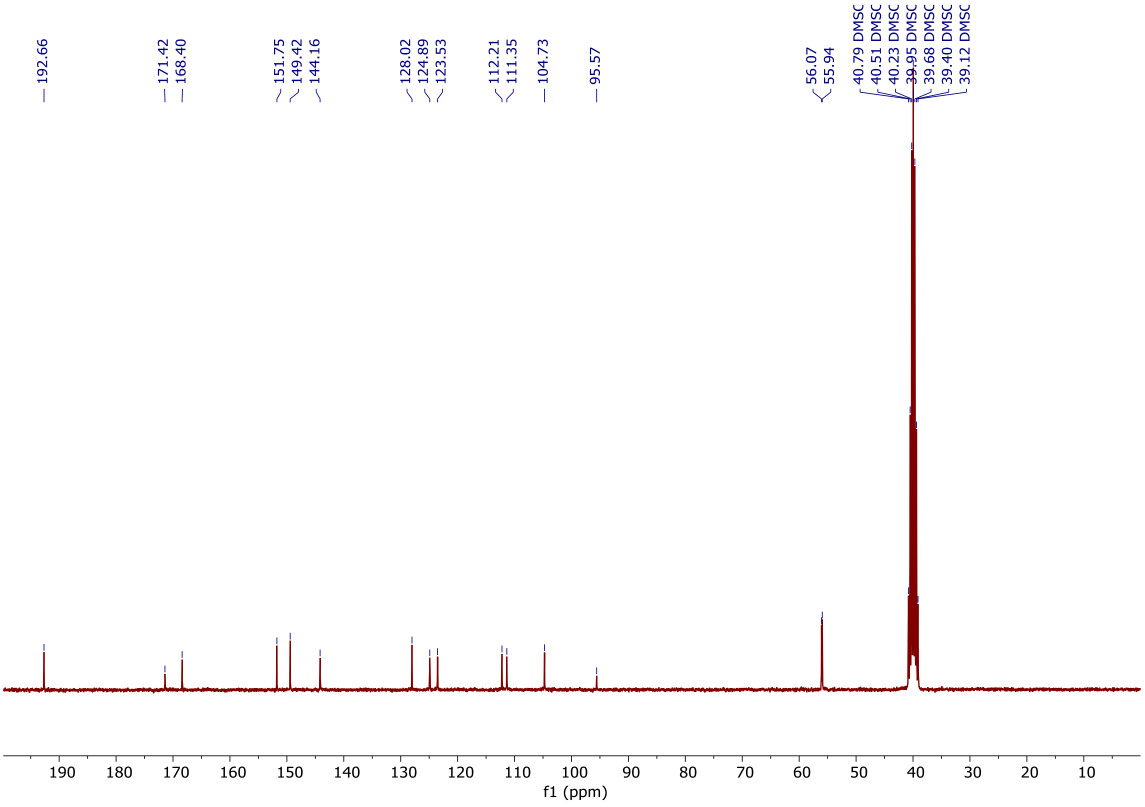

Figure S36: ^{13}C NMR spectrum of compound **26** (75 MHz, DMSO-d_6).

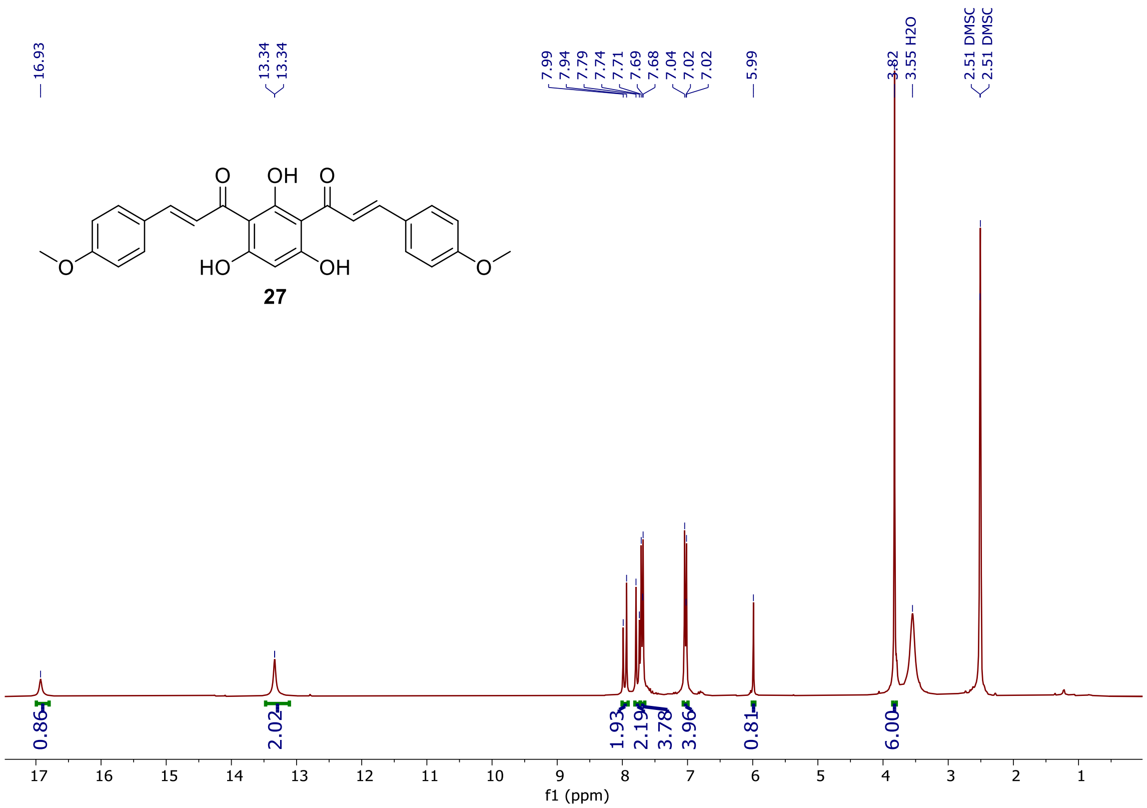

Figure S37: ^{1}H NMR spectrum of compound **27** (300 MHz, DMSO-*d*6),

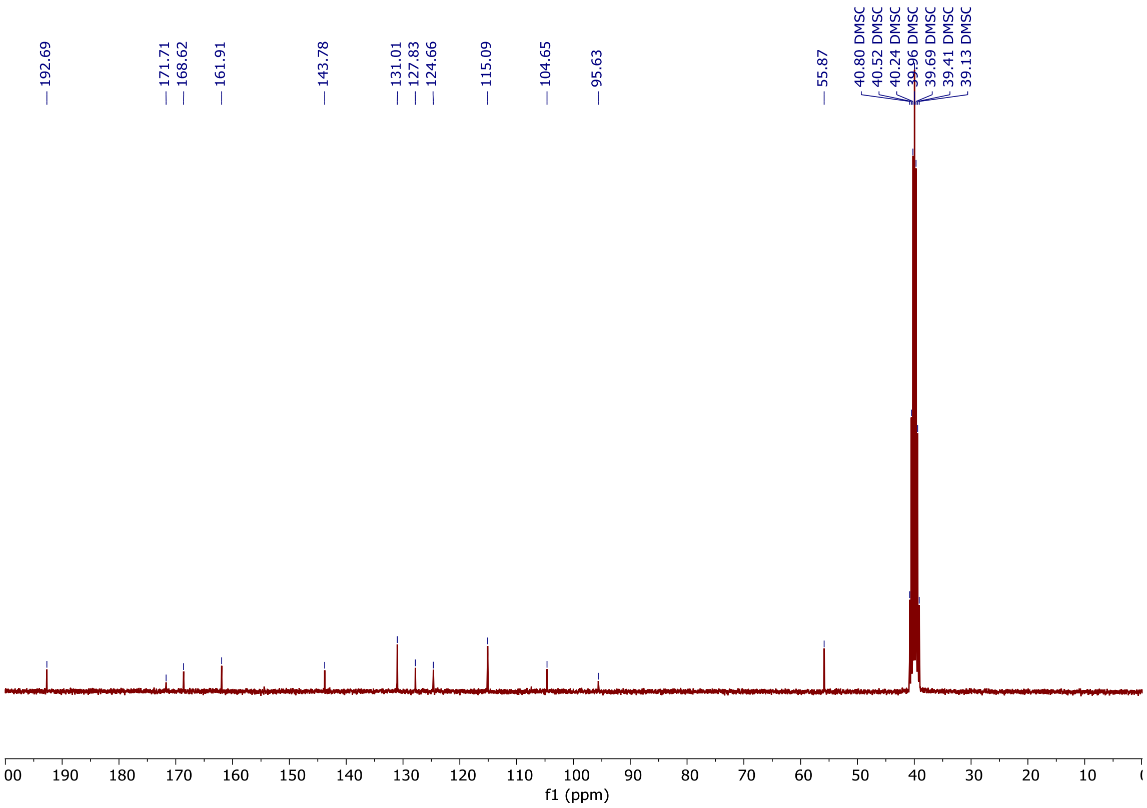

Figure S38: ^{13}C NMR spectrum of compound **27** (75 MHz, DMSO-*d*6).

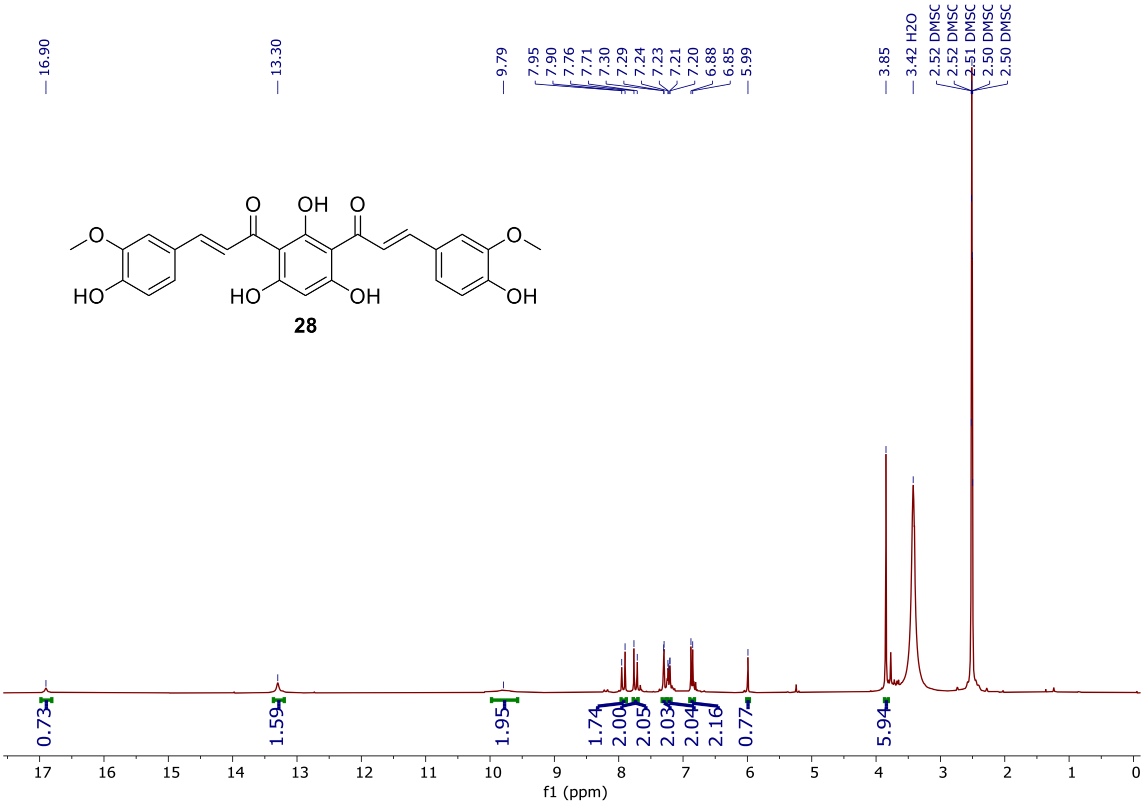

Figure S39: ^{1}H NMR spectrum of compound **28** (300 MHz, DMSO-d_6),

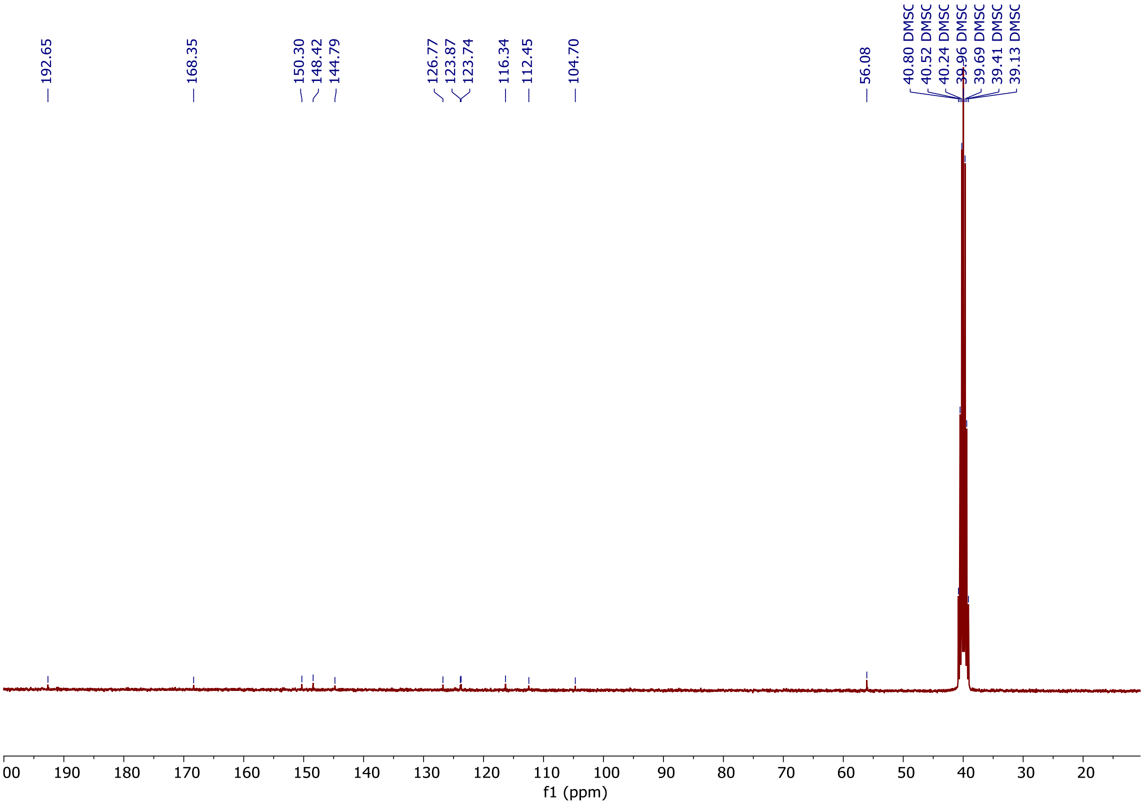

Figure S40: ^{13}C NMR spectrum of compound **28** (75 MHz, DMSO-d_6).

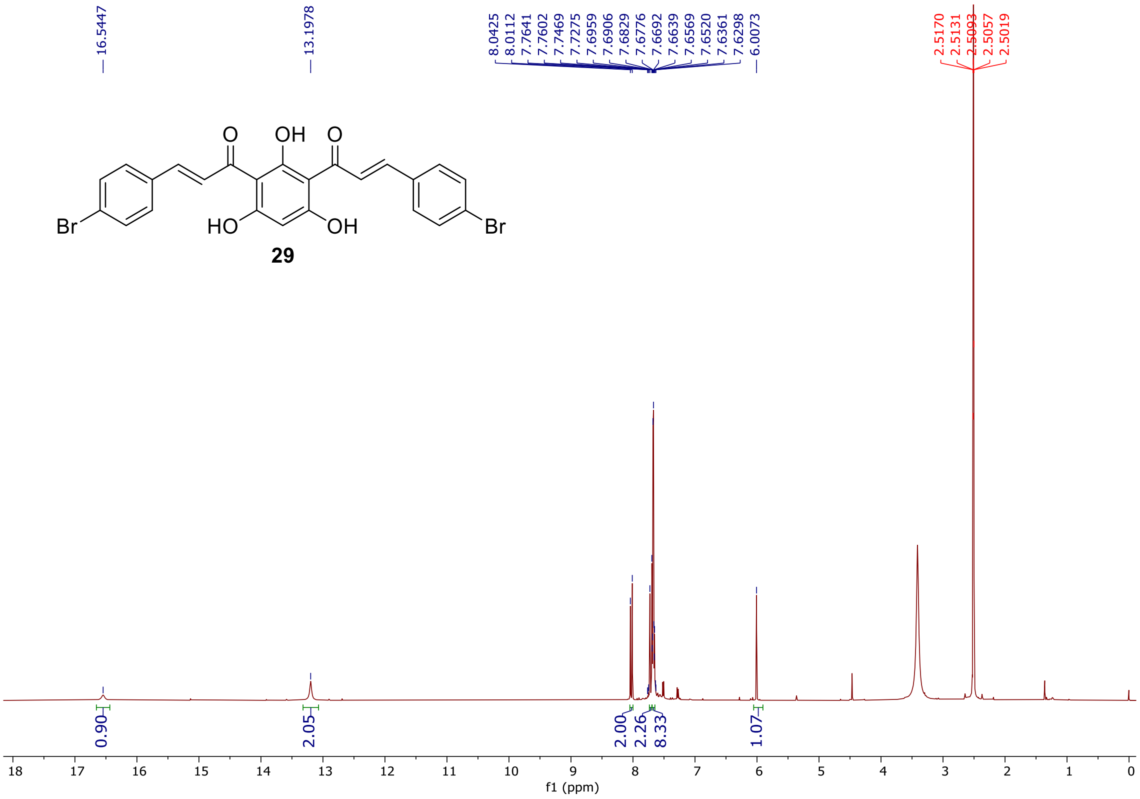

Figure S41: ^{1}H NMR spectrum of compound **29** (300 MHz, DMSO-d_6),

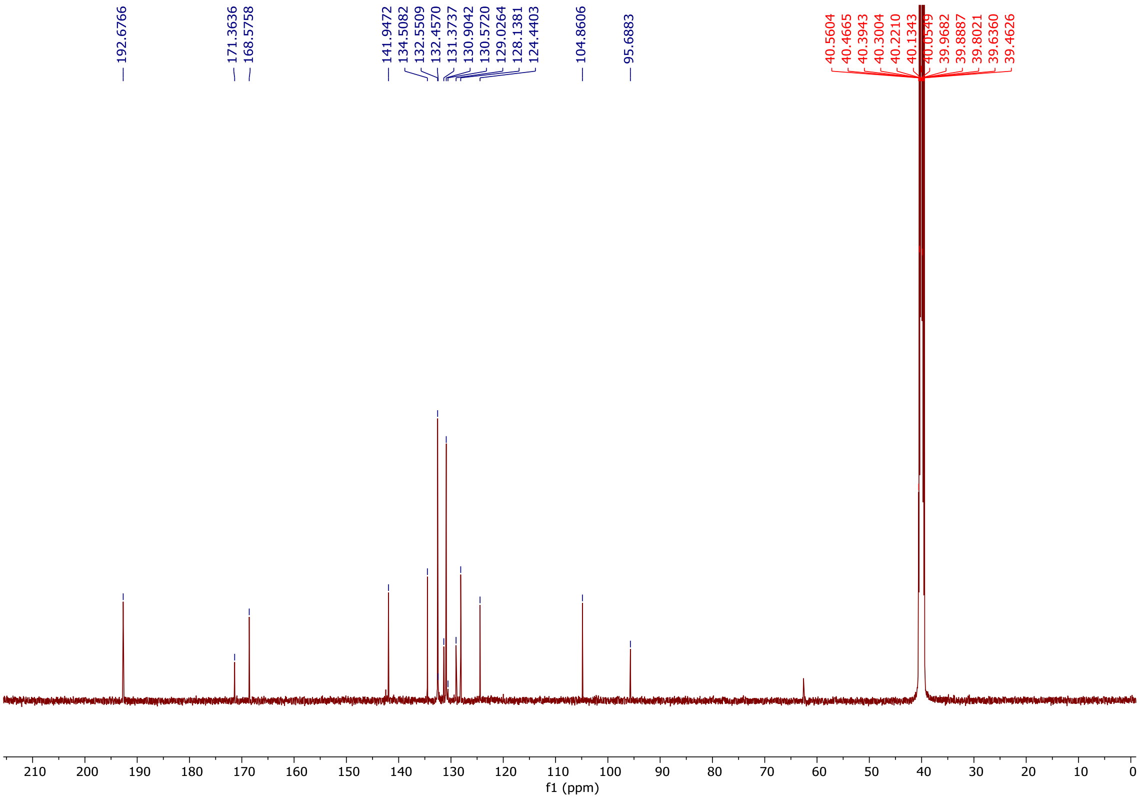

Figure S42: ^{13}C NMR spectrum of compound **29** (75 MHz, DMSO-d_6).

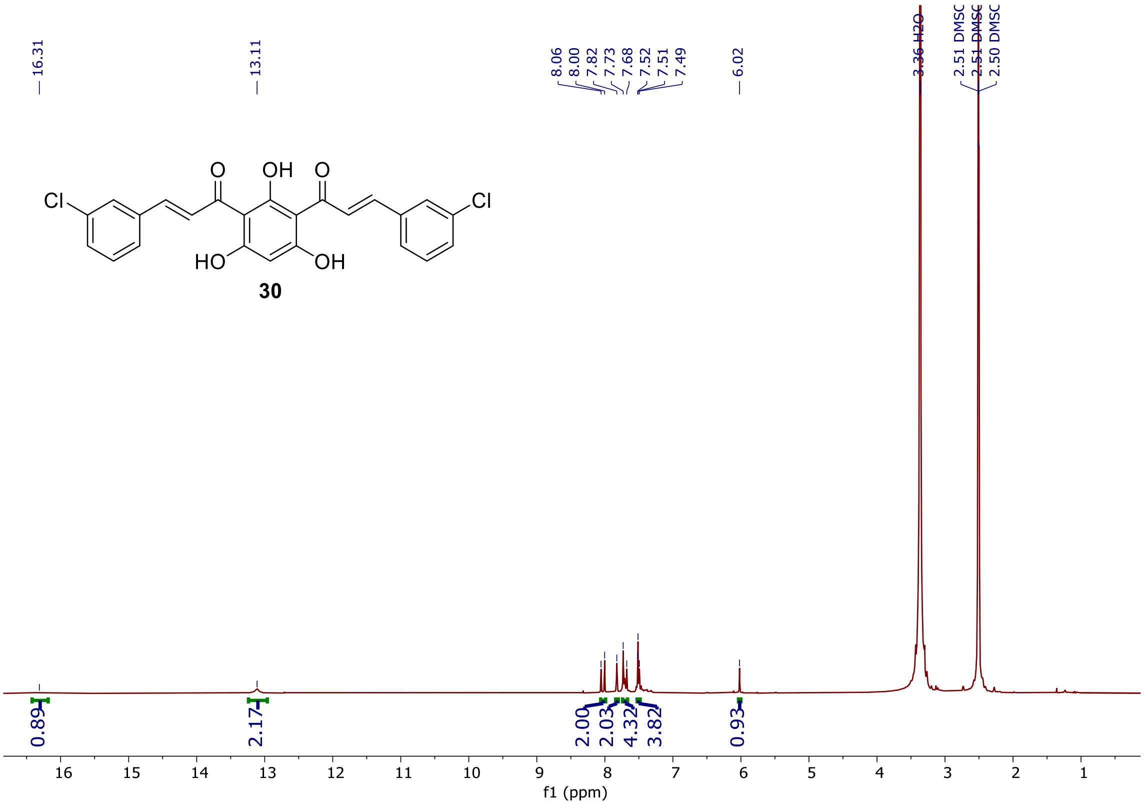

Figure S43: ^{1}H NMR spectrum of compound **30** (300 MHz, DMSO-d_6)

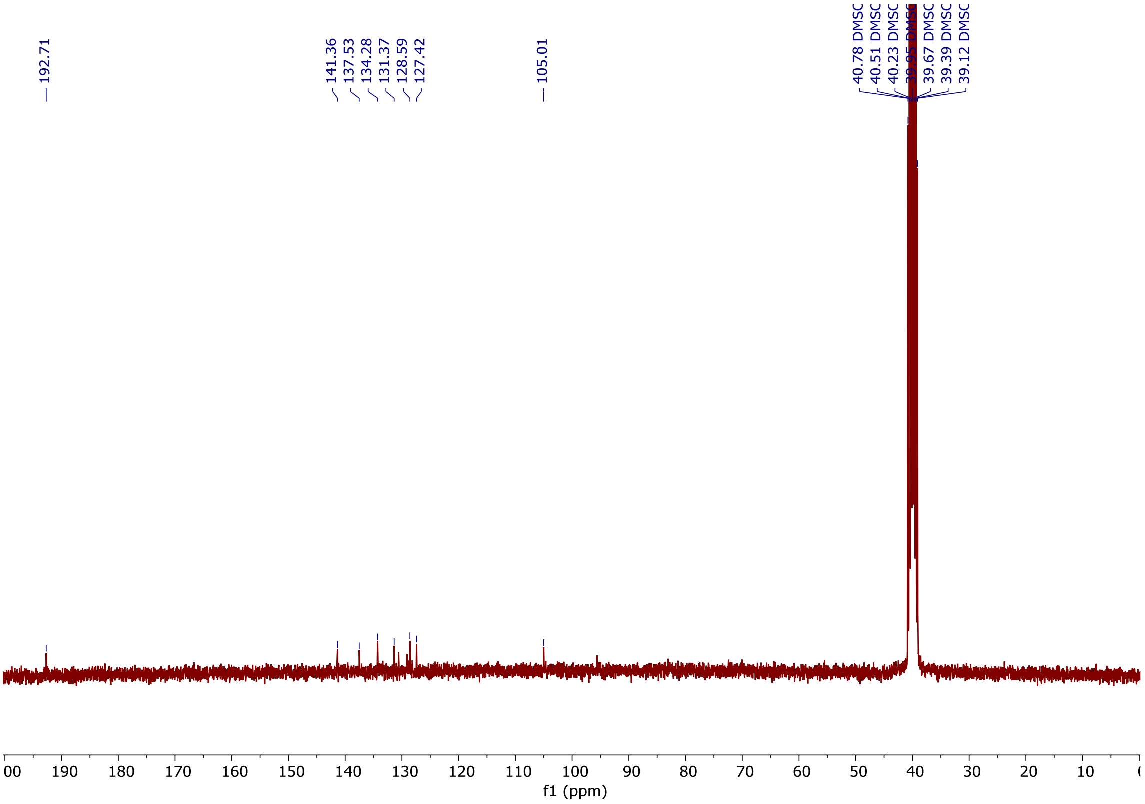

Figure S44: ^{13}C NMR spectrum of compound **30** (75 MHz, DMSO-d_6).

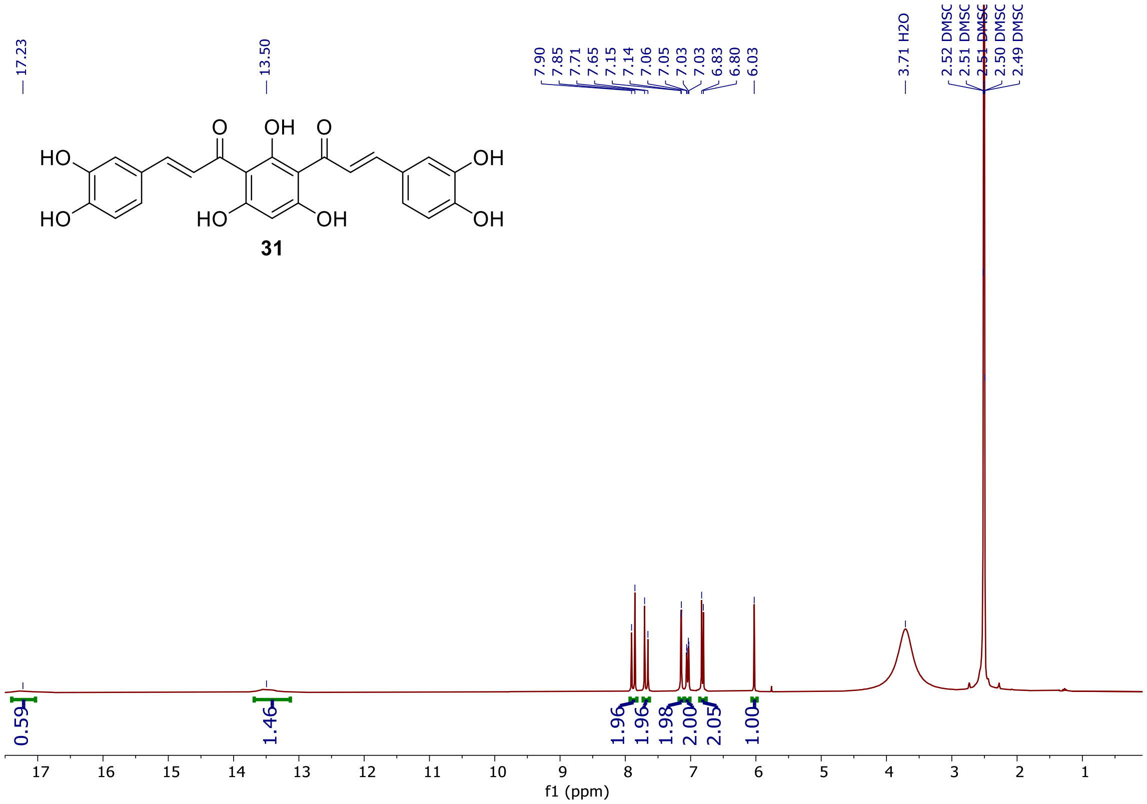

Figure S45: ^{1}H NMR spectrum of compound **31** (300 MHz, DMSO-d_6)

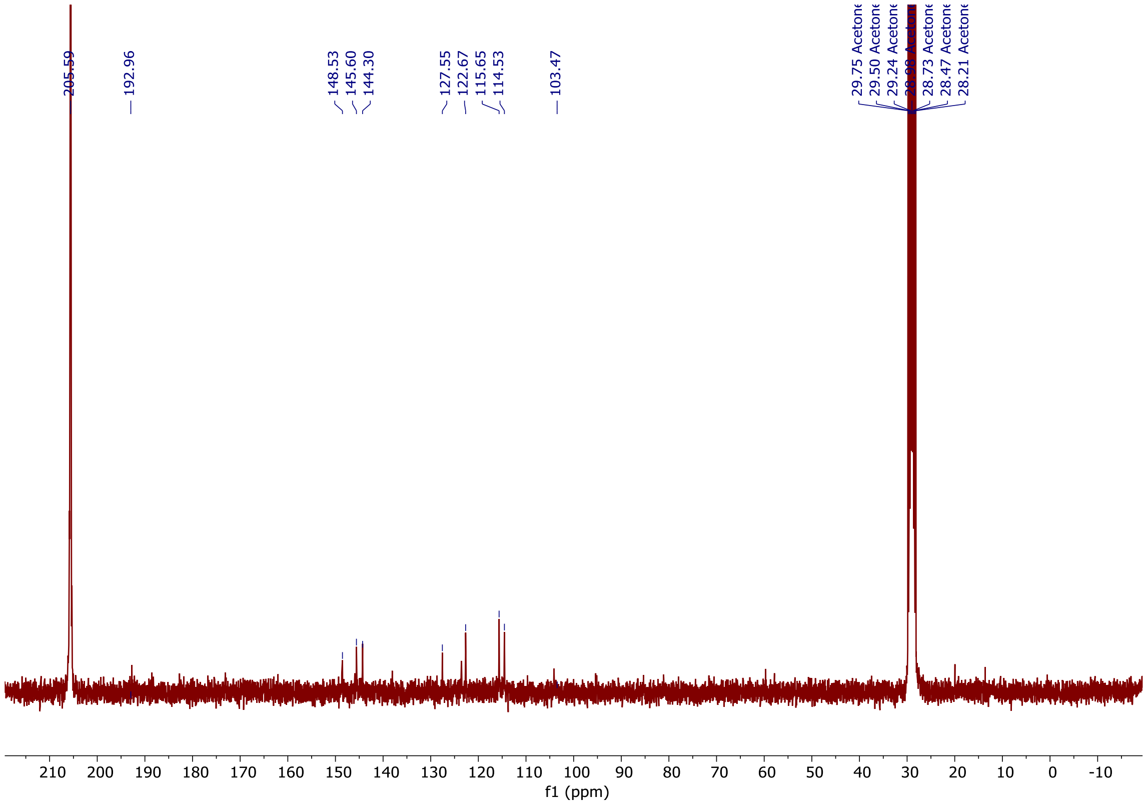

Figure S46: ^{13}C NMR spectrum of compound **31** (75 MHz, DMSO-d_6).

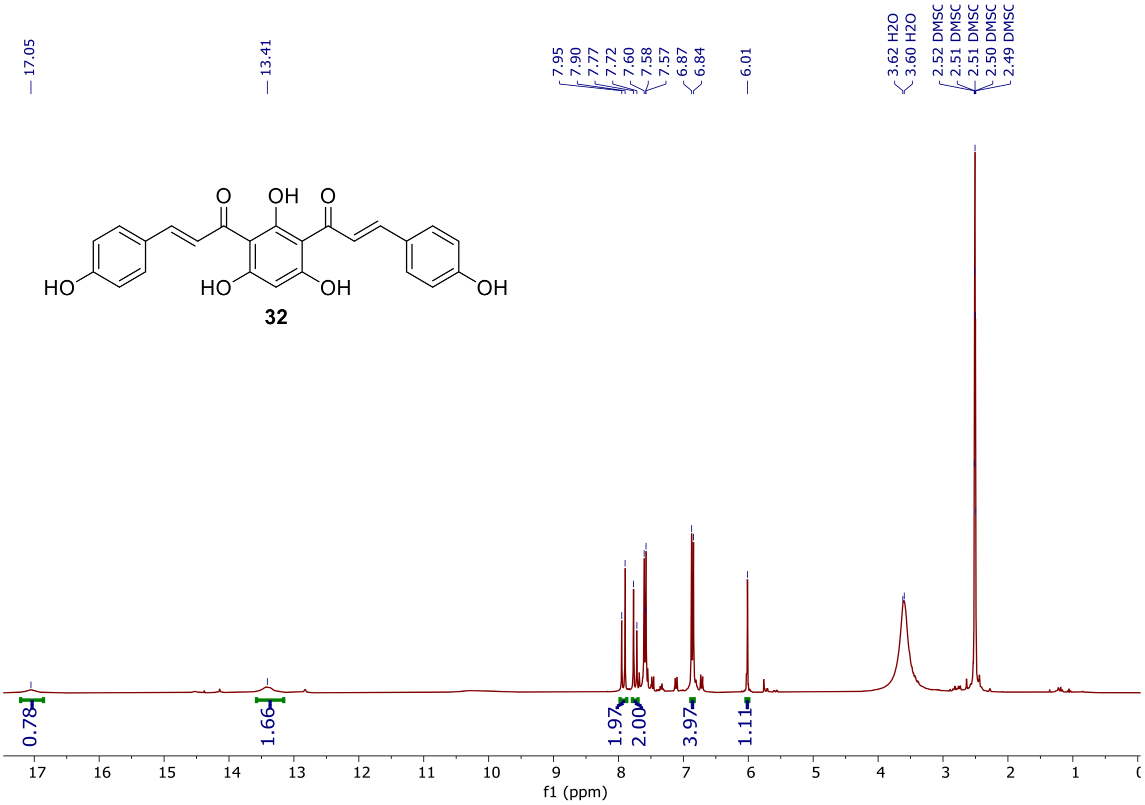

Figure S47: ^{1}H NMR spectrum of compound **32** (300 MHz, DMSO-*d*$_6$)

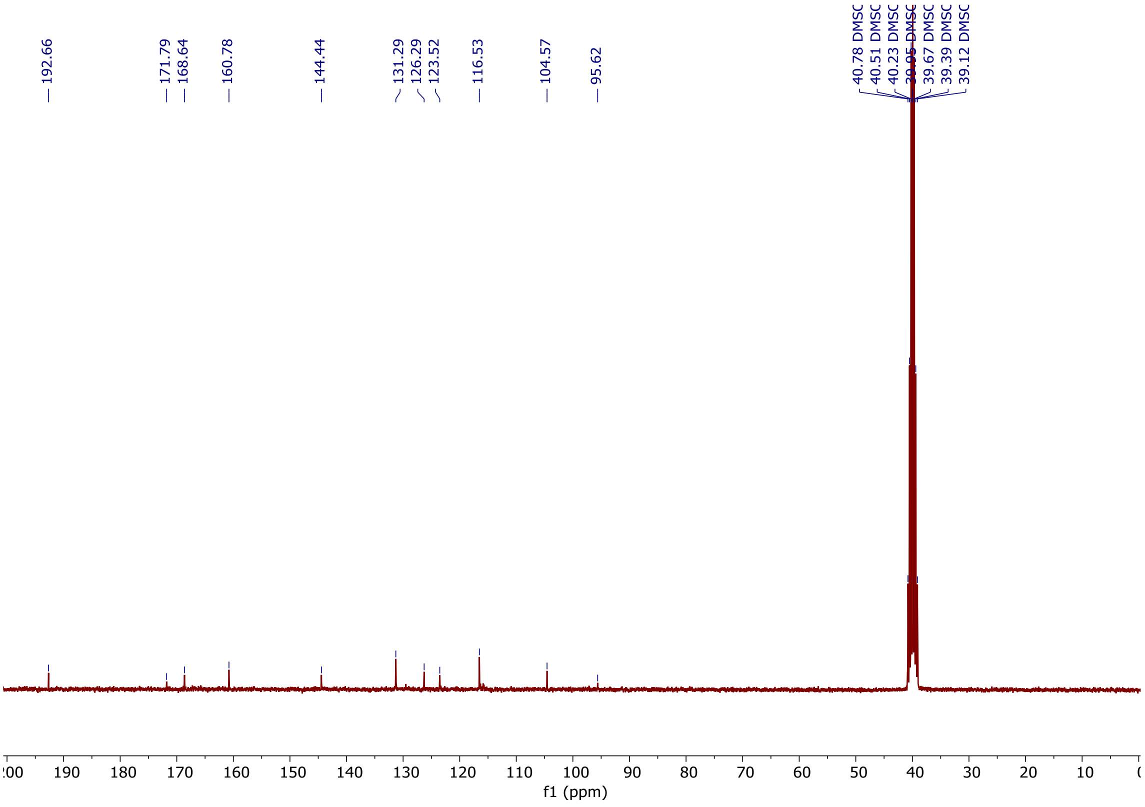

Figure S48: ^{13}C NMR spectrum of compound **32** (75 MHz, DMSO-*d*$_6$).

Mass spectra

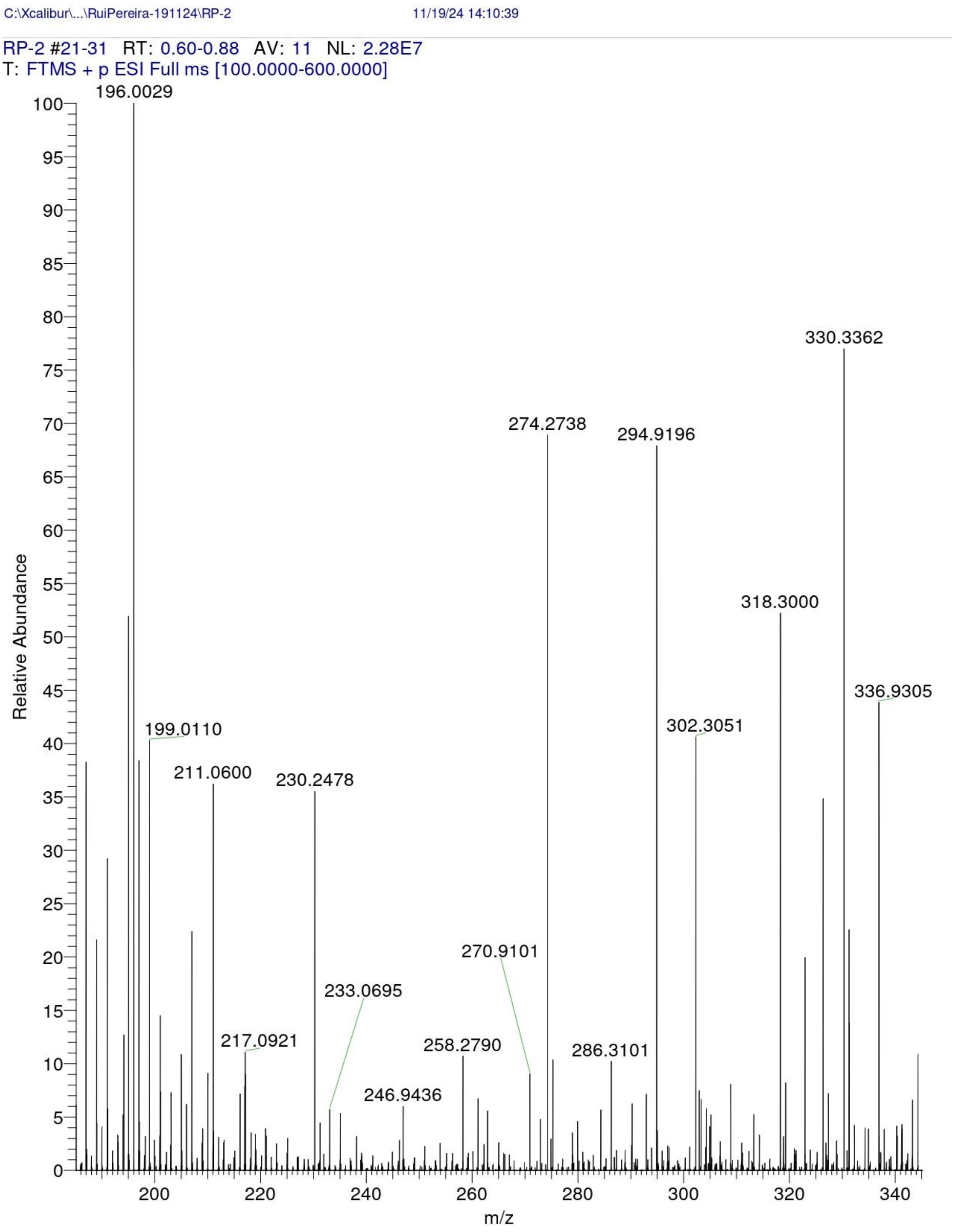

Figure S49: Mass spectrum of compound **2.**

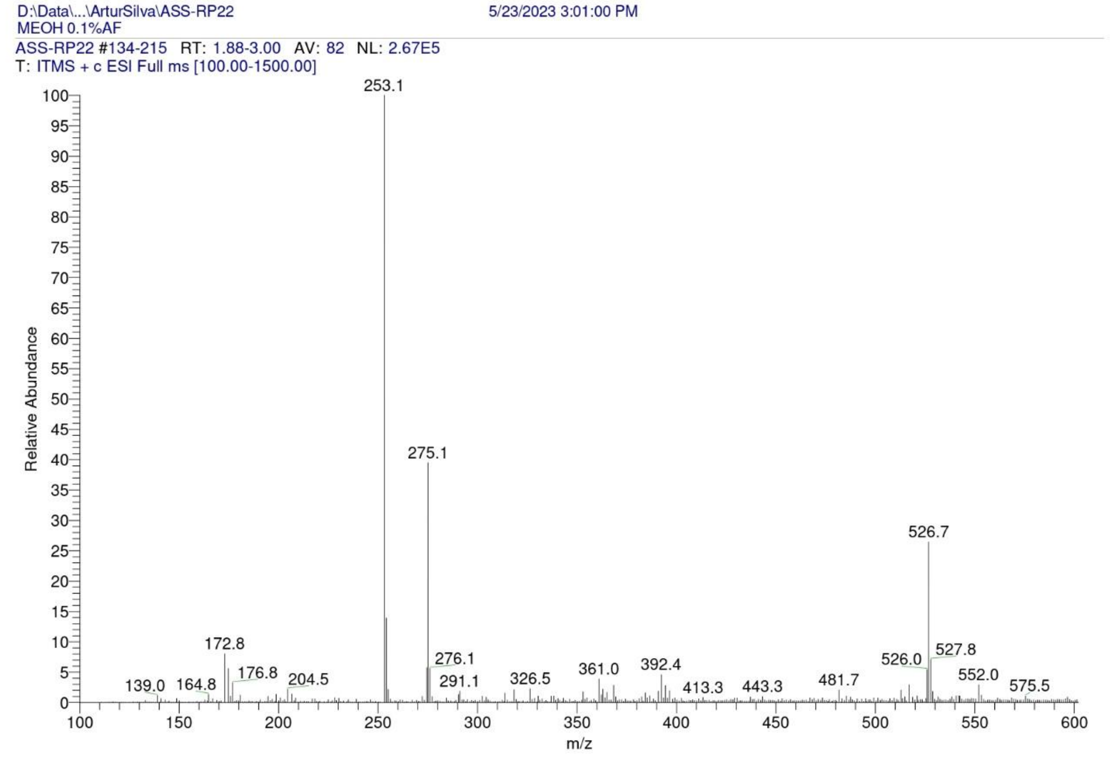

Figure S50: Mass spectrum of compound **3.**

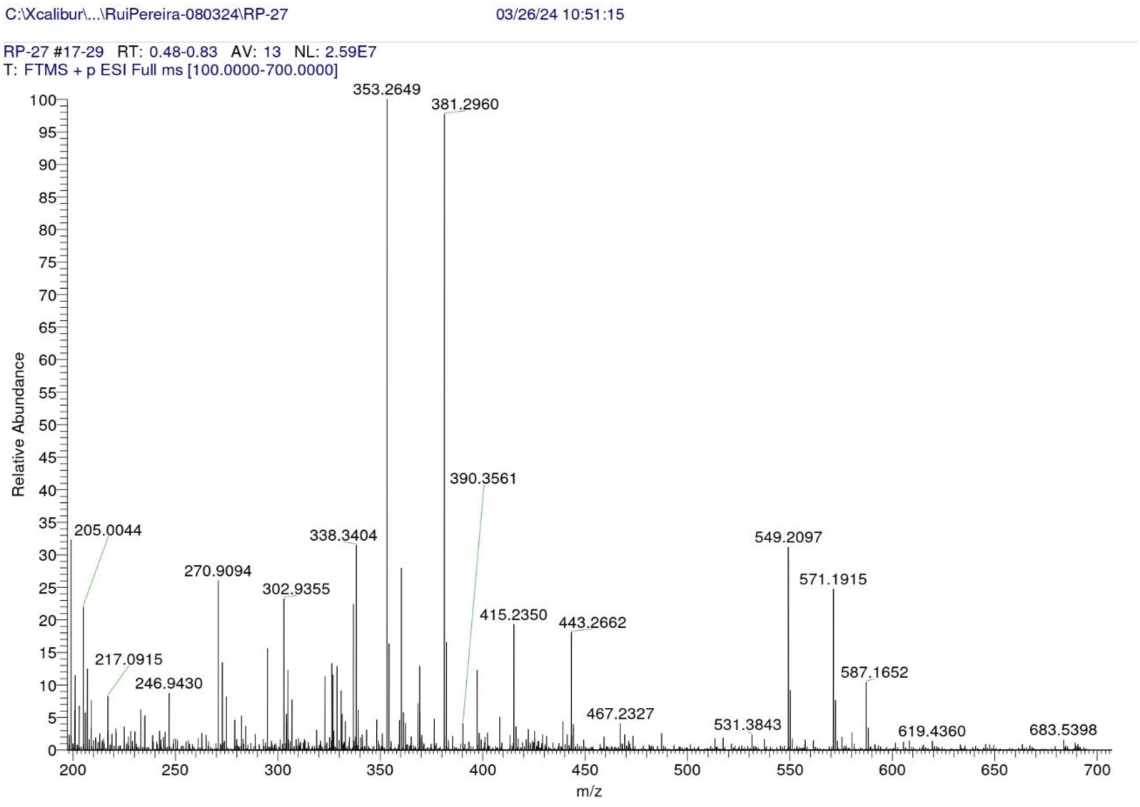

Figure S51: Mass spectrum of compound **10.**

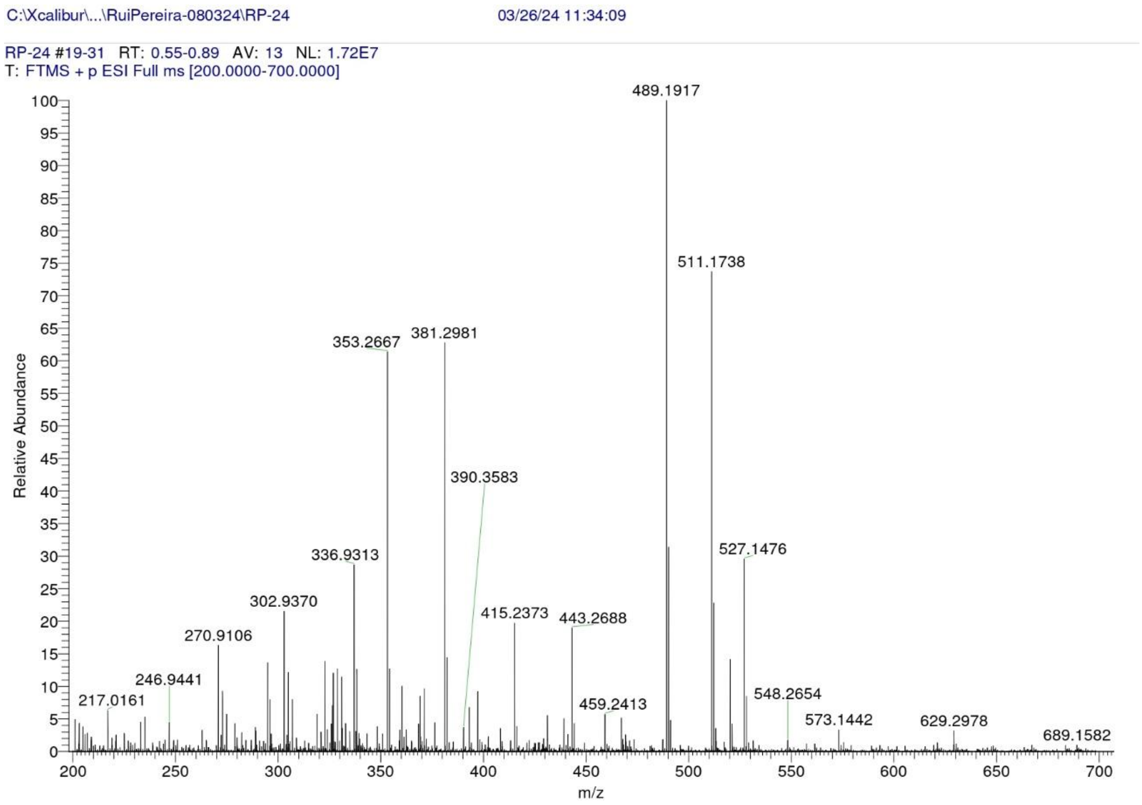

Figure S52: Mass spectrum of compound **11.**

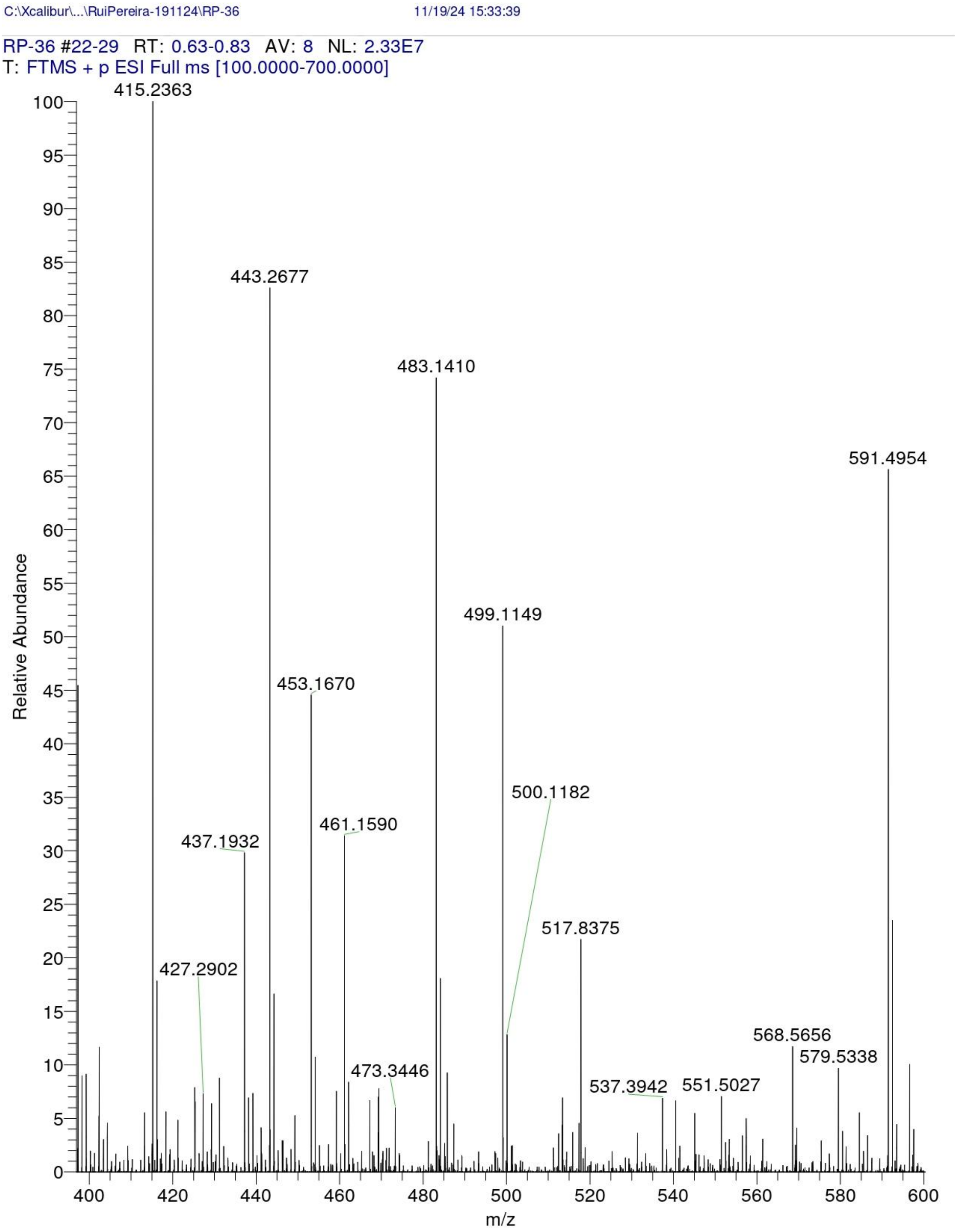

Figure S53: Mass spectrum of compound **12.**

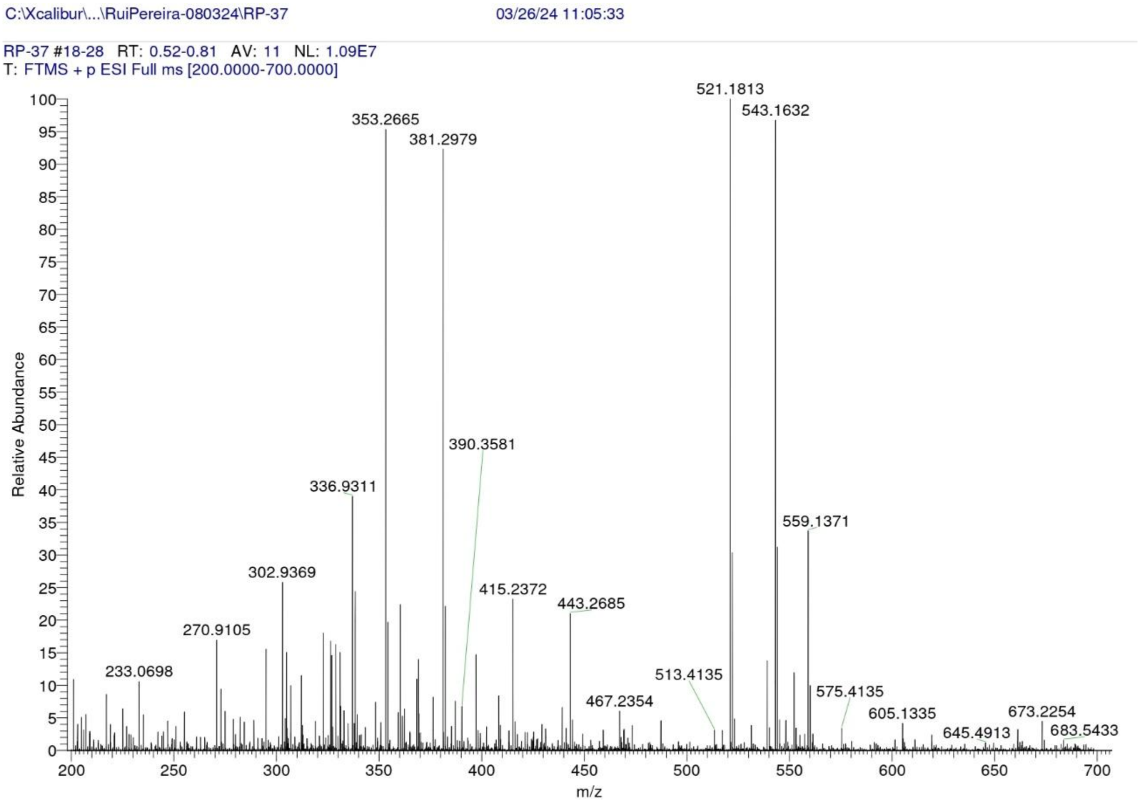

Figure S54: Mass spectrum of compound **13.**

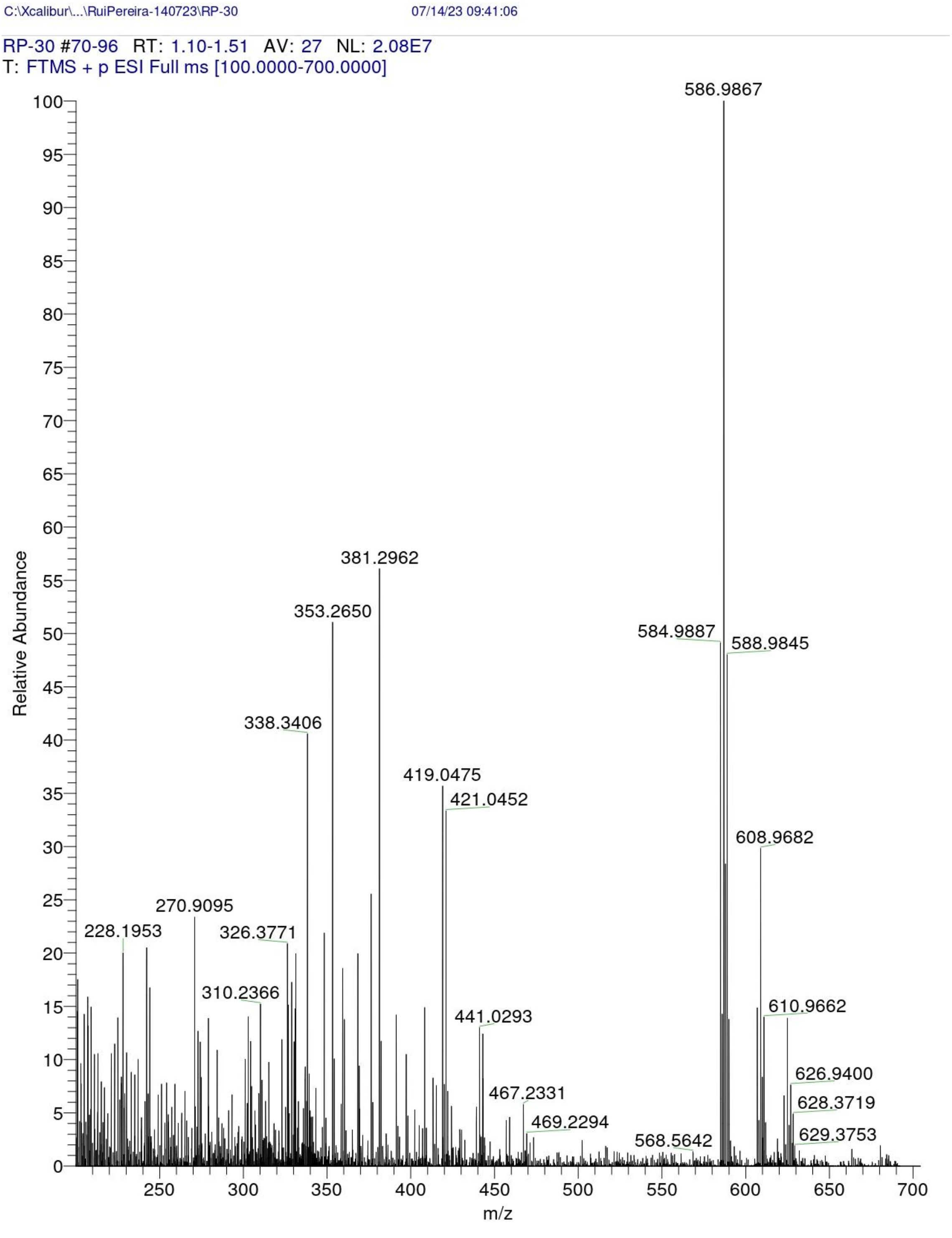

Figure S55: Mass spectrum of compound **14.**

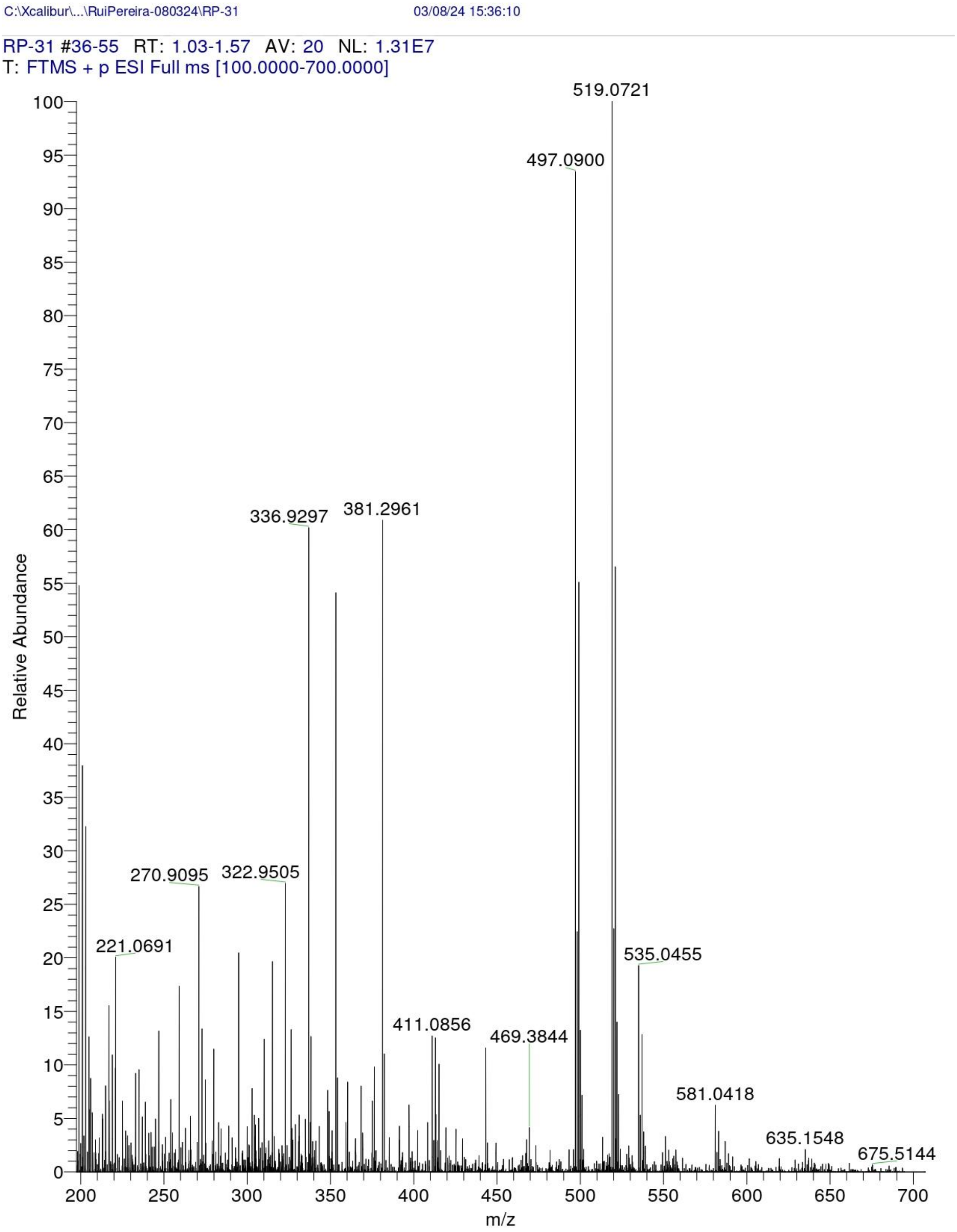

Figure S56: Mass spectrum of compound **15.**

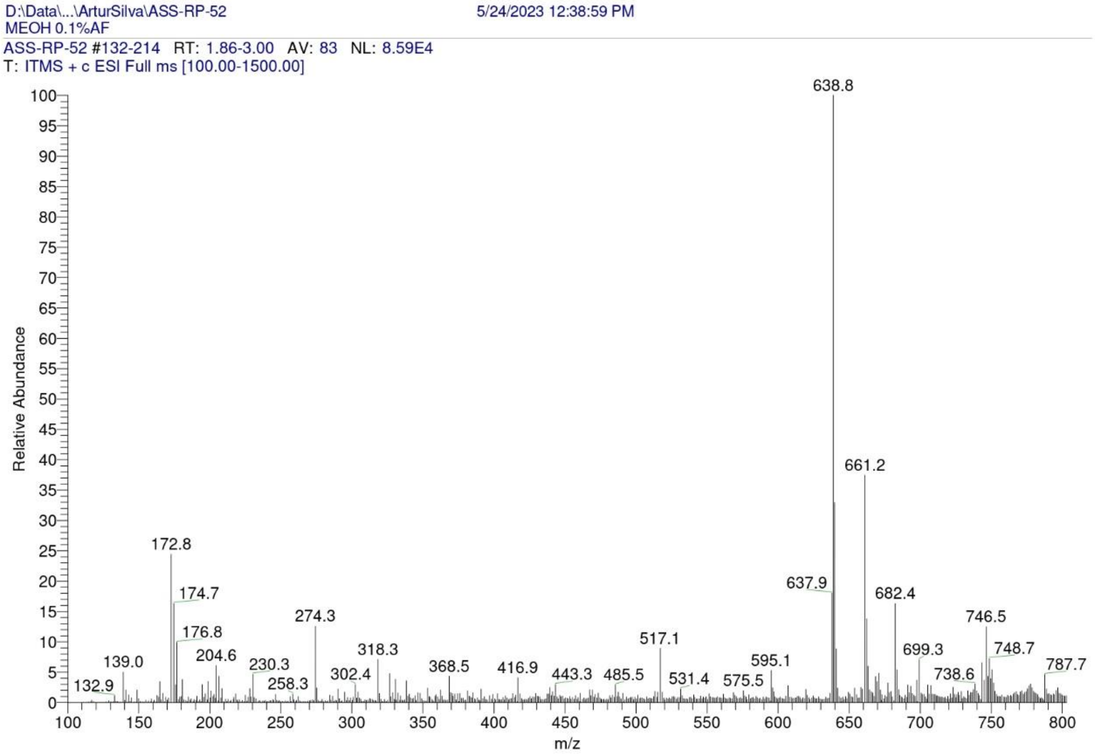

Figure S57: Mass spectrum of compound **19**

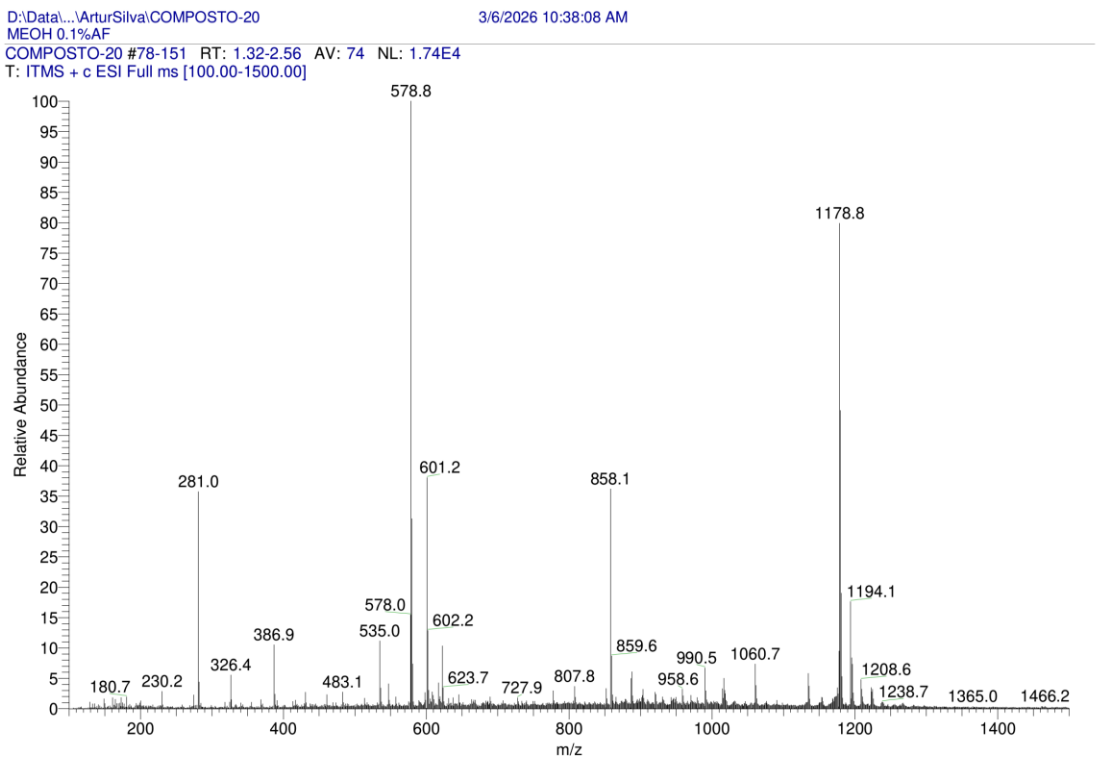

Figure S58: Mass spectrum of compound **20.**

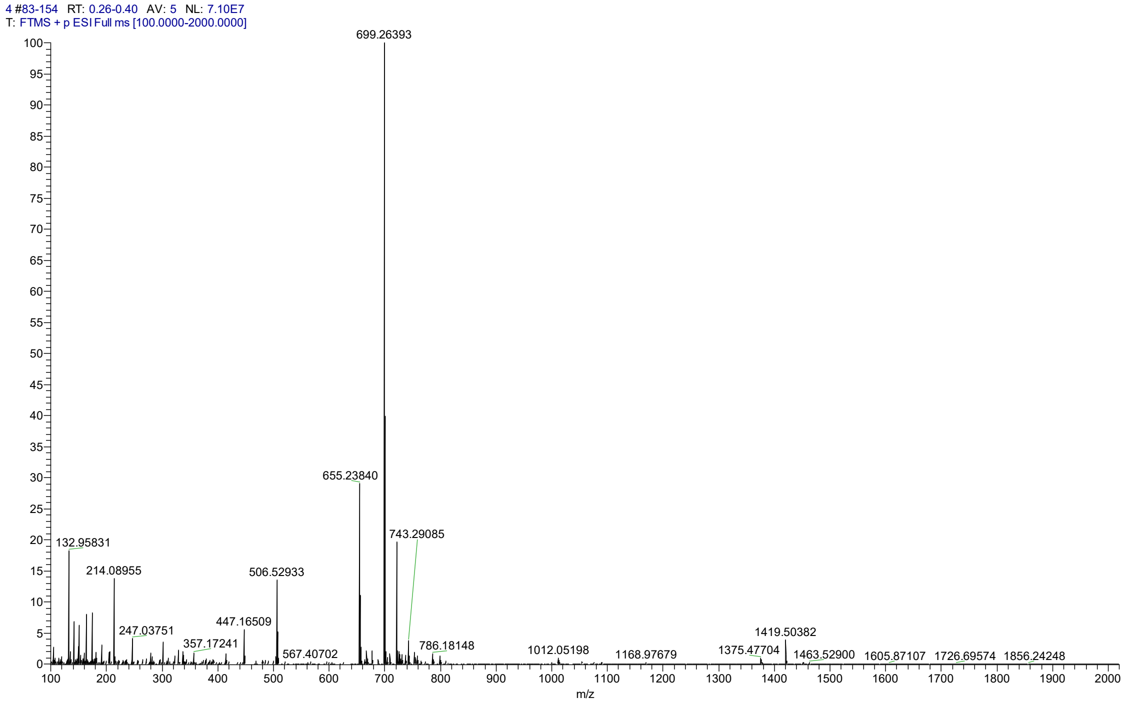

Figure S59: Mass spectrum of compound **21**

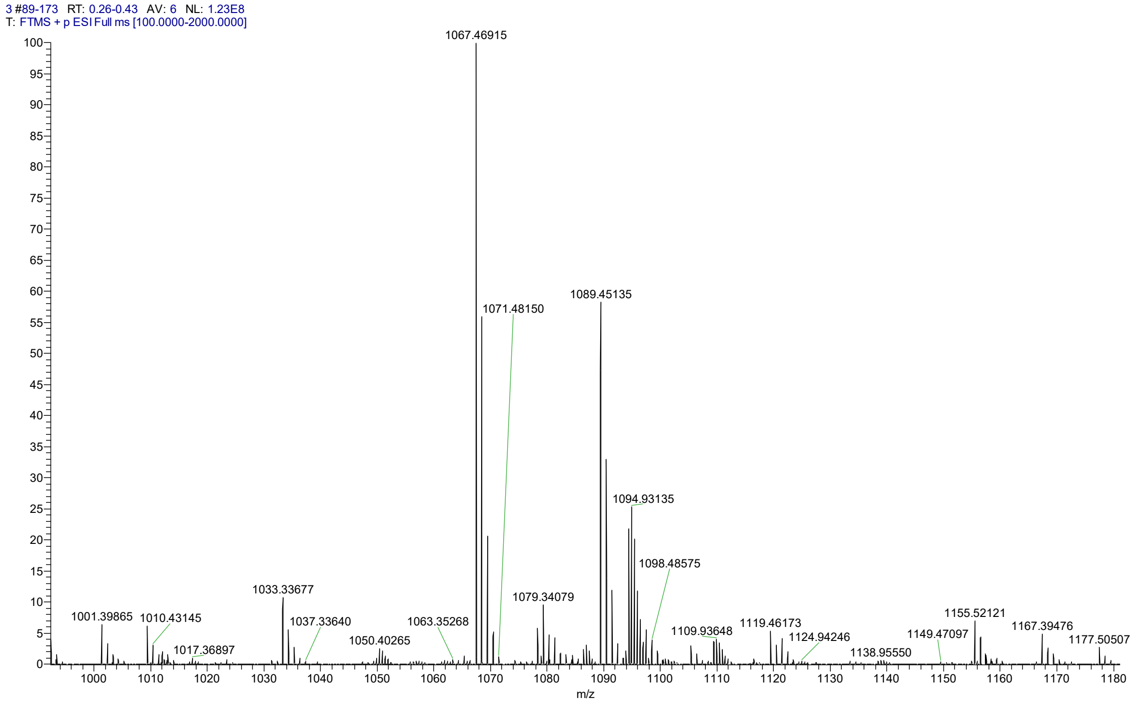

Figure S60: Mass spectrum of compound **24.**

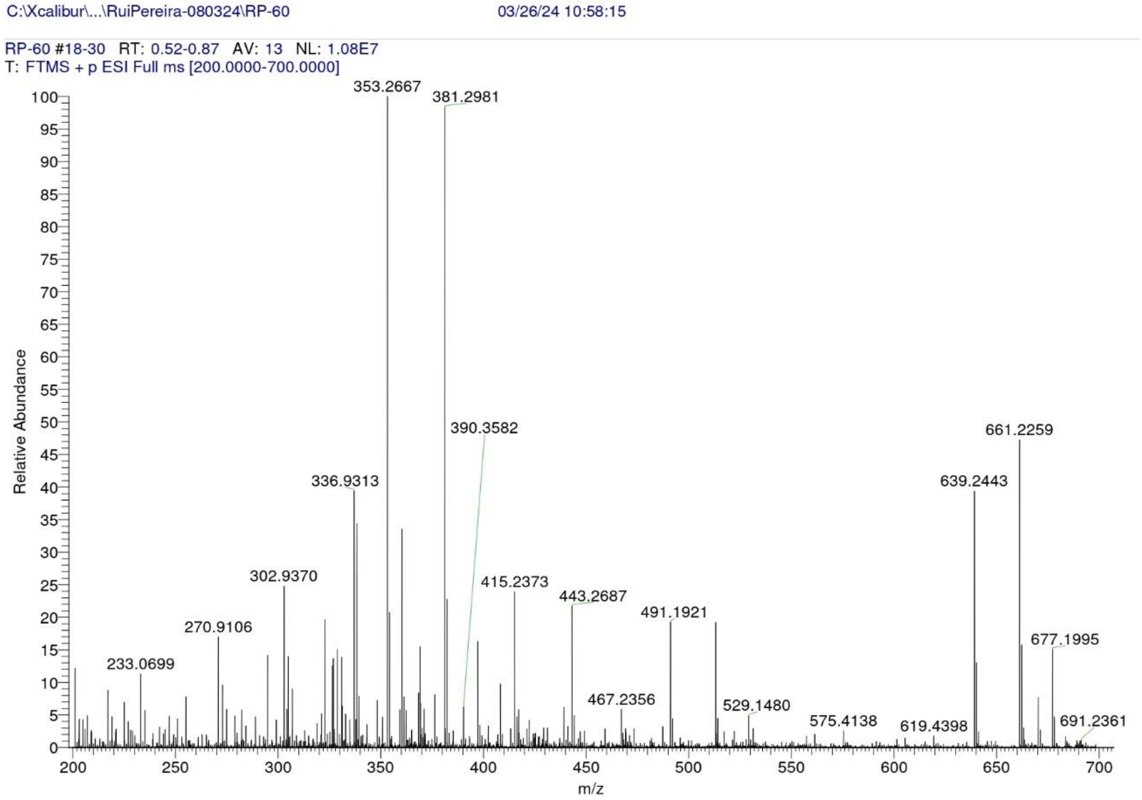

Figure S61: Mass spectrum of compound **25.**

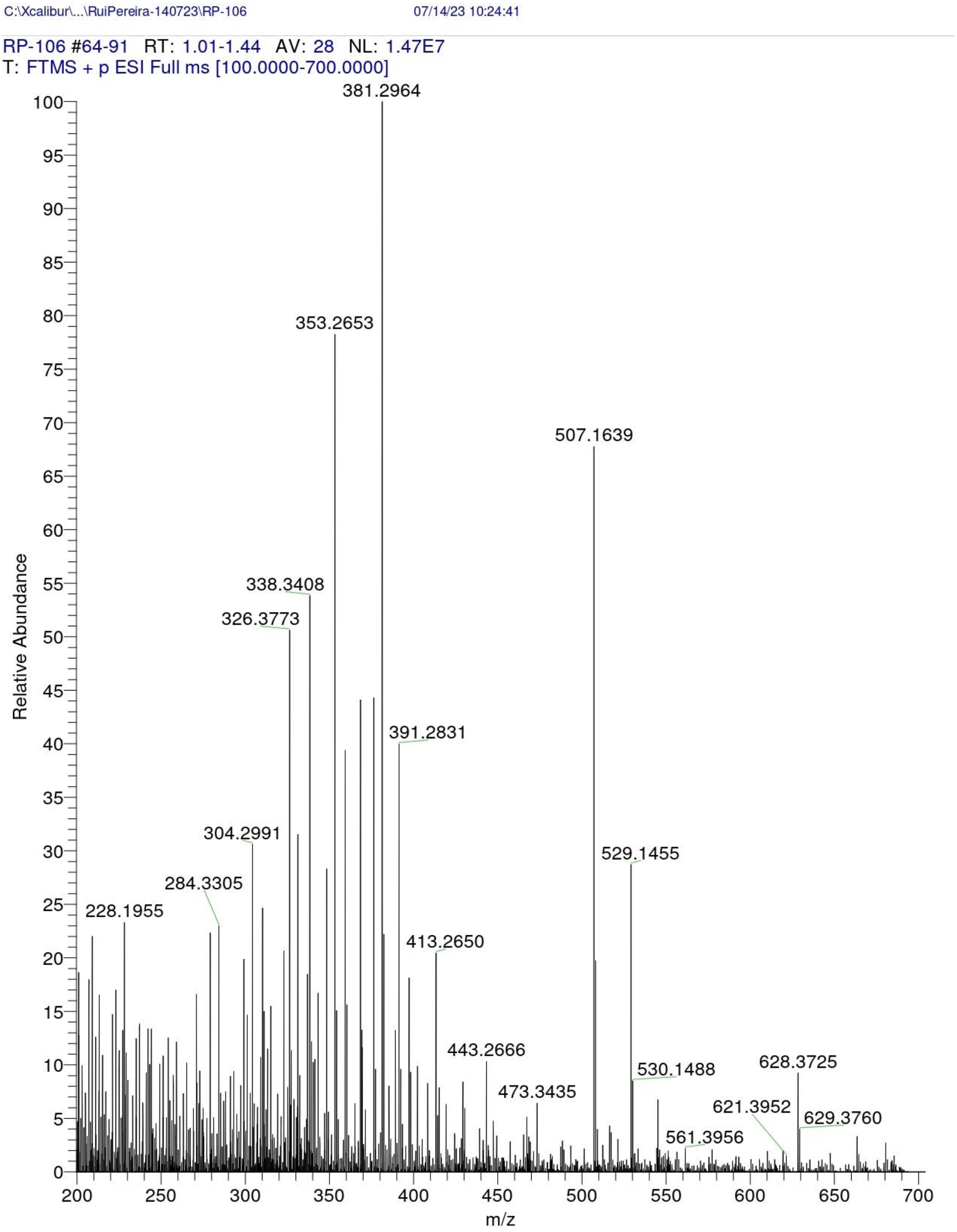

Figure S62: Mass spectrum of compound **26.**

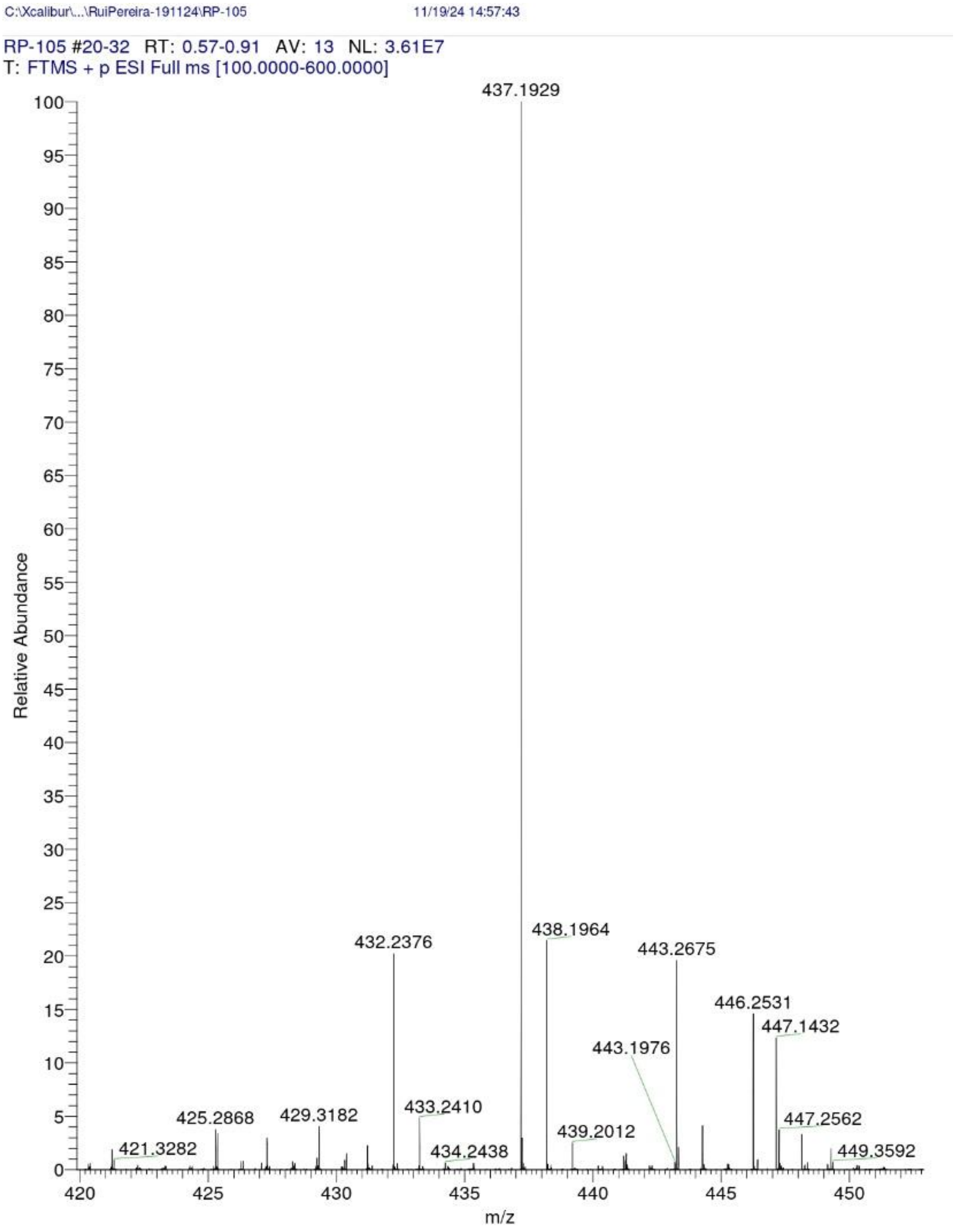

Figure S63: Mass spectrum of compound **27.**

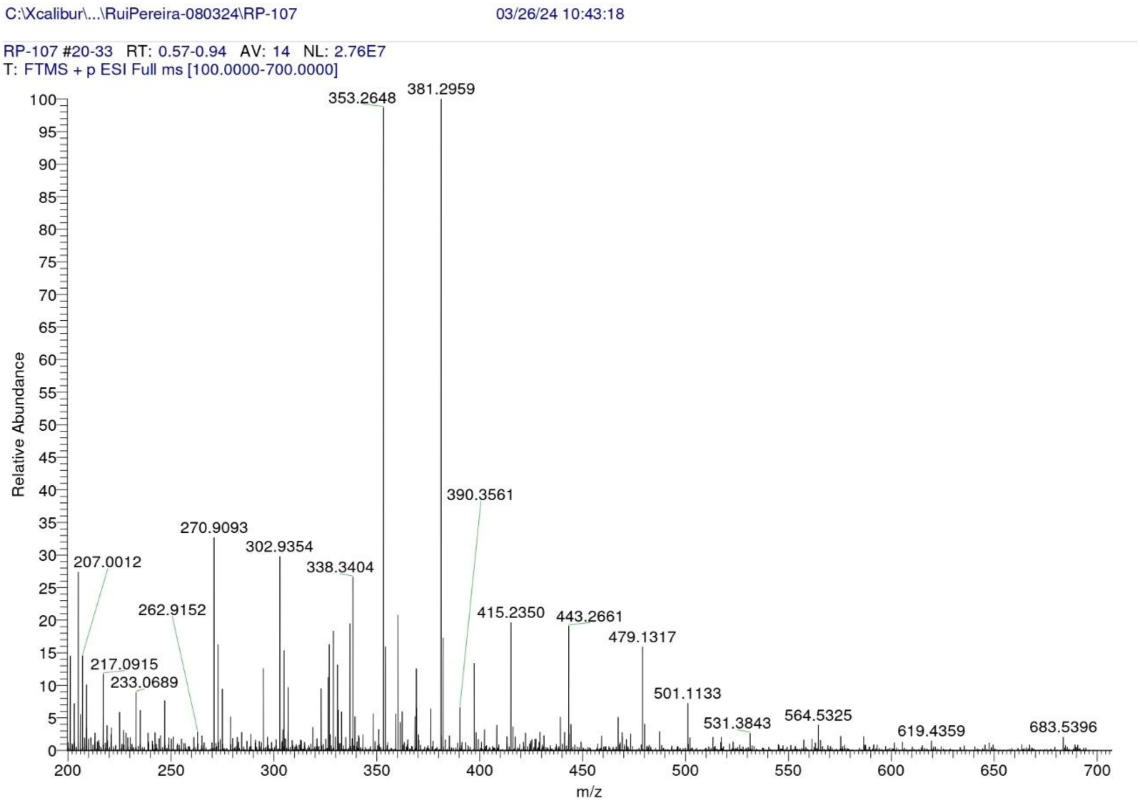

Figure S64: Mass spectrum of compound **28.**

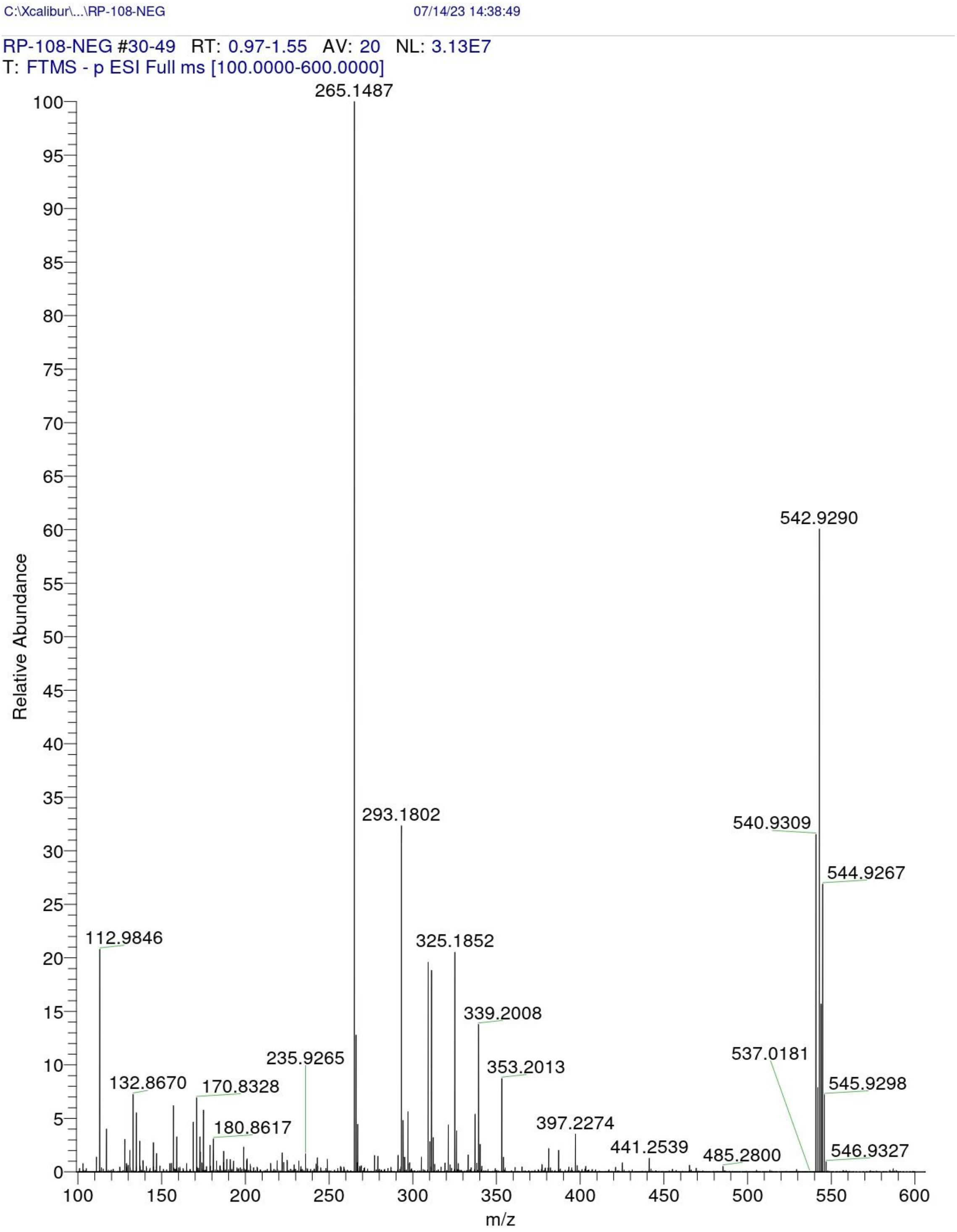

Figure S65: Mass spectrum of compound **29.**

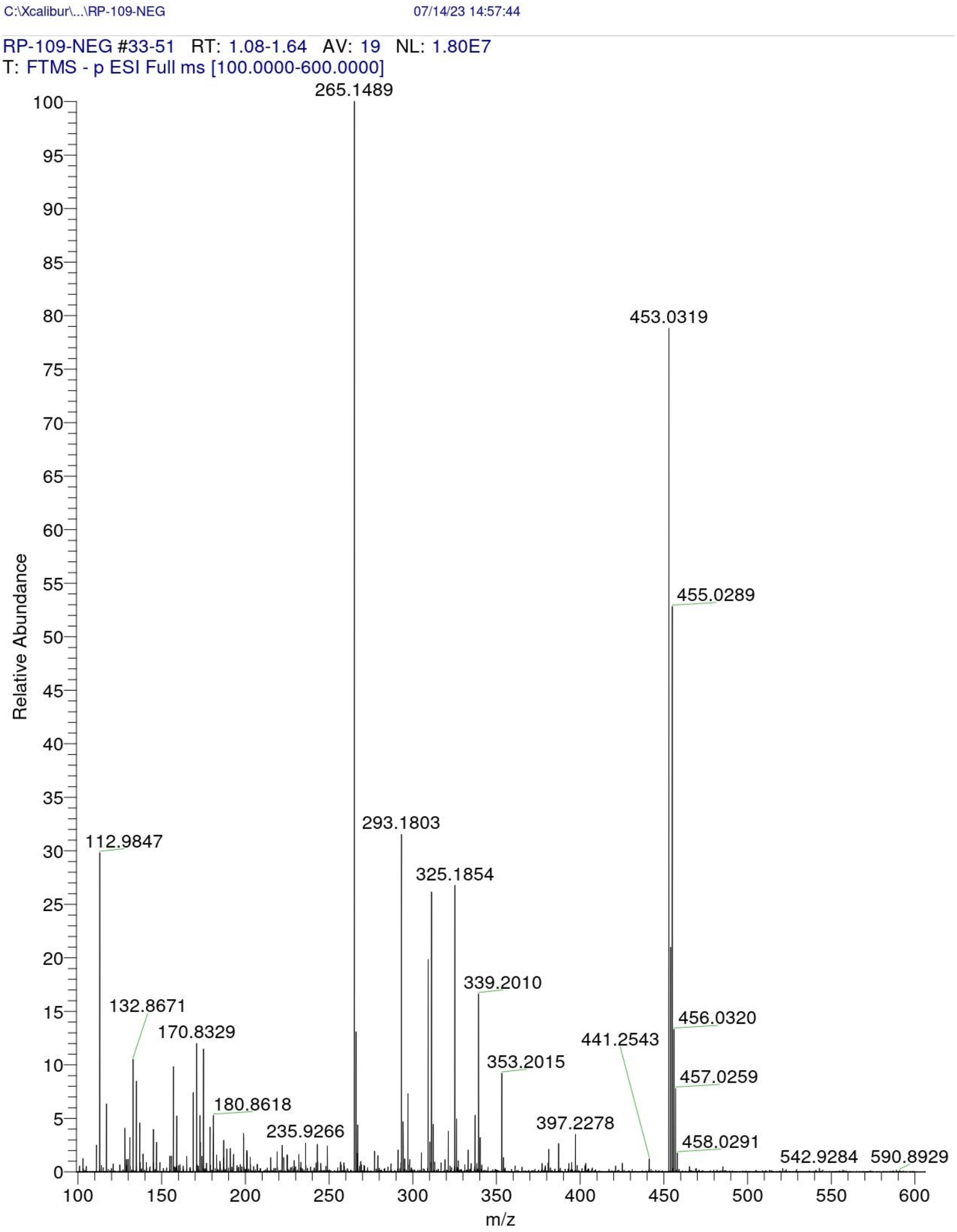

Figure S66: Mass spectrum of compound **30.**

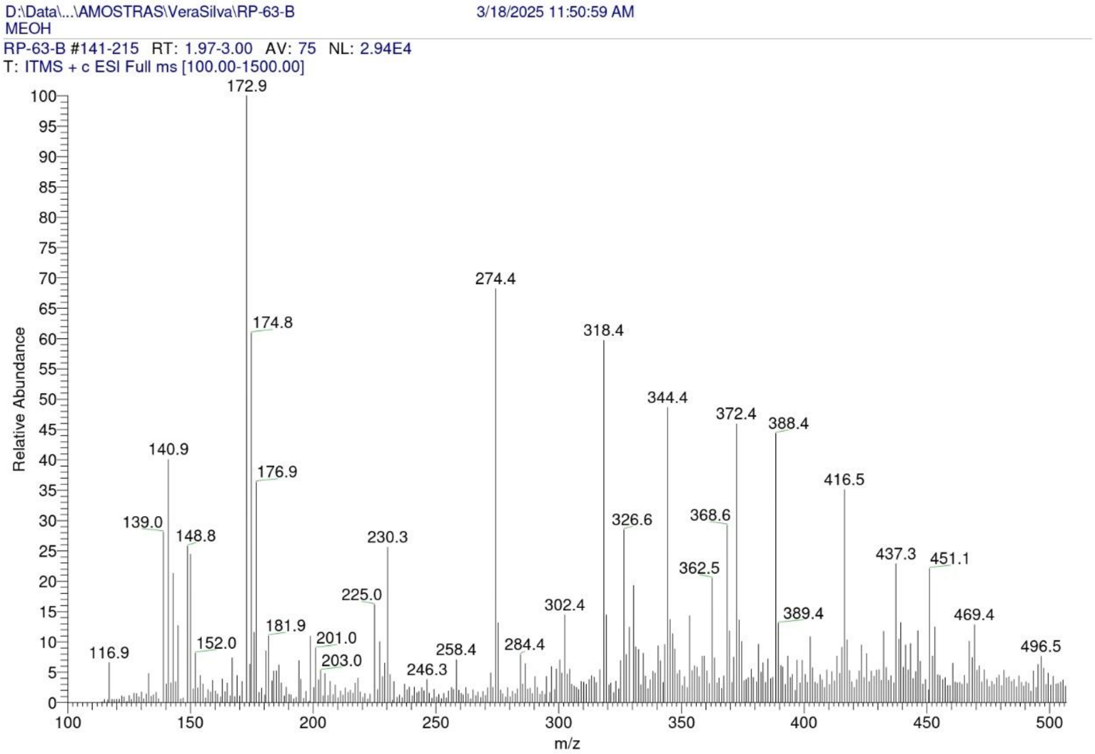

Figure S67: Mass spectrum of compound **31.**

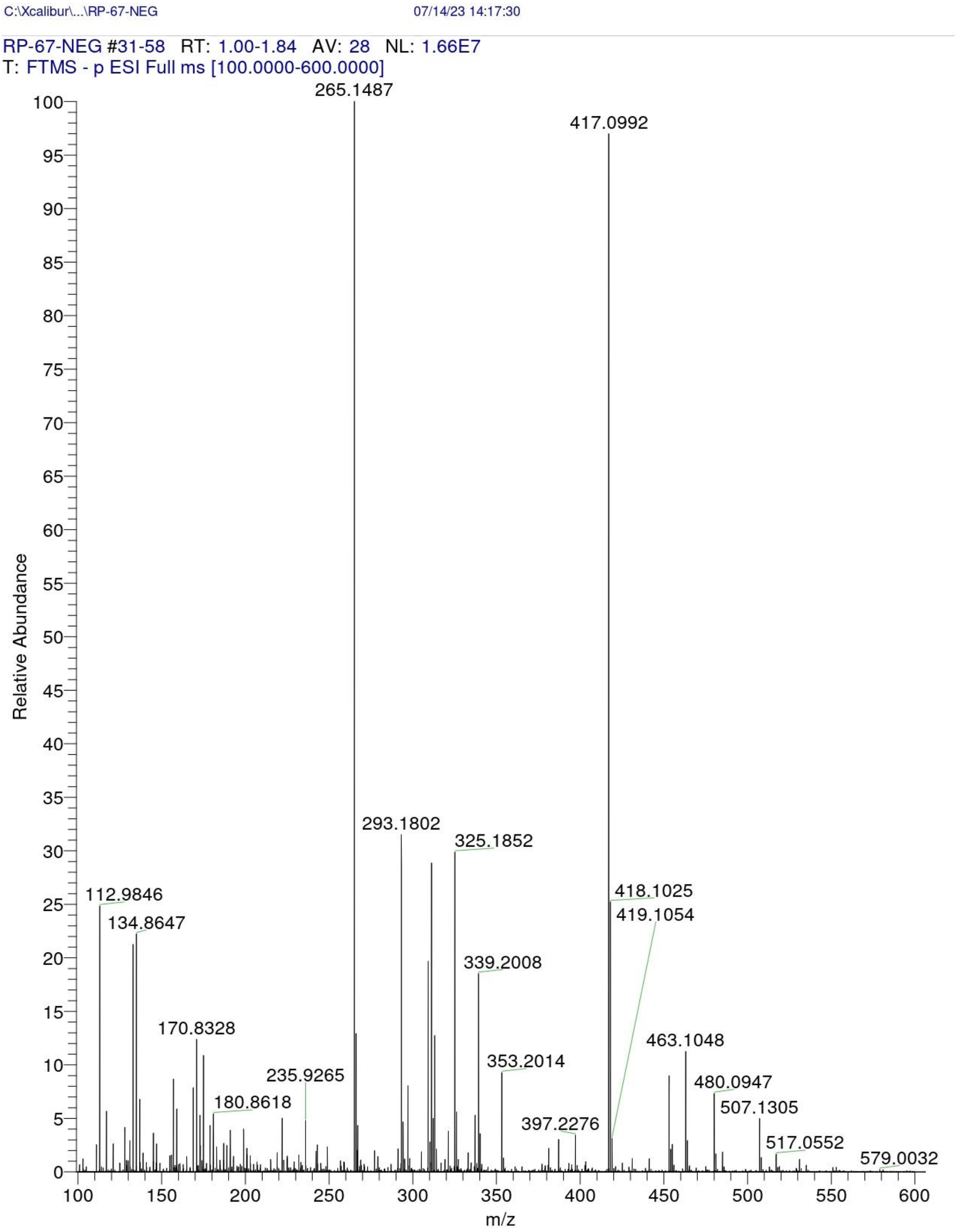

Figure S68: Mass spectrum of compound **32.**

Inhibition studies

Nonlinear regression using Solver

values of x	values of y	concentration of inhibitor (µM)				standard deviation y_{exp}
6,25	4644,76	0	3834,00	4446,71	5653,57	925,81
25	7970,40	0	7905,71	8183,857	7821,643	189,57
100	10479,48	0	10383,71	11129,29	9925,429	607,62
6,25	4009,36	0,195313	3044,14	5131,86	3852,08	1052,71
25	7642,67	0,195313	6957,43	8928,29	7042,286	1114,19
100	8818,57	0,195313	9526,29	8889,00	8040,43	745,43
6,25	2955,48	0,78125	4355,71	2338,43	2172,29	1215,48
25	5857,29	0,78125	4668,00	6121,14	6782,71	1081,77
100	7418,14	0,78125	7997,29	8035,714	6221,429	1036,56
6,25	1353,70	3,125	1193,57	1065,86	1801,67	393,17
25	3121,77	3,125	3704,14	2630,71	3030,44	542,51
100	3760,89	3,125	2566,86	3314,71	5401,11	1468,86
uM		uM				

Figure S69: Mean values of the slopes (y values) and respective standard deviations as results of the in vitro inhibition of COX-2 (2.5 ng/µL) by *bis*-chalcone **31** (0–3.125 µM) using three substrate concentrations (x values: 6,25, 25 and 100 µM).

Without Inhibition

$$v_{inic} = \frac{V_{max} \times |S|}{K_m + |S|}$$

Competitive Inhibition

$$v_{inic} = \frac{V_{max} \times |S|}{K_m \times (1 + \frac{|I|}{K_{ic}}) + |S|}$$

Noncompetitive Inhibition

$$v_{inic} = \frac{V_{max} \times |S|}{K_m \times (1 + \frac{|I|}{K_{ic}}) + |S| \times (1 + \frac{|I|}{K_{iu}})}$$

Uncompetitive Inhibition

$$v_{inic} = \frac{V_{max}(S)}{K_m + (S) \times (1 + \frac{|I|}{K_{iu}})}$$

Mixed Inhibition

$$v_{inic} = \frac{V_{max} \times |S|}{K_m \times (1 + \frac{|I|}{K_{ic}}) + |S| \times (1 + \frac{|I|}{K_{iu}})}$$

Without Inhibition $(y_{exp} - y_{cal})^2$	Ycalc	Competitive Inhibition $(y_{exp} - y_{cal})^2$	Ycalc	Noncompetitive Inhibition $(y_{exp} - y_{cal})^2$	Ycalc	Uncompetitive Inhibition $(y_{exp} - y_{cal})^2$	Ycalc	Mixed Inhibition $(y_{exp} - y_{cal})^2$	Ycalc
7140231,5203	3299,8976	2704158,2528	5219,1903	28948928,6868	7657,7710	2388383,8423	4170,7257	1725160,7059	4584,4650
11015740,6645	6060,4427	176115,6834	8156,8082	348118,9768	7666,9566	81098,1802	8025,8464	221579,3783	8193,7897
24534464,4833	7663,0917	3660625,3827	9492,5235	24430404,4672	7669,2565	743596,6660	#######	969823,4156	10201,7319
3726390,9652	3299,8976	2402129,3115	3760,5301	27330073,3698	6902,6680	2220115,7576	3974,0736	2228716,8864	4073,4862
9993125,6394	6060,4427	3421444,5033	7083,3162	4089063,3834	6910,9479	2779780,7053	7328,0476	2681853,4234	7385,0958
5116727,5299	7663,0917	1335042,4818	9091,6509	12004696,5102	6913,0209	1771456,2628	9287,6596	1719790,9428	9268,9290
3310678,1065	3299,8976	5438970,5586	2045,5000	19825457,8092	5326,8794	3785205,3529	3481,5959	2983173,0170	3052,7268
2464257,0611	6060,4427	4160898,9134	5078,2991	3164218,7197	5333,2691	2346571,0537	5812,0737	2416563,8522	5697,9902
2328924,5635	7663,0917	3421089,5595	8069,3380	15169017,3967	5334,8689	2724437,1107	6980,1506	2211515,8252	7273,7005
11672235,3540	3299,8976	1497735,0417	724,2649	6449487,8375	2784,3537	3155504,2108	2327,7514	396768,5659	1524,5760
26496104,0435	6060,4427	2231883,7859	2381,6647	923447,9174	2787,6936	598934,1024	3180,3564	651217,0814	2977,3339
49996558,1789	7663,0917	14089267,9823	5565,9014	7151583,8163	2788,5298	4517843,9312	3500,9357	4380390,3996	3908,4074
157795438	sum	44539361	sum	149834499	sum	27112927	sum	22586553	sum

Without Inhibition		Competitive Inhibition		Noncompetitive Inhibition		Uncompetitive Inhibition		Mixed Inhibition	
V_{max} =	8403,88	V_{max} =	10040,59	V_{max} =	7670,02	V_{max} =	11599,88	V_{max} =	11109,19
K_m =	9,67	K_m =	5,77	K_m =	0,01	K_m =	11,13	K_m =	8,90
		K_{ic} =	0,24	K_{iu}= K_{ic}=	1,79	K_{iu} =	1,42	K_{ic} =	1,34
								K_{iu} =	2,02

Figure S70: Sum of the squares (sum) of the different models (without inhibition, competitive inhibition, noncompetitive inhibition, uncompetitive inhibition, and mixed inhibition) determined from the results obtained from COX-2 inhibition by *bis*-chalcone **31**.

Models comparison based on F test

	$(y_{exp} - y_{cal})^2$	p	N	N-p	$f_{0,05}$	Fcalc	ΔSS	Δdf	ΔSS / Δdf	p (%)*
Without Inhibition	157795438,1101	2	36	34						
Competitive Inhibition	44539361,4570	3	36	33	1,783	83,91	0,972	0,031	31,102	0%
Noncompetitive Inhibition	149834498,8910	3	36	33	1,788	1,75	5,634	0,031	180,281	0%
Uncompetitive Inhibition	27112927,1760	3	36	33	1,788	159,06	0,200	0,031	6,413	2%
Mixed Inhibition	22586553,4937	4	36	32	1,799	95,78				
	Criterium 1					Criterium 2			Criterium 3	
	Lower ΔSS for the same N-p					Higher Fcalc			Evaluate if mixed inhibition is better than other inhibition	

If Δ SS/Δ df value is higher than $f_{0,05}$ value, mixed inhibition is prefered

Comparison based on Akaike:

	AIC c	ΔAICc			
Without Inhibition	557,2644				
Competitive Inhibition	514,2333	-43,03			
Noncompetitive Inhibition	557,9069	0,64			
Uncompetitive Inhibition	496,3642	-60,90			
Mixed Inhibition	492,4515	-64,81	-21,78	-65,46	-3,91

Figure S71: Comparison of the different models (without inhibition, competitive inhibition, noncompetitive inhibition, uncompetitive inhibition, and mixed inhibition), based on the COX-2 inhibition by *bis*-chalcone **31.**

Uncertainties calculation by the "Jackknife" procedure (95% level of confidance)

Eliminated number	V_{max}	K_m	K_{ic}	K_{iu}
1	11153,66	9,16	1,43	2,00
2	11155,92	8,70	1,25	2,00
3	10688,28	8,09	1,18	2,28
4	11089,60	8,78	1,33	2,02
5	11080,20	9,02	1,36	2,03
6	11426,99	9,52	1,40	1,96
7	11105,44	8,87	1,43	2,00
8	11105,49	8,90	1,28	2,01
9	11093,92	8,87	1,40	1,94
10	11137,44	9,07	1,61	1,95
11	11116,14	8,85	1,26	2,00
12	11031,16	8,74	1,22	2,24

V_{max} error:	108,97
K_m error:	0,23
K_{ic} error:	0,08
	0,07

Figure S72: Error parameters determination (V_{max}, K_m and K_{ic}) for competitive inhibition model of COX-2 by *bis*-chalcone **31**, through "Jackknife" procedure.

Nonlinear regression using Solver

values of x	values of y	concentration of inhibitor (μM)				standard deviation y_{exp}
6,25	3188,24	0	4291,29	2248,57	3024,86	1031,11
25	4049,58	0	4296,14	3366,46	4486,143	599,18
100	4403,71	0	4852,29	3599,29	4759,571	698,20
6,25	1495,93	8	799,00	2184,92	1503,86	693,00
25	2884,92	8	3514,93	2926,29	2213,54	651,68
100	4359,64	8	4387,07	4067,29	4624,57	279,65
6,25	882,74	10	2252,57	178,71	216,92	1186,47
25	2375,57	10	2238,00	3462,57	1426,14	1025,16
100	3776,73	10	2656,62	4987,86	3685,714	1168,28
6,25	507,87	12,5	497,79	541,39	484,43	29,79
25	1706,27	12,5	1835,54	1632,00	1651,29	112,36
100	2926,87	12,5	2718,46	3131,43	2930,71	206,51
uM		uM				

Figure S73: Mean values of the slopes (y values) and respective standard deviations as results of the in vitro inhibition of COX-1 (2.5 ng/μL) by *bis*-chalcone **30** (0–12.5 μM) using three substrate concentrations (x values: 6,25, 25 and 100 μM).

	Without Inhibition		Competitive Inhibition		Noncompetitive Inhibition		Uncompetitive Inhibition		Mixed Inhibition	
	$(y_{exp}-y_{cal})^2$	Ycalc	$(y_{exp}-y_{cal})^2$	Ycalc	$(y_{exp}-y_{cal})^2$	Ycalc	$(y_{exp}-y_{cal})^2$	Ycalc	$(y_{exp}-y_{cal})^2$	Ycalc
	11385286,6501	1431,4507	2126869,6348	3200,9832	3807096,4966	3936,7285	7433581,8106	1858,1754	2126837,7644	3200,5594
	4941168,3978	2863,1120	740472,0290	4136,0721	753108,3298	3941,4507	1056959,5911	3713,4632	742272,7862	4139,4752
	2005299,6982	3817,6699	985123,7676	4461,9332	1612743,3715	3942,6330	1866051,8928	4948,7209	987005,4938	4467,0916
	972957,0482	1431,4507	1458372,5948	1088,5419	4126650,2263	2523,2476	999410,0163	1609,8336	1451493,9651	1091,3659
	850802,7318	2863,1120	1201466,2942	2542,3343	1235251,5999	2526,2742	855865,9525	2838,4072	1197602,1554	2544,2194
	1037615,2081	3817,6699	1040898,1630	3816,6613	10231796,8188	2527,0320	2334157,3923	3507,6361	1051835,7883	3813,3143
	3718665,4814	1431,4507	2823404,3276	934,3838	8973072,2966	2315,4107	4182474,7780	1557,7849	2824233,7223	936,9942
	2814996,1349	2863,1120	2111527,4195	2318,9465	2111786,8177	2318,1881	2380846,0652	2680,4963	2110960,8157	2320,6395
	2734798,6884	3817,6699	2755851,9234	3683,4874	9105707,9438	2318,8835	3501290,5718	3269,6059	2758589,8767	3678,7155
	2560797,0932	1431,4507	247138,9279	793,8530	7599449,0438	2099,2680	2938549,1132	1497,2730	251209,2405	796,2153
	4040067,6275	2863,1120	465727,4325	2089,4535	494537,4071	2101,7861	1944936,5357	2506,2095	469185,1252	2090,9545
	2465875,3277	3817,6699	1174943,5213	3529,5431	2124455,0955	2102,4166	108040,5310	3013,9458	1152357,8119	3523,2644
	39528330	sum	17131796	sum	52175655	sum	29602164	sum	17123585	sum

Without Inhibition:
$$v_{inic} = \frac{V_{max} \times |S|}{K_m + |S|}$$

V_{max} = 4294,98; K_m = 12,50

Competitive Inhibition:
$$v_{inic} = \frac{V_{max} \times |S|}{K_m \times (1 + \frac{|I|}{K_{ic}}) + |S|}$$

V_{max} = 4582,27; K_m = 2,70; K_{ic} = 1,24

Noncompetitive Inhibition:
$$v_{inic} = \frac{V_{max} \times |S|}{K_m \times (1 + \frac{|I|}{K_{ic}}) + |S| \times (1 + \frac{|I|}{K_{iu}})}$$

V_{max} = 3943,03; K_m = 0,01; $K_{iu} = K_{ic}$ = 14,28

Uncompetitive Inhibition:
$$v_{inic} = \frac{V_{max}(S)}{K_m + (S) \times (1 + \frac{|I|}{K_{iu}})}$$

V_{max} = 5565,87; K_m = 12,47; K_{iu} = 17,31

Mixed Inhibition:
$$v_{inic} = \frac{V_{max} \times |S|}{K_m \times (1 + \frac{|I|}{K_{ic}}) + |S| \times (1 + \frac{|I|}{K_{iu}})}$$

V_{max} = 4588,13; K_m = 2,71; K_{ic} = 1,25; K_{iu} = 2554,47

Figure S74: Sum of the squares (sum) of the different models (without inhibition, competitive inhibition, noncompetitive inhibition, uncompetitive inhibition, and mixed inhibition) determined from the results obtained from COX-1 inhibition by *bis*-chalcone **30**.

Models comparison based on F test

	$(y_{exp}-y_{cal})^2$	p	N	N-p	$f_{0,05}$	Fcalc	ΔSS	Δdf	ΔSS / Δdf	p (%)*
Without Inhibition	39528330,0871	2	36	34						
Competitive Inhibition	17131796,0354	3	36	33	1,783	43,14	0,000	0,031	0,015	90%
Noncompetitive Inhibition	52175655,4475	3	36	33	1,788	-8,00	2,047	0,031	65,504	0%
Uncompetitive Inhibition	29602164,2505	3	36	33	1,788	11,07	0,729	0,031	23,320	0%
Mixed Inhibition	17123584,5454	4	36	32	1,799	20,93				
	Criterium 1					Criterium 2			Criterium 3	
	Lower ΔSS for the same N-p					Higher Fcalc			Evaluate if mixed inhibition is better than other inhibition	

If Δ SS/Δ df value is higher than $f_{0,05}$ value, mixed inhibition is prefered

Comparison based on Akaike:

	AIC c	ΔAICc			
Without Inhibition	507,4302				
Competitive Inhibition	479,8375	-27,59			
Noncompetitive Inhibition	519,9300	12,50			
Uncompetitive Inhibition	499,5263	-7,90			
Mixed Inhibition	482,4831	-24,95	2,65	-37,45	-17,04

Figure S75: Comparison of the different models (without inhibition, competitive inhibition, noncompetitive inhibition, uncompetitive inhibition, and mixed inhibition), based on the COX-1 inhibition by *bis*-chalcone **30.**

Uncertainties calculation by the "Jackknife" procedure (95% level of confidance)

Eliminated number	V_{max}	K_m	K_{ic}
1	4560,39	2,36	1,09
2	4616,00	2,68	1,21
3	4634,23	2,82	1,26
4	4641,26	2,89	1,23
5	4604,46	2,80	1,19
6	4400,58	2,23	1,06
7	4575,42	2,68	1,25
8	4587,30	2,71	1,23
9	4555,70	2,62	1,21
10	4548,76	2,61	1,27
11	4541,84	2,61	1,34

V_{max} error:	51,87
K_m error:	0,16
K_{ic} error:	0,07

Figure S76: Error parameters determination (V_{max}, K_m and K_{ic}) for competitive inhibition model of COX-1 by *bis*-chalcone **30**, through “Jackknife” procedure.

Molecular Docking Studies

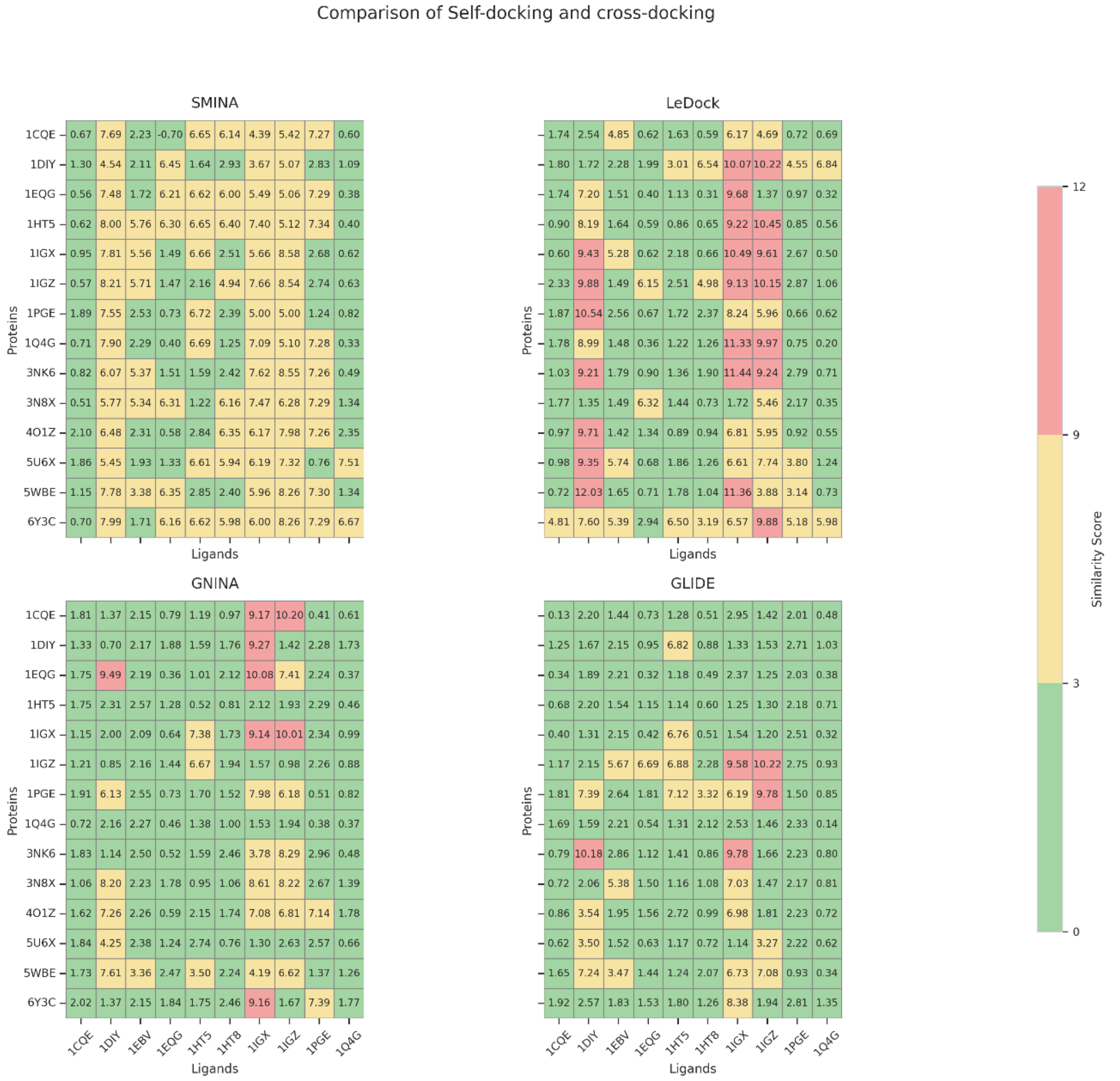

Figure S77: Docking Protocol Validation for COX-1

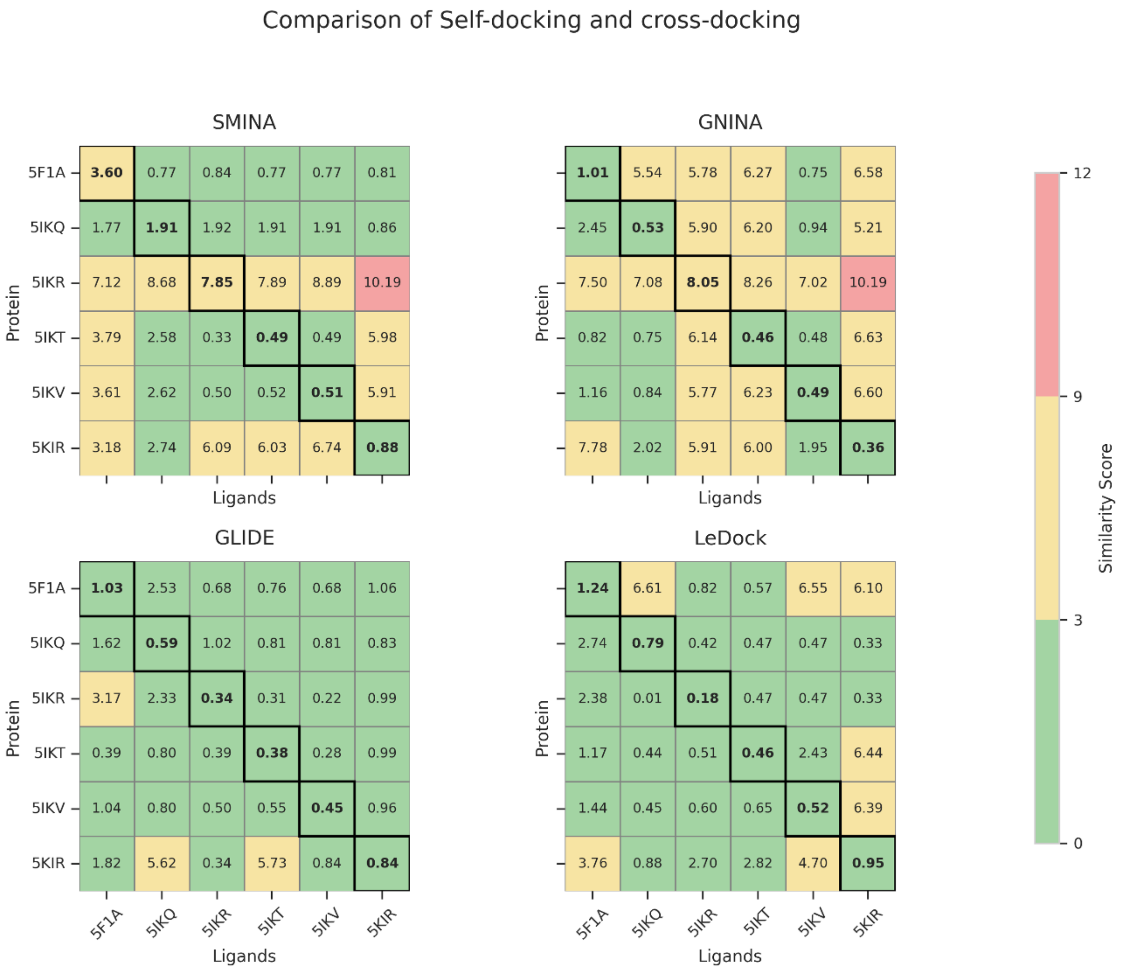

Figure S78: Docking Protocol Validation for COX-2

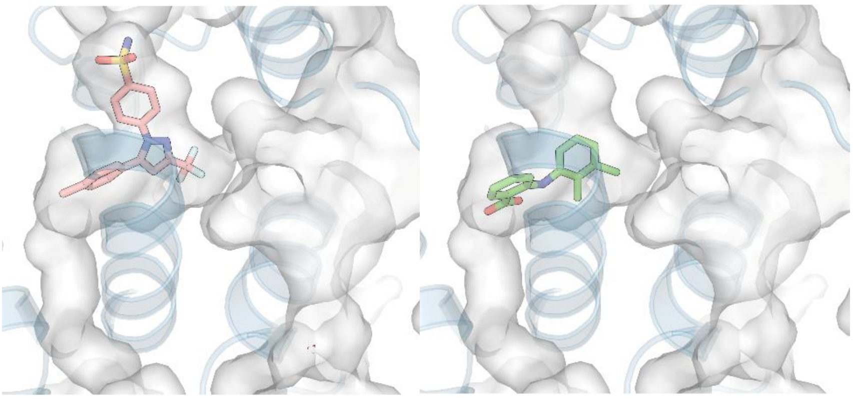

Figure S79: Predicted binding poses for compounds **30** and **31** in COX-1 (PDB 6Y3C) and COX-2 (PDB 5IKT). Both proteins are overlaid, but the surface shown corresponds to COX-2. **Left)** X-ray pose of Celecoxib within COX-2 (PDB 5JW1) occupying the side pocket. **Right)** The X-ray ligand, tolfenamic acid, from the COX-2 structure used in the docking calculations.

Table S1: Molecular properties prediction of compound **30**

Drug-likeness		ADME	
**ID**	**Value**	**ID**	**Value**
**CMC_like_Rule**	Not qualified	**BBB**	2.71084
**CMC_like_Rule_Violation_Fields**	AlopP98_value	**Buffer_solubility_mg_L**	2.63755
**CMC_like_Rule_Violations**	1	**Caco2**	19.8867
**Lead-like_Rule_Violation_Fields**	Molecular_weight, AlopP98_value	**CYP_2C19_inhibition**	Inhibitor
**Lead_like_Rule**	Violated	**CYP_2C9_inhibition**	Inhibitor
**Lead_like_Rule_Violations**	2	**CYP_2D6_inhibition**	Non
**MDDR_like_Rule**	Drug-like	**CYP_2D6_substrate**	Non
**MDDR_like_Rule_Violation_Fields**		**CYP_3A4_inhibition**	Inhibitor
**MDDR_like_Rule_Violations**	0	**CYP_3A4_substrate**	Weakly
**Rule_of_Five**	Suitable	**HIA**	94.919182
**Rule_of_Five_Violation_Fields**	AlopP98_value	**MDCK**	0.0461753
**Rule_of_Five_Violations**	1	**Pgp_inhibition**	Inhibitor
**WDI_like_Rule**	Out of 90% cutoff	**Plasma_Protein_Binding**	100
**WDI_like_Rule_Violation_Fields**	AlopP98_value, AMolRef	**Pure_water_solubility_mg_L**	0.00508899
**WDI_like_Rule_Violations**	2	**Skin_Permeability**	-2.12459
		**SKlogD_value**	6.46014
		**SKlogP_value**	6.46014
		**SKlogS_buffer**	-5.23709
		**SKlogS_pure**	-7.95166

Table S2: Molecular properties prediction of compound **31**

Drug-likeness		ADME	
**ID**	**Value**	**ID**	**Value**
**CMC_like_Rule**	Qualified	**BBB**	0.111495
**CMC_like_Rule_Violation_Fields**		**Buffer_solubility_mg_L**	249.66
**CMC_like_Rule_Violations**	0	**Caco2**	16.4317
**Lead-like_Rule_Violation_Fields**	Molecular_weight, AlopP98_value	**CYP_2C19_inhibition**	Inhibitor
**Lead_like_Rule**	Violated	**CYP_2C9_inhibition**	Inhibitor
**Lead_like_Rule_Violations**	2	**CYP_2D6_inhibition**	Non
**MDDR_like_Rule**	Drug-like	**CYP_2D6_substrate**	Non
**MDDR_like_Rule_Violation_Fields**		**CYP_3A4_inhibition**	Inhibitor
**MDDR_like_Rule_Violations**	0	**CYP_3A4_substrate**	Weakly
**Rule_of_Five**	Suitable	**HIA**	55.02039
**Rule_of_Five_Violation_Fields**	No_H_bond_donors	**MDCK**	0.0516369
**Rule_of_Five_Violations**	1	**Pgp_inhibition**	Inhibitor
**WDI_like_Rule**	Out of 90% cutoff	**Plasma_Protein_Binding**	100
**WDI_like_Rule_Violation_Fields**	No_H_bond_donors	**Pure_water_solubility_mg_L**	1.0524
**WDI_like_Rule_Violations**	1	**Skin_Permeability**	-2.93258
		**SKlogD_value**	-2.93258
		**SKlogP_value**	4.38758
		**SKlogS_buffer**	4.38758
		**SKlogS_pure**	-3.25625
